# A multimeric pore complex in the poxvirus core revealed by integrative structural analysis

**DOI:** 10.1038/s41467-026-79013-7

**Published:** 2026-10-30

**Authors:** Jiasui Liu, Simon Schäfer, Svenja Lehnung, Lukas Max Baumann, Lars Mühlberg, Cornelia Steiner, Markus Schreiber, Mark Linder, Sonja Welsch, Fan Liu, Boris Bogdanow, Jacomina Krijnse Locker, Beata Turoňová

**Affiliations:** 1 Department of Molecular Sociology, Max Planck Institute of Biophysics, Frankfurt am Main, Germany; 2 Division of Immunology, Paul Ehrlich Institute, Langen, Germany; 3 Microscopy of Pathogens, Paul Ehrlich Institute, Langen, Germany; 4 Department of Structural Biology, Leibniz-Forschungsinstitut für Molekulare Pharmakologie (FMP), Berlin, Germany; 5 Institute of Virology, Charité Universitätsmedizin Berlin, Freie Universität Berlin and Humboldt-Universität zu Berlin, Berlin, Germany; 6 IMPRS on Cellular Biophysics, Frankfurt am Main, Germany; 7 Central Electron Microscopy Facility, Max Planck Institute of Biophysics, Frankfurt am Main, Germany; 8 Charité Universitätsmedizin Berlin, Berlin, Germany; 9 Cluster of Excellence SubCellular Architecture of Life (SCALE), Goethe University Frankfurt, Frankfurt, Germany

**Keywords:** Pox virus, Cryoelectron tomography

## Abstract

It has long been known that poxviruses transcribe more than a hundred early mRNAs within their viral core, yet how these transcripts exit the core remains unknown. Here, we combine cryo-electron tomography with deep-learning-based particle picking, subtomogram averaging, cross-linking mass spectrometry, and AlphaFold3 modeling to resolve the molecular architecture of a long-sought pore within the poxvirus core. The pore is formed by a heteromeric assembly of the viral proteins E8, E6, and L3, which together create a hollow hexameric tube spanning both the core wall and the surface-exposed palisade layer. Within the pore channel, we observe a rod-like density consistent with the viral RNA helicase I8, which may contribute to the release of the newly synthesized viral mRNAs from the core. We further observe a state of the pore lacking the outer and basal rings under transcription conditions. Together, our findings identify the structure and dynamics of a candidate mRNA-release pore within the surface layer of a double-stranded DNA virus and suggest that it may form part of the poxvirus transcription machinery.

## Abstract (web summary)

Poxviruses transcribe mRNA inside a sealed core, but how it escapes was unknown. Using cryo-electron tomography and integrative modelling, the authors resolve a pore in the core wall, built from three viral proteins, that may release the mRNA.

## Introduction

Poxviruses are a family of double-stranded DNA viruses that replicate in the cytoplasm of the host cell. Their virions have a distinctive asymmetric structure with a brick-shaped membrane surrounding a biconcave core flanked by two lateral bodies. Poxviruses cause diseases such as Mpox as well as smallpox, by the now-eradicated Variola virus. The recent Mpox outbreaks, declared by the WHO as a public health emergency of international concern, underscore the importance of studying poxviruses^1^. The prototype member vaccinia virus (VACV) is a valuable model for understanding the biology and structural organization of poxviruses^2^.

Poxviruses exist in two major infectious forms: the mature virion (MV), also known as the intracellular mature virus (IMV), and the extracellular enveloped virion (EV)^3^. The virus enters the host cell via fusion of the viral envelope with host membranes, releasing the viral core into the cytoplasm. This membrane-free, brick-shaped core, with dimensions of roughly 200 × 320 x 200 nm, contains the double-stranded DNA genome along with the viral transcription machinery. The latter allows for the transcription of more than 100 early mRNAs inside the core, which are then released into the cytoplasm for translation by the host translational apparatus^4,5^. The early viral proteins trigger the uncoating of the viral core, followed by release of the genome into the cytoplasm, initiating DNA replication followed by virus assembly^2^.

The structure of the MV is only partially understood due to its size, compactness and asymmetrical shape. To facilitate structural analyses, we recently analyzed membrane-free cores by cryo electron tomography (cryo-ET) and subtomogram averaging (STA). These cores are composed of at least three distinct layers. The innermost layer has a filamentous appearance and appears discontinuous. It is surrounded by a continuous layer likely composed of dimers of the major core protein A3^6^. The outer surface of the core consists of typical spike-like structures, known as the palisade layer. The central part of the core appears sparsely filled with electron-dense material, likely composed of the viral transcription machinery. The core surface is covered by the palisade layer, composed of stake-like projections that were recently shown to consist of trimers of the A10 protein^6,7^. Importantly, interspaced between the abundant A10-trimers, ring-like or pore-like structures were observed and proposed previously to serve as mRNA release sites during the early stages of infection^8^ composed of a hexameric, hollow structure, surrounding a central density. They are typically surrounded by six A10-trimers, giving them a flower-like appearance^6,7,9^. It has been proposed that E6 hexamers are a constituent of this structure, but a conclusive structure remains unavailable due to resolution limitations.

To overcome this problem, we developed a high-throughput particle picking pipeline by combining techniques of template matching (TM), deep-learning based surface segmentation, oversampling, and STA. We applied this optimized pipeline to VACV cores, which enabled efficient and accurate particle localization, allowing us to resolve the structure of the pore complex at ~8.2 Å resolution. Leveraging this structure, we integrated Alphafold3 (AF3)^10^ and cross-linking mass spectrometry to demonstrate that the complex is a heteromeric assembly composed of hexamers of the viral gene products E8 and E6, and a dodecamer of L3, with transcription-state-dependent dynamics. We further propose that the rod-like density in the pore lumen consists of the viral RNA-helicase I8, potentially contributing to mRNA release.

Collectively, our findings describe a strong candidate for the mRNA-release machinery of poxviruses, whose existence has been postulated for decades. By defining its protein composition, we uncover a multimeric assembly with the hallmarks of a pore, providing a channel through which mRNA could be exported across the core wall. Consistent with such a role, all of its constituents have previously been implicated in poxvirus early transcription. This work advances our understanding of the structural basis of early gene expression in poxviruses and opens new avenues for investigating the regulation, evolution, and vulnerability of this unique transcriptional system.

## Results

### VACV cores undergoing transcription are analyzed by cryo electron tomography

During virion assembly VACV packages its transcription machinery inside the core to mediate early transcription immediately after entry and delivery of the core into the cytoplasm. Viral early transcription can be mimicked in vitro by incubating membrane-free cores with NTPs (ref- Moss 1991). Biochemical data supported the notion that viral mRNAs are synthesized inside such cores and then released in an ATP-dependent manner. We prepared membrane-free cores as before^7^, mixed them with transcription mix and immediately pipetted the mix onto holey grids inside the plunge freezer. After 5 minutes of incubation inside the plunge freezing chamber kept at 37 degrees, the mix was plunge-frozen. UV/VIS-spectrometry of RNA isolated from viral cores in the presence and absence of UTP detected an increase in RNA concentration, implying that the cores undergo transcription under these conditions (Supplementary Fig. 1).

**Fig. 1:**
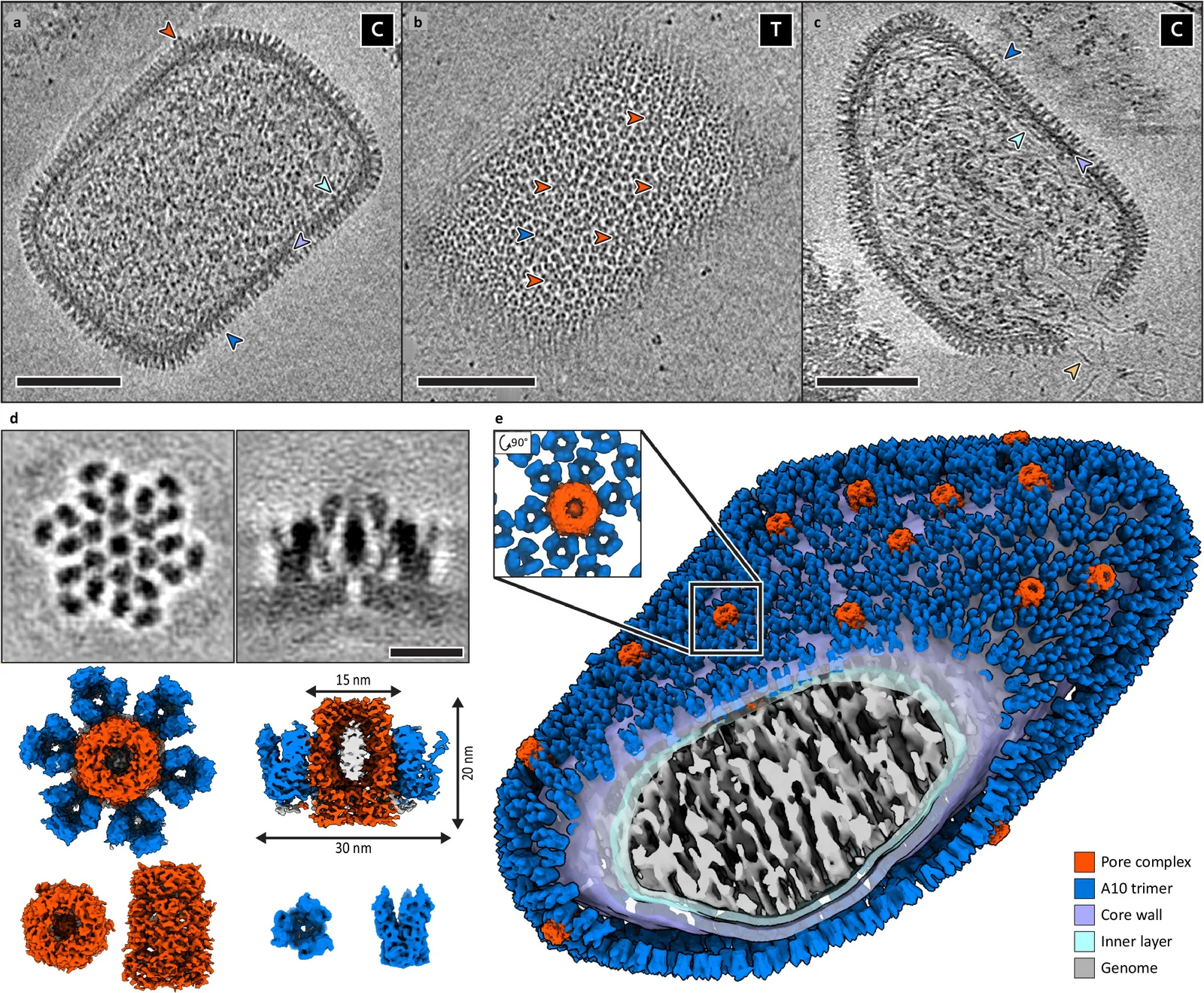
Organization of the VACV core during transcription in vitro, as revealed by tomography. Tomogram slices showing a central (**a**) and top (**b**) view of an intact VACV core during early transcription. **c** Tomogram slice showing a central view of a ruptured VACV core during early transcription, with filamentous density (beige arrowhead), possibly DNA, exiting the core**. d** STA maps of the palisade components. The top and middle rows show the STA structure of the pore complex, surrounded by six A10 trimers, with the top view on the left and the side view on the right. The STA map (top) shows central slices, while the iso-surface representation (middle) shows the full top view (left) and a side view cut through the center of the pore complex (right). The bottom row shows iso-surface representations of the STA structures of the pore complex (left) and the A10 trimer (right). The complex forms a portal-like structure approximately 15 nm in diameter and 20 nm in height. **e** A 3D view of the in vitro transcribing VACV core organization, showing the pore complex (dark orange) and A10 trimers (blue) forming the palisade layer, together with the segmented core wall (purple), inner layer (light blue), and internal genome (beige). The enlarged image in the top-left corner shows details of the pore complex surrounded by A10 trimers. Scale bars: 100 nm (**a**–**c**), 15 nm (**d**).

A total of 89 tilt series were collected from in vitro transcribing VACV cores. The reconstructed tomograms revealed a core architecture similar to that of the membrane-free cores^7^ hereafter referred to as untreated cores. The cores exhibited a brick-like shape with a surface composed of three layers: the palisade layer, the core wall, and an inner layer (Fig. 1a–c, Supplementary Video 1). Interestingly, unlike in the untreated condition, these cores displayed detached lateral bodies and a decondensed genome inside (Supplementary Fig. 2), which appeared as filaments filling most of the core center (Fig. 1a). Two distinct states of the core were observed. In one state, the core remained intact and exhibited substantial internal density, which appeared relatively evenly distributed (Fig. 1a and Supplementary Video 1). In the other state, the core-wall had ruptured, allowing filamentous density, potentially DNA, to exit (Fig. 1c). These ruptured cores appeared overall less filled, and the internal density was formed by filaments interspaced with distinct granular densities, rather than the uniform distribution seen in intact cores (Fig. 1a, c). The palisade layer, however, appeared unchanged when comparing transcriptionally active versus untreated cores. When viewed from the top, the pore-like structures were recognizable by the presence of six surrounding A10-trimers (red arrowheads in Fig. 1b).

### A high-throughput pipeline for automated pore complex picking

**Fig. 2:**
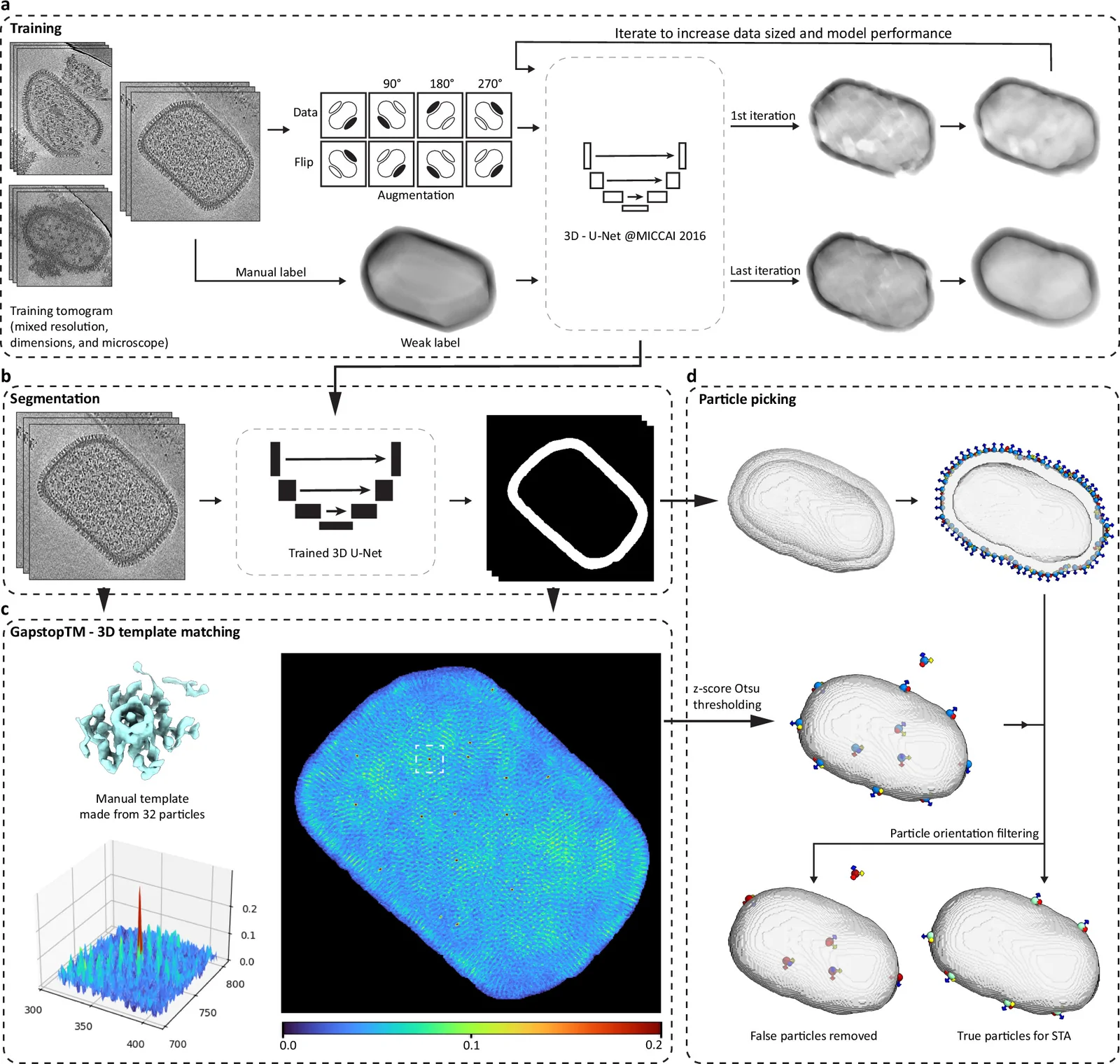
Particle localization workflow. The particle-picking workflow consists of four steps. **a** Training. The 3D U-Net was trained on fSIRT bin8 tomograms of varying dimensions and pixel sizes, each augmented eightfold through rotations and flips. A weak label was first generated by constructing a convex-hull shell from manually annotated boundary points on the core surface and used for the first training round; the resulting predictions were refined with CryoCAT and manually corrected in Dragonfly to generate an improved ground truth for a second training round. **b** Segmentation. Only the network from the final iteration was used to predict the palisade segmentation for all tomograms. These segmentations were subsequently used both to mask the GAPSTOP^TM^ template-matching results and to generate surface triangle meshes for contextual particle cleaning. **c** GAPSTOP^TM^ 3D template matching. A subtomogram average of 32 manually picked pore complexes was used as the reference for template matching (top left). Several significant peaks were detected within the palisade layer, corresponding to pore-complex positions (right and bottom left); the 40 top-scoring peaks per core were extracted as particle candidates with associated positions and orientations. **d** Particle picking. A triangle mesh was generated from the palisade segmentation using the marching cubes algorithm (step size = 5) and sampled into a surface point cloud. Each particle candidate from template matching was compared with the nearest surface-point normal, and candidates whose orientations differed by more than 90° from that normal were discarded.

Because of the low abundance of pore complexes on VACV cores, large numbers of tomograms are needed to obtain sufficient particles for STA, and manual identification of side-view pores embedded in the A10 layer is labor-intensive and subjective. We therefore developed an automated workflow (Fig. 2; “Methods”) that integrates deep-learning segmentation, template matching, and surface-geometry-based particle cleaning.

First, a trained 3D U-Net segments each VACV core. From each segmentation, a surface mesh is generated, and from it a point cloud describing the core surface, with each point carrying both its spatial coordinates and its local surface orientation (normal).

Candidate pore positions are then located across the dataset by high-confidence 3D template matching (TM) using GAPSTOP^TM^^11,12^. Masking the TM results with the corresponding core segmentation suppresses background from the carbon edge and yields distinct peaks, which are detected by Otsu-based peak detection in cryoCAT^13^. This first use of the segmentation restricts candidates to the core surface and enable reliable detection of pore complexes across many tomograms, including a substantial number of side-oriented pores that would have been difficult to localize manually.

The resulting candidates are refined by STA, after which the surface point cloud provides a geometric reference for a second cleaning step: each particle is compared to the nearest surface point, and those whose orientations deviate by more than 90° from the local surface normal are inconsistent with the core surface and are excluded. This surface-normal filtering removes spurious hits while retaining genuine pores in all orientations.

### The pore complex is a hexameric tube supported by six A10 trimers

In total, 2,678 particles from 119 cores were obtained for refined STA. The initial reconstruction included not only the pore complex but also the six surrounding A10 trimers and reached a resolution of 9.4 Å (Fig. 1d). Six A10 trimers contact the outer surface of the pore complex, giving it its characteristic flower-with-petals appearance (Fig. 1b, d). The pore complex measures approximately 20 nm in height and 15 nm in diameter, or ~30 nm in diameter when including the six surrounding A10 trimers (Fig. 1d). A distinct rod-like density is visible in the center of the complex and spans a substantial portion of its vertical axis. The pore complexes are randomly distributed within the palisade, without apparent higher-order organization, with an average of 23 complexes per core (Fig. 1e).

Structurally, the complex can be divided into three regions (Fig. 1d). The top part protrudes about 4 nm above the A10 layer and forms a prominent ring. The middle region is aligned with the A10 layer and has a height of approximately 10 nm, similar to that of the A10 trimers; the hollow part of this region contains a prominent electron-dense central rod-like structure (Fig. 1d). The base extends about 6 nm below the A10 layer and is integrated into the core wall, potentially corresponding to the A3 protein^6^. By penetrating the core wall, the hollow central channel of the complex creates an opening to the interior of the core (Fig. 1d top left).

Visual inspection of the reconstruction revealed a clear hexameric symmetry of the tubular pore complex, which we further validated by an in-plane angle randomization test (Supplementary Fig. 3, Methods). After confirming this architecture, C6 symmetry was applied during refinement. Applying a tighter mask to focus on the pore complex further improved the resolution to 8.1 Å (Fig. 1d, Supplementary Fig. 4).

The A10 trimers were processed using the same workflow, resulting in reconstructions of all trimers as well as a subset containing only the surrounding trimers, with resolutions of 7.7 Å and 9.7 Å, respectively (Fig. 1d, Supplementary Fig. 6). For details on STA see the Methods, and for the Fourier shell correlation (FSC) curves see Supplementary Fig. 5.

### Identification of pore complex components by cross-linking mass spectrometry

**Fig. 3:**
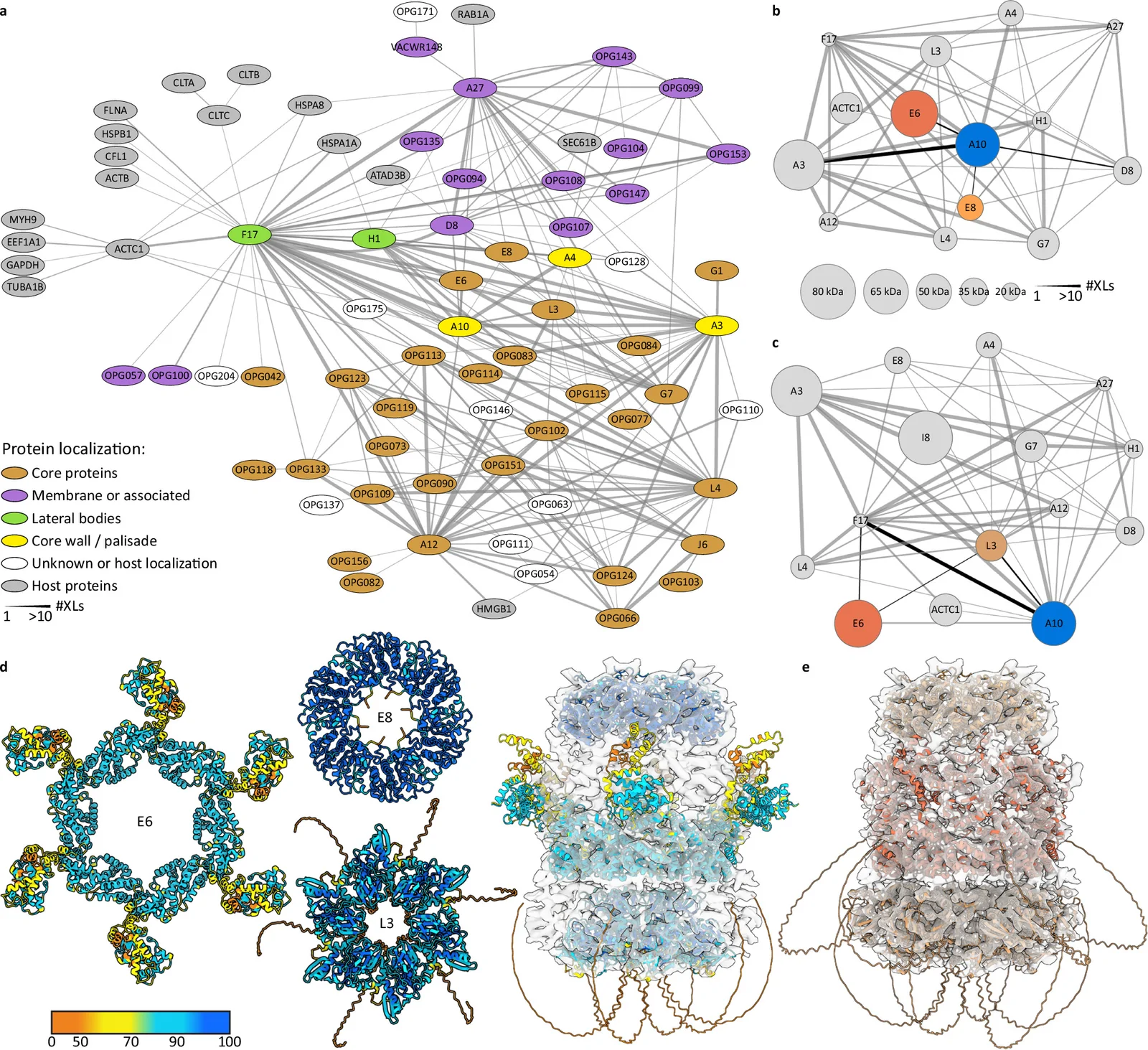
VACV core interactome network captured by Cross-Linking Mass Spectrometry. **a** VACV core interactome network at 1 % FDR on PPI level. Annotated protein localization in the virion is represented by node color. Width of the edges is correlated with the number of detected cross-links. **b** Extracted cross-linking network of proteins interacting with A10, from which candidate pore components were drawn. Node size correlates with molecular weight. The components ultimately assigned to the pore complex are highlighted (E6, orange; E8, apricot). **c** Extracted cross-linking network of VACV proteins cross-linking to both A10 and E6, from which candidates for the basal ring were drawn; L3, the component assigned to the basal ring, is highlighted in cappuccino. **d** AF3 predictions of the E8, E6, and L3 hexamers. The interface predicted template modeling (ipTM) scores were 0.87, 0.4, and 0.73, respectively. Left and middle panels show top views of the individual hexameric predictions, while the right panel shows the side view of the predicted models fitted into the STA map. Whereas E8 and the lower portion of E6 agree well with the density, the upper region of the E6 protomers extends beyond the map, and the L3 hexamer alone does not fully account for the basal density. The model is colored by per-residue confidence (predicted Local Distance Difference Test; pLDDT), with values indicated on the scale bar. **e** Final model of the pore complex after rigid-body fitting of E6 domains and density-guided placement of additional L3 protomers to complete the basal assembly, shown fitted into the STA map. Source data are provided as a Source Data file.

To identify candidate proteins forming the pore complex, we performed cross-linking mass spectrometry (XL-MS) of VACV cores using the chemical cross-linker disuccinimidyl sulfoxide (DSSO). Analysis of two biological replicates revealed substantial overlap at the level of protein-protein interactions (PPIs) (Supplementary Fig. 6). Overall, we identified 87 proteins, forming 233 PPIs linked by 3,349 cross-linked residue pairs. The data capture predominantly known components of the core and palisade layer, but lateral body proteins and membrane proteins are also present (Fig. 3a).

We reasoned that the pore components should cross-link to the surrounding A10 trimers and therefore examined the A10 cross-linking partners as candidate pore components (Fig. 3b; Supplementary Table 1). Some candidates were set aside without re-prediction because experimental structures are available and are incompatible with the pore density: A27, a membrane protein of the viral envelope, and H1, a dual-specificity phosphatase that assembles as a dimer. Actin was excluded as a host protein. Because a smaller protein could in principle contribute to the density through a higher oligomeric state, the remaining candidates were not filtered by molecular mass but were instead predicted with AF3 across all oligomeric states, from monomer to dodecamer (Supplementary Table 2). Predictions compatible with the six-fold symmetry of the pore density (i.e., six subunits or multiples thereof) were subjected to a global search against the density to determine their best position and orientation (see “Methods”). Although some non-hexameric states, including pentamers, returned high AF3 scores, the reconstructed pore is six-fold symmetric throughout (Supplementary Fig. 3), and we found no evidence of pentameric assemblies in the cryo-ET data. Of all candidates examined, only a few gave placements compatible with the density: a hexamer of E6 in the inner region, L3 in the basal region, and A3 and E8 in the upper region. The remaining candidates were positioned in orientations incompatible with the density at any oligomeric state tested (Supplementary Fig. 7).

E6 is required for proper assembly of MVs^14,15^, and a previous study suggested that the pore complex could be assembled from E6^9^. Consistent with this, a hexamer of E6 gave the best placement in the inner region of the pore (Supplementary Table 3). The lower portion of the E6 hexamer matched the middle part of the STA structure well, whereas its upper portion extended beyond the observed pore density (Fig. 3d, Supplementary Fig. 8), supporting E6 as a component of the pore complex. The density extending above the palisade layer and the basal structure piercing the putative A3 core wall indicated that at least two further proteins are part of the complex.

L3 cross-links to both A10 and E6 (Fig. 3c) and is an essential factor for early transcription, as L3-deficient virions are impaired in early viral transcription^16^. Our cross-linking analysis further revealed interactions between L3 and A3, which has been proposed to form the core wall^6^, providing indirect support for a core-wall–associated location of L3. A hexameric L3 prediction, fitted into the bottom region of the STA structure (Fig. 3c, Supplementary Fig. 9), accounted well for the central portion of the basal density, but additional surrounding density remained unassigned. This remaining density could be explained by placement of further L3 protomers, suggesting that L3 forms a higher-order, double-ring assembly (Fig. 3e, Supplementary Fig. 9). Attempts to predict this arrangement directly with AF3 did not yield a consistent dodecameric model, producing instead overlapping or stacked hexamers (Supplementary Fig. 10); the basal architecture was therefore modeled in a density-guided manner. To test whether the density supports a six-fold double-ring, we expanded a single L3 protomer under C2-C12 symmetry and fitted each to the basal density (Supplementary Table 3). Only expansions commensurate with six-fold symmetry (C2, C3, C6) placed subunits into density, whereas the remaining symmetries fitted markedly worse; among these, the six-fold expansion filled all lobes, and the six individually placed protomers occupied them with comparable cross-correlation. The intra-L3 cross-links are compatible with this compact arrangement. We therefore present the basal double-ring as the model best consistent with the density and cross-linking data rather than as a uniquely determined structure.

Among the candidates, a hexamer of the E8 gene product (32 kDa) gave the best agreement with the upper density (Fig. 3d, Supplementary Fig. 11; Supplementary Table 3). E8 is essential for transcriptional competence, as a temperature-sensitive E8 mutant fails to synthesize early mRNA at the non-permissive temperature^17^. A3 was also examined, but owing to its larger size it overfilled the density and fitted markedly worse than E8. The cross-links between A3 and L3 place it at the basal core-wall side rather than the top of the pore, and together with its proposed role in the core wall^6^ this assigns A3 to the core wall rather than the pore.

### Topology and domain organization of the pore complex

**Fig. 4:**
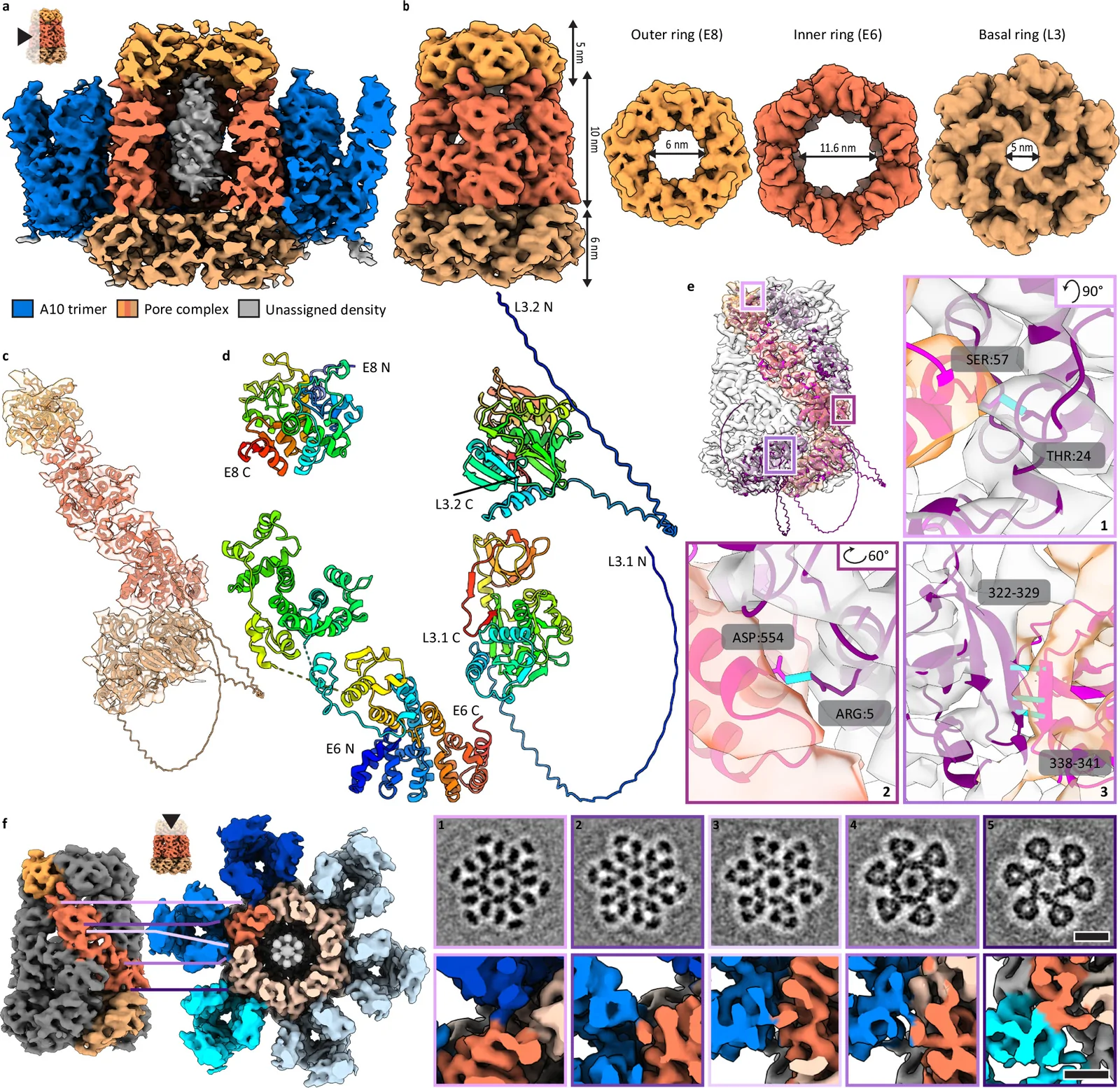
The pore complex forms a hexagonal portal composed of three layers. **a** Side view of the pore complex subtomogram average (STA), with components colored separately: A10 trimers (blue), outer ring (apricot), inner ring (orange), and basal ring (cappuccino), and unassigned density (gray). **b** A composite map of the pore complex showing the heights of the rings (left). The top views of the rings are presented on the right together with their approximate inner diameters, which correspond to the largest point within each ring. The color code of the components is as in (**a**). **c** A single chain of the pore complex model fitted into a sharpened STA map. **d** The individual protomers shown separately, each colored with a rainbow gradient indicating the N- (navy blue) and C- (red) termini. **e** Two neighboring chains fitted into a sharpened STA map, highlighting the interfaces numbered 1–3 from top to bottom. (1) The interface between two E8 protomers, formed by a polar interaction network centered on Ser56/Ser57, which engages Asp178, Thr176 and Thr24 through multiple hydrogen bonds (shown in cyan). (2) The interface between domain 2 of two E6 protomers, containing an electrostatic cluster involving Asp554 and Arg5/Arg6. (3) The interface within the inner L3 ring, in which a β-hairpin (strands 322–329 and 338–341) from one protomer is augmented by a strand from its neighbor; the associated polar contacts are shown as hydrogen bonds (cyan). **f** Depiction of a single chain from each ring within the pore complex (left) linked to the connections between the A10 trimers and the inner ring as seen from the top (middle). The corresponding slices from the STA maps are shown on the top right, while the bottom right shows the corresponding zoomed-in iso-surfaces. Five connections between E6 and the surrounding A10 trimers are resolved in the density, spanning three different A10 trimers. Scale bars 15 nm (top) and 2.5 nm (bottom).

These analyses indicate that the pore complex is composed of three ring-shaped structural elements, hereafter referred to as the outer, inner, and basal rings (Fig. 4a, b). The inner diameter of the complex ranges from ~2.8 nm to ~11.6 nm, with the largest radius in the inner ring and the smallest in the basal ring. The prominent rod-like density is confined to the inner ring of the complex (Fig. 4a).

To model the pore complex as a single assembly, we first used AF3 to predict arrangements of the candidate components E8, E6, and L3, including models in which L3 was represented as a dodecameric double ring. Although these predictions correctly placed E8, E6, and L3 in the same vertical order suggested by the STA structure, they did not yield a satisfactory model for the basal L3 assembly. In particular, the predicted L3 dodecameric arrangements were inconsistent, whereas the relative placement of the three components was broadly compatible with the density. We therefore used an AF3 model containing three candidate hexamers (E8, E6, and L3) as an initial scaffold for the overall organization of the complex. In this initial model, the predicted spacing between the rings was considerably smaller than observed in the STA map (Supplementary Fig. 12a). In addition, the E6 hexamer remained partially open, with its upper portion extending beyond the density, indicating that the AF3 prediction was incomplete. This was not specific to a single prediction: across 500 predictions from 100 independent seeds, AF3 consistently generated six-fold-symmetric E6 hexamers but never the closed conformation seen in the density (no model within 12 Å Cα RMSD of the fitted model; median 24.7 Å; Supplementary Fig. 13, Supplementary Table 4; Methods), indicating that ring closure is not captured by prediction of the isolated hexamer.

To improve the model, we performed crosslink-guided modeling using AF3x^18^. Incorporating the cross-linking constraints produced assemblies in which the E6 hexamer engaged the E8 hexamer located above it, resulting in a closed E6 ring and a defined interaction interface between the two proteins. Although this model better satisfied the cross-linking data, the spacing between the hexameric layers still remained smaller than observed in the STA reconstruction (Supplementary Fig. 12a, b). The closed E6 ring nonetheless improved the fit to the density, with the cross-correlation rising from 0.37 to 0.51 for the full complex and from 0.48 to 0.63 for the E6 hexamer alone, and the model overflow correspondingly reduced (Supplementary Table 5).

Closer inspection of E6 revealed two flexible regions at the boundaries between residues Lys163-Lys164 and Asn409-Glu410, delimiting three segments along the sequence—consistent with the three blocks that were apparent in the predicted aligned error (PAE) plots (Supplementary Fig. 12a). The N- and C-terminal segments (up to Lys163 and from Glu410 onward) both form the lower part of the E6 hexamer and fit the STA density well without modification, whereas the middle segment extends upward, beyond the observed density in the AF3 predictions.

For fitting, we therefore treated the protein as two rigid bodies: domain 1, the middle segment that extends beyond the density, and domain 2, the N- and C-terminal segments that together form the lower portion and fill in the density as predicted (Fig. 3e, Supplementary Fig. 12d). Domain 1 from the AF3x model and domain 2 from the AF3 model were positioned into the STA map using Assembline (Rantos et al. 2022), with an elastic network maintaining connectivity across the segment boundaries. The E8 hexamer was fitted independently into the outer density region. For L3, the AF3-derived hexamer accounted for the inner basal ring, whereas the remaining basal density was filled by density-guided placement of additional AF3-derived L3 protomers to form an outer concentric ring. This procedure yielded a model that closely matches the overall dimensions and spacing observed in the STA reconstruction (Fig. 3e, Supplementary Fig. 12d). Among the models tested, the final fitted model gave the best agreement with the density (cross-correlation 0.59 for the full complex and 0.68 for the E6 hexamer; Supplementary Table 5). The fitted arrangement of four connected protomers—one each from E8 and E6 and two from L3 representing the inner and outer basal rings—is shown in Fig. 4c. The resulting model reveals a clear architectural organization of the pore complex.

The resulting model reveals a clear architectural organization of the pore complex. The E8 hexamer forms the outer part of the complex and protrudes above the A10 trimer layer. The N-terminus of each E8 monomer points toward the center of the assembly, whereas the C-terminus lies at the base of the hexamer (Fig. 4d). Neighboring E8 protomers associate through a polar interaction network involving highly conserved residues (Fig. 4e, panel 1; Supplementary Table 6), consistent with a functionally constrained oligomerization interface. The channel inside the E8 hexamer displays a strong positive electrostatic potential (Supplementary Fig. 14).

The E6 hexamer is situated directly beneath E8 and is embedded within the palisade layer. Domain 1 of E6 is positioned between pairs of E8 monomers. The interface is formed by an acidic helical segment of E6 whose acidic character is conserved across sequences, facing a region of the E8 C-terminus carrying conserved basic residues, indicating charge complementarity at this interface (Supplementary Fig. 15, Supplementary Table 6). In contrast to other regions of the pore complex, the channel formed by domain 1 carries strong negative electrostatic potential (Supplementary Fig. 14). Domain 1 also contacts neighboring E6 protomers. Domain 2 is positioned directly below and contains both the N- and C-terminal regions of the protein (Fig. 4d). Here, the N-terminus of each monomer contacts the C-terminus of its neighbor through an interface centered on conserved basic and acidic residues (Fig. 4e, panel 2). This inter-protomer arrangement is independently supported by cross-links within the domain-2/C-terminal region whose distances exceed the ~35 Å DSSO limit within a single protomer and therefore report contacts between neighboring subunits (Supplementary Table 6).

The L3 assembly forms the basal part of the pore complex and is embedded within the core wall beneath the palisade layer. Consistent with the density-guided modeling described above, L3 adopts a dodecameric organization composed of two hexameric rings (Supplementary Fig. 9). The inner ring (L3.1) lines the central channel, narrow at its top and flaring outward toward its base. The outer ring (L3.2) sits on this flared region, radially outside the upper part of L3.1 while extending above it to form the interface with the E6 hexamer. The N-terminal region of L3, up to Gly65, lacks ordered density in both rings (Fig. 3e, Supplementary Fig. 9); cross-linking nevertheless indicates that it is not simply disordered, as residues within it cross-link to their own equivalents in neighboring protomers, which can only occur between subunits (Supplementary Table 6).

Within the inner ring, where neighboring protomers are in close contact, a three-stranded β-sheet forms between them: a β-hairpin from one protomer augmented by a strand from its neighbor (Fig. 4e, panel 3). The interface is supported by conservation and by an inter-protomer cross-link (Supplementary Table 6, Supplementary Fig. 16a). To test the sensitivity of the predicted β-sheet in silico, we introduced substitutions into the paired strands in AlphaFold3. The proline substitution of the paired strands abolished the predicted assembly, whereas β-compatible valine substitutions at the same positions, or proline substitutions in a remote loop, left it unchanged (“Methods”, Supplementary Fig. 16b–d, Supplementary Table 7). No equivalent pairing occurs in the outer ring, whose larger radius separates the protomers too widely for strand augmentation; L3.2 instead interfaces with E6 through hydrophobic packing involving conserved nonpolar residues. The central channel of the L3 assembly shows strong positive electrostatic potential (Supplementary Fig. 14).

At the interface between the pore complex and the surrounding A10 trimers, contacts to A10 are observed exclusively from the E6 ring. Notably, a single E6 protomer spans interactions with three distinct A10 trimers (Fig. 4f). Cross-links between E6 and A10 satisfying the DSSO distance constraint further confirm the proximity of the two proteins (Supplementary Table 6).

Finally, to independently evaluate the plausibility of the model, we mapped cross-linked residue pairs onto the structure while excluding low-confidence regions (pLDDT <50). Because the DSSO cross-linker spans distances of up to ~35 Å, we assessed whether the observed cross-links satisfied this constraint. Of the 100 cross-links mapped onto the model (excluding low-confidence regions, pLDDT <50), 75 were within the ~35 Å Cα–Cα distance compatible with DSSO. Of the 25 exceeding this distance, 16 are A10-A10 cross-links, which do not necessarily originate from the pore complex and may arise from elsewhere in the palisade; excluding these, 75 of 84 cross-links (89%) satisfy the distance constraint, supporting the overall validity of the model. A summary of the evidence supporting each interface—conservation and cross-linking restraints—is provided in Supplementary Table 6, and the evidence supporting each component assignment is summarized in Table 1.

**Table 1:** Evidence supporting the assignment of each pore-complex component

Component & assigned region	Density fit	XL-MS support	AF3 / AF3x confidence	Prior biological evidence	Limitations	Pubmed
E8 Outer ring	Hexamer gave the best agreement with the upper density; alternative size-comparable candidate (D8) did not fit at any oligomeric state tested	Cross-links to A10 (basis for candidate selection); inter-protomer E8-E8 links exceed the 35 A DSSO limit within one protomer	Hexamer predicted with consistent inter-protomer interfaces	Essential for transcriptional competence; ts mutant L81F fails to synthesize early mRNA at non-permissive temperature	No direct E6-E8 cross-link detected; the E6-E8 interface is supported by conserved charge complementarity rather than by a distance restraint	17619043
E6 Inner ring	Hexamer gave the best placement in the inner region; fit improved from CC 0.48 (AF3 prediction) to 0.68 (fitted model), with model overflow reduced from 0.41 to 0.19	Cross-links to A10 (basis for candidate selection); inter-protomer E6-E6 links in the domain-2/C-terminal region exceed the 35 A DSSO limit within one protomer and therefore require a hexameric arrangement; four E6-A10 links satisfy the distance constraint	Monomer predicted with high confidence; isolated hexamer is open (ipTM ~0.4), closing only with cross-link-guided prediction (AF3x) and density fitting	Required for MV assembly; ts mutant P226L incapable of in vitro transcription at non-permissive temperature	Isolated AF3 hexamer does not reproduce the closed conformation (0/500 seeds within 12 A of the fitted model); closure inferred to be lattice-templated; domain-1/domain-2 boundaries defined from predicted flexible regions	20116821 19217136 38171004
L3 Basal ring (inner and outer ring)	Hexamer accounted for the inner basal ring; remaining basal density filled by density-guided placement of further protomers; symmetry expansion supports a six-fold-commensurate arrangement	Cross-links to both A10 and E6 (basis for candidate selection); same-residue L3-L3 links are inter-protomer by definition, supporting the homo-oligomeric arrangement	Hexamer predicted reliably; dodecameric double ring not reproduced by AF3 (overlapping or stacked hexamers of approximate D6 symmetry)	Essential for early transcription; L3-deficient virions produce only trace amounts of early viral mRNA	Outer ring built in a density-guided manner rather than predicted; presented as the model best consistent with the density and cross-linking data rather than as uniquely determined; N-terminal region (to Lys65) lacks ordered density	16282472
I8 Tentative rod density	Monomer compatible with the dimensions of the rod; overall shape and position agree with the density, with deviations in the lower, widened portion	Single I8-L3 cross-link, mapping to residues consistent with the modeled position ( ~ 16 A Ca-Ca); three intramolecular I8 cross-links consistent with a folded monomer	Monomer predicted with high confidence (pTM 0.92); oligomeric states from dimer to dodecamer score poorly (ipTM 0.19-0.35) and did not fit the rod	RNA helicase (NPH-II) required for early transcription; sedimentation properties indicate a monomer; proposed to be required for mRNA release from the core	Rod density not resolved beyond overall shape despite focused classification; assignment rests on shape, size and a single cross-link; presented as a plausible candidate rather than a firm assignment	1332061 8970979 1332061

For each proposed component, the table summarizes the fit to the STA density, the supporting cross-linking restraints, the confidence of the AlphaFold3 (AF3) predictions, prior biological evidence, and the limitations of the assignment. Cross-linking entries state the restraints relevant to the assignment and the oligomeric state; the full interaction network is shown in Fig. 3. The central rod is assigned tentatively, as indicated.

### Classification reveals an uncapped pore state under transcription conditions

To determine whether transcription affects the structure of the pore complex, we compared pore complexes from in vitro transcribing cores with those from untreated cores. For this analysis, we combined a previously published dataset of untreated VACV cores (EMPIAR-11674^7^) with a newly acquired dataset of untreated cores prepared using the same protocol. In total, the merged datasets comprised 100 tomograms containing 131 cores, which were processed using the same workflow described above for the transcription dataset, yielding a total of 2590 particles.

STA of the untreated cores yielded structures of the pore complex together with the surrounding A10 trimers at a resolution of 9.8 Å, the isolated pore complex at 8.2 Å, the asymmetric unit at 8.2 Å, and the A10 trimer surrounding the pore at 11.3 Å (Supplementary Fig. 17). Overall, these reconstructions closely matched those obtained from the transcription dataset in both overall architecture and dimensions, and no clear structural differences were observed (Supplementary Fig. 18).

**Fig. 5:**
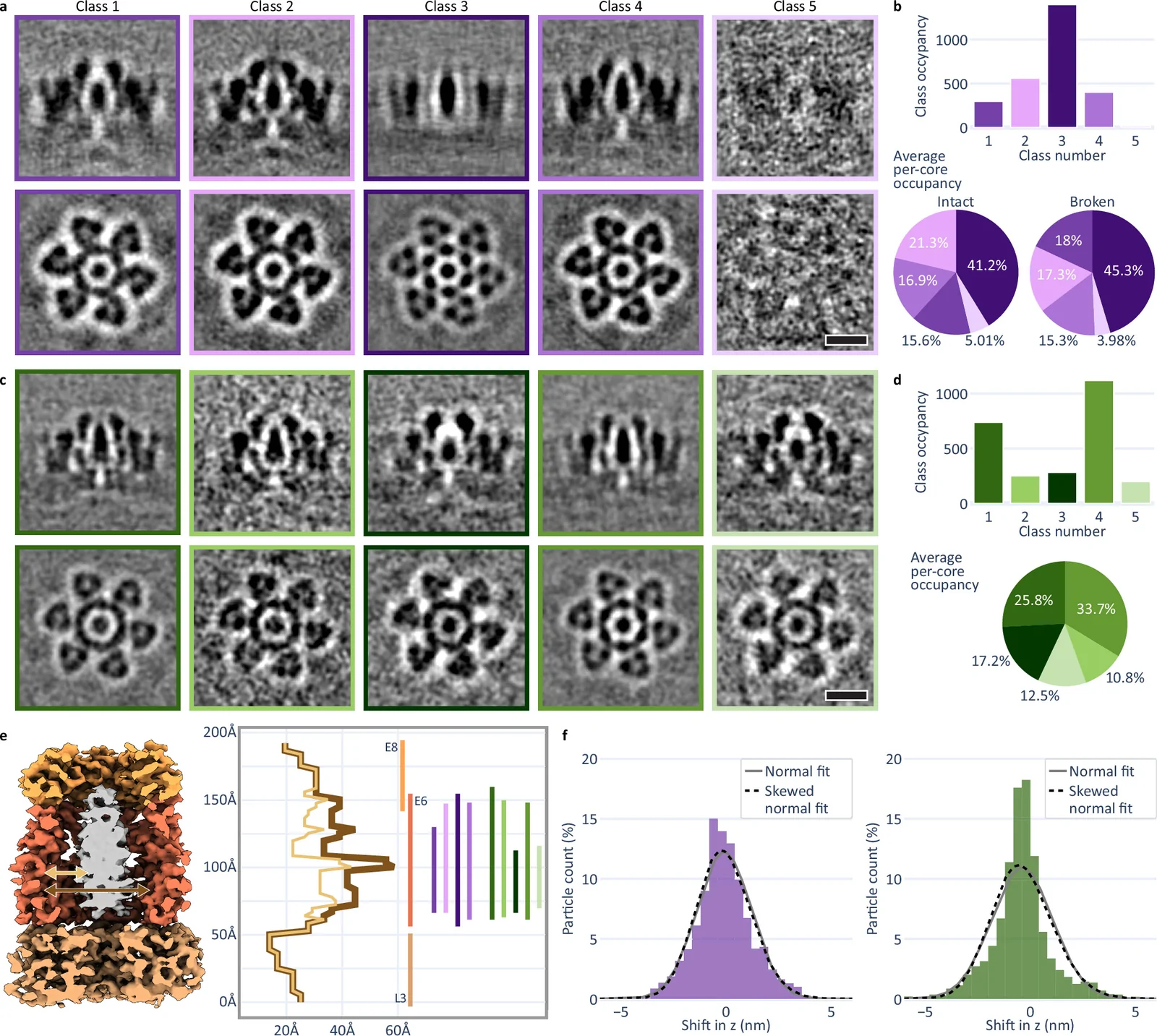
Classification and rod analysis of transcription and untreated condition. **a** De novo classification of the pore complex during transcription. The classes 1, 2, and 4 differ mostly in the shape of the internal rod density while class 5 consists mostly of false positive picks. Interestingly, class 3 misses the outer ring (hexamer of E8) as well as the basal ring (hexamer of L3). **b** Occupancy of the classes from (a) (top) and the average per-core occupancy for intact and broken cores (bottom), showing comparable class distributions and no enrichment of the uncapped class on broken cores (*n* = 119 cores). Classes 1–5 comprise 298, 560, 1,397, 402 and 21 particles, respectively. **c** De novo classification of the pore complex without transcription. Similar to (**a**), the classes differ mostly in the size and shape of the inner rod density. However, all classes contain all three rings. **d** Occupancy of the classes from (**c**) (top) and the average per-core occupancy (bottom) showing random distribution of the classes per core (*n* = 131 cores). Classes 1–5 comprise 738, 251, 283, 1,118 and 200 particles, respectively. **e** The inner channel radius profile of the pore complex is shown on the right, calculated with (beige) and without (brown) inclusion of the rod density. The minimum radius of the pore complex is 13.9 Å and is located at the top of the basal ring. Within the E6 and partially E8 region, where the rod density is present, inclusion of the rod density yields a minimum radius of ~22 Å. The density map on the left is vertically aligned with the radius profile, allowing direct correspondence between structural features and channel dimensions. The graph additionally indicates the vertical positions and height of individual rings (colored as in the map) as well as of the inner rod for different classes (color-coded as in panels (**a**) and (**c**)). **f** Axial position of the inner rod, determined by focused STA, for the transcription (left) and untreated (right) conditions (*n* = 2678 particles, transcription; *n* = 2590, untreated). The distributions are comparable in spread (SD 1.31 and 1.46 nm; no significant difference, two-sided Brown–Forsythe W = 0.008, df = 1, *p* = 0.93), reflecting continuous positional variability of the rod along the pore axis in both conditions. Source data are provided as a Source Data file.

Having established that the overall pore complex architecture remains unchanged between untreated and transcriptionally active cores, we next performed de novo classification to explore whether alternative structural states might be present in either of the two conditions. The classification revealed a distinct class that consistently appeared only under the specific in vitro transcription conditions used here. This class (#3) was characterized by the absence of both the outer and basal parts, corresponding to proteins E8 and L3 (Fig. 5a). We refer to this structure as the uncapped pore complex. In total, 1,397 uncapped pore complexes were detected, representing almost half of the pore complexes observed under these conditions (Fig. 5b).

By mapping the class back to tomograms, individual uncapped pore complexes could be directly observed, confirming the loss of the outer part under in vitro transcription conditions (Supplementary Fig. 19). Interestingly, spatial distribution analysis revealed no significant localization preference for the uncapped complexes; instead, uncapped and intact pore complexes appeared interspersed within the same cores, with the uncapped class showing no preference for broken over intact cores (Fig. 5b, Supplementary Fig. 20).

### The prominent rod-like central density is variable in position

Under both transcription and untreated conditions, the prominent electron-dense rod-like structure filled the central part of the inner ring. It displays a predominant oval shape of approximately 9 nm in height (Fig. 5a, c). Classification also revealed shorter rods, reduced by up to 1.7 nm from the top while remaining anchored at their base (Fig. 5a, c, d, e). The diameter of the rod of roughly 3.4 nm at the base remained largely unchanged. By leaving a gap of at least ~2 nm between the rod and the surrounding pore wall (Fig. 5d, e), it does not seem to close the channel and is geometrically compatible with the passage of single-stranded RNA.

To investigate the behavior of the rod, STA focused specifically on the rod density was performed. Comparison of particle coordinates and orientations between the rod-focused and full pore reconstructions showed that the rod remains strongly aligned with the central axis of the pore. Polar angle analysis revealed that the vast majority of rods adopt orientations with θ close to 0° or 180°, indicating that the rod does not substantially tilt away from the pore axis but primarily rotates around it (Supplementary Fig. 21a). Analysis of translations along the z-axis showed comparable distributions in the transcription and untreated conditions (Fig. 5f; SD 1.3 and 1.5 nm, no significant difference in spread), indicating continuous positional variability of the rod along the pore axis in both conditions rather than a transcription-specific change. To characterize this variability, particles were grouped based on the axial shifts obtained from rod-focused alignment and re-averaged in the context of the full pore. In all cases, the resulting averages displayed a continuous, full-length rod, with only minor positional offsets along the pore axis (Supplementary Fig. 21b). The distribution of axial shifts within individual pore classes was broad and did not reveal class-specific enrichment (Supplementary Fig. 21c). Together, these observations indicate that the apparent shortening of the rod in some classes arises from positional heterogeneity rather than a discrete structural state.

Despite classification and focused STA, the reconstruction of the isolated rod density did not reveal additional structural features. The lack of a well-defined structure suggests that the rod may be structurally heterogeneous, asymmetric, or that its symmetry does not match that of the hexameric pore complex, which would prevent coherent averaging.

**Fig. 6:**
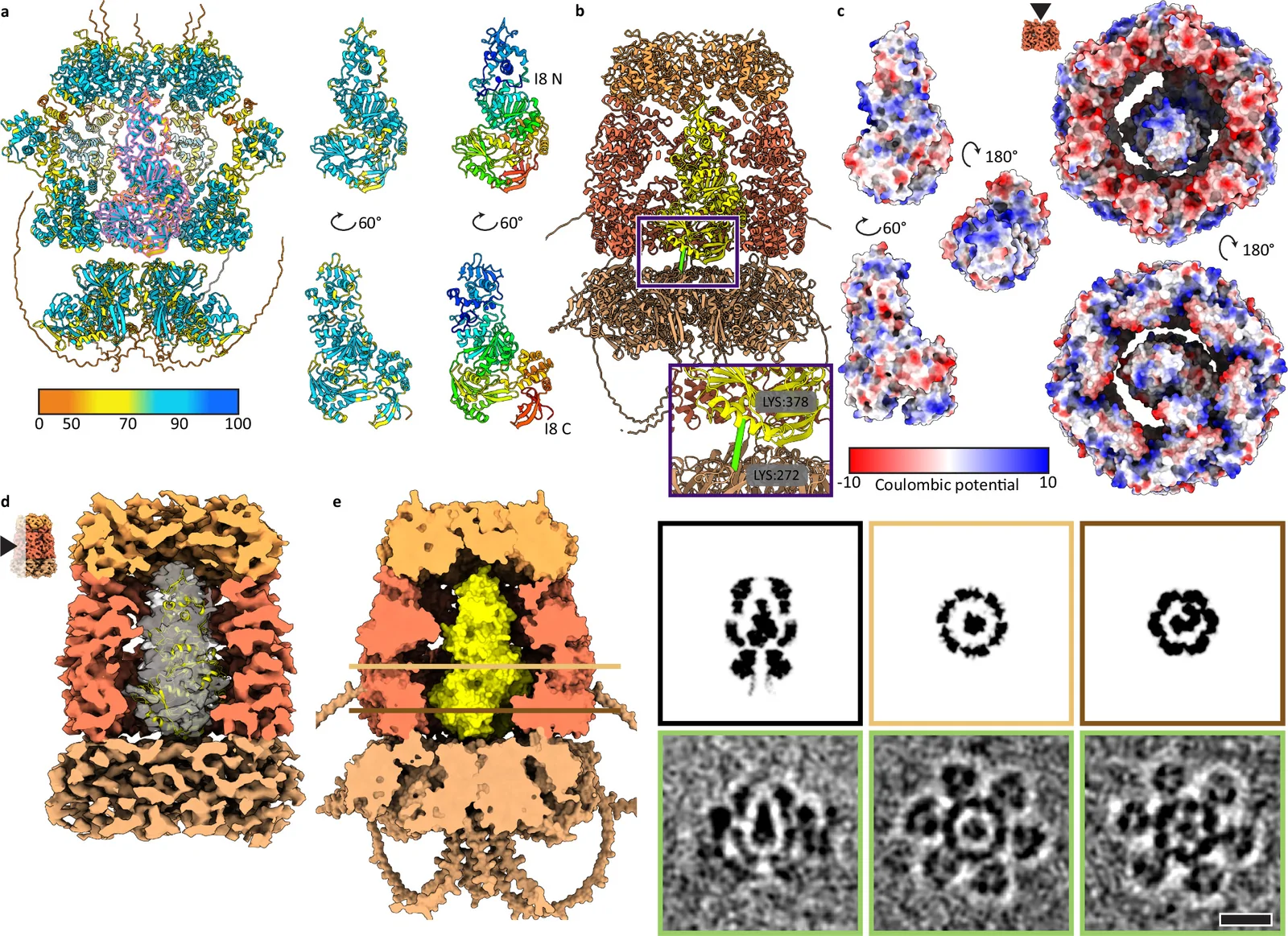
AlphaFold3 prediction of I8 as a possible candidate for the inner rod. **a** Left: AF3 prediction of the pore complex with one I8 monomer highlighted in pink; colors follow the AF3 per-residue confidence (pLDDT), with values indicated on the scale bar. The ipTM is 0.41. Middle: side view of I8 (top) and a 60° rotation about the vertical axis (bottom), illustrating the shape of its basal region. Right: the same views colored by a rainbow gradient from the N terminus (blue) to the C terminus (red). **b** The predicted AF3 model was aligned to our structure (colored as before: outer ring, apricot; inner ring, orange; basal ring, cappuccino) to position the I8 model (yellow) within the assembly. The inset shows the single cross-link between I8 and L3, mapping to residues 378 (I8) and 272 (L3). The corresponding Cα–Cα distance in the model ( ~ 16 Å) is well within the expected range for DSSO cross-links, supporting a potential interaction between the two proteins. **c** The Coulombic surface potential of I8 alone and within the E6 ring highlights complementary electrostatic features between the two assemblies. **d** The I8 prediction fitted into the STA map of the pore complex shows good agreement in overall shape and dimensions. **e** Surface representation of the pore complex with I8 (left). A 10 Å molmap (UCSF ChimeraX) generated from the model is shown as central slices in side and top views (top right), with top-view slices vertically aligned and color-coded to match positions indicated on the model. For comparison, corresponding slices from class 2 of the untreated condition are shown below, where the inner rod most closely resembles the generated map. While the central region agrees well, the lower region – where I8 widens –shows clear shape differences in class 2 (and all other classes).

Nonetheless, to explore possible molecular candidates for this density, we examined cross-linking interactions involving proteins that could plausibly reside within the lumen of the pore complex. Considering only cross-links mapping to residues oriented toward the lumen across components of the outer, inner, and basal rings, only two candidates were identified: F17 and I8, based on their cross-links with L3. We then tested these candidates, together with further proteins associated with early transcription, by predicting them alongside the assigned pore components (hexamers of E8, E6 and L3) and examining where each was placed. Although H1 had been set aside as a candidate for the pore rings, it was included here as a possible lumenal component; predicted as a dimer, it was placed outside the pore, as was F17 (predicted as six chains). L4 was tested as a monomer, dimer and trimer; all three were positioned within the pore but none accounted for the rod density, differing from it in position, shape or size. H6 was placed within the lumen but is substantially smaller than the density. Only I8 was consistently positioned within the lumen, along the central axis, at a size compatible with the rod (Fig. 6a), including in models incorporating RNA (Supplementary Fig. 22). Given the limited resolution of the rod density, this comparison assesses position and overall size rather than detailed fit. All predictions are available at Zenodo^19^.

I8 is an RNA helicase involved in early transcription and is reported to function as a monomer^20^; consistent with this, AF3 predictions of higher I8 oligomeric states scored poorly (Supplementary Table 8). In the AF3 model, the I8–L3 cross-link maps to residues 378 (I8) and 272 (L3), which are separated by ~16 Å (Cα–Cα), consistent with the expected range for DSSO cross-links. Notably, the predicted surface electrostatics of I8 show strong positive charge, which would complement the negatively charged inner surface of the E6 hexamer lining the pore (Fig. 6c, Supplementary Fig. 14). Alignment of the AF3 model within the pore complex positions I8 along the central axis, where its overall shape and dimensions agree well with the observed rod-like density (Fig. 6b, d). Comparison of model-derived density with STA classes further shows good agreement in the central region, while deviations in the lower, widened portion indicate that the observed rod does not fully match the predicted conformation (Fig. 6e). Together, these observations suggest that I8 represents a plausible candidate for the dynamic rod located within the pore complex lumen.

### Pore architecture in intracellular cores is consistent with the structure obtained in vitro

**Fig. 7:**
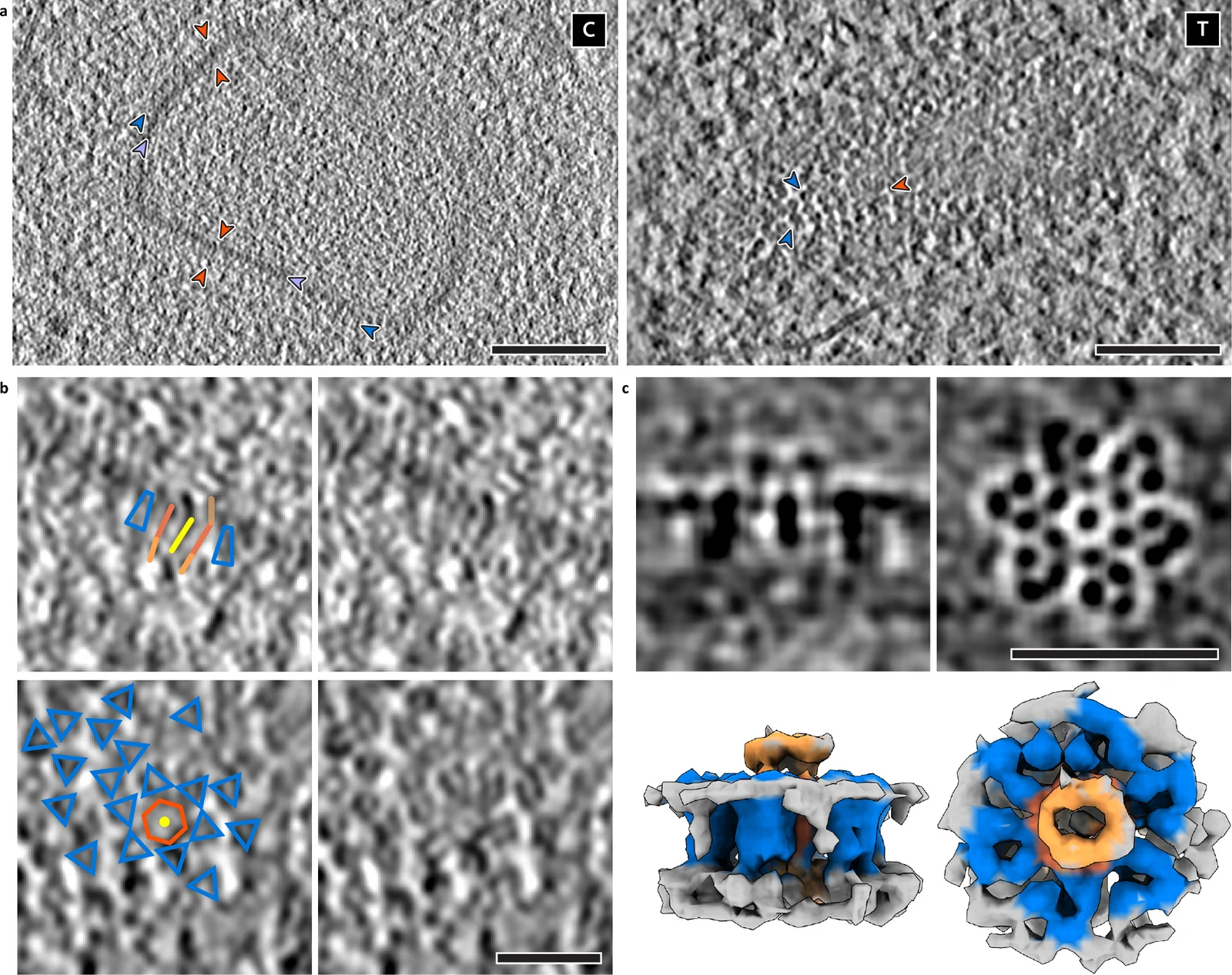
In situ cryo-electron tomography of vaccinia virus cores in infected cells. **a** Central (left) and top (right) views of in situ viral cores. Arrows highlight key structural features, including the palisade layer containing A10 trimers (blue), the pore complex (dark orange), and the core wall (light violet). **b** Close-up views of the pore complex. Top row: central (side) view; bottom row: top view. For each view, the left panel shows annotated features, including the pore complex and surrounding A10 trimers, whereas the right panel shows the corresponding unannotated slice. **c** Subtomogram averaging (STA) map of the pore complex shown in side (left) and top (right) views. The iso-surface is colored according to the fitted model: A10 trimers (blue), outer ring (apricot), inner ring (orange), and basal ring (cappuccino). Scale bars 100 nm (**a**), 30 nm (**b**, **c**).

To assess the biological relevance of our findings, we also examined pore complexes in situ, in cells infected with VACV (see Methods for details). HeLa cells grown on EM grids were infected for 30 minutes at a multiplicity of infection of 100 before fixation and plunge-freezing. This early post-infection time point was chosen to capture incoming cores undergoing early transcription, before core uncoating and DNA release. We collected 19 tilt series of intracellular cores and processed them for STA. As the diameter of the cores exceeds the thickness of a standard cryo-FIB lamella, the cores are only partially contained within the lamellae. Milling slightly thicker lamellae, however, reduced image contrast and made visual identification of pore complexes particularly challenging (Fig. 7).

To overcome this limitation, we used the high-confidence 3D-TM with the in vitro transcription-initiated STA structure as a reference, which enabled reliable detection of pore complexes even under low-contrast conditions (Supplementary Fig. 23). In total, 159 particles were extracted for STA. Deconvolution of the tomograms allowed for visual confirmation of the template matching results, showing our predicted hexameric structure in the scarce top-views (Fig. 7a, b). Furthermore, several side-views of the pore complex could be captured, showing an expected discontinuity in the underlying core-wall (Fig. 7a, b). The resulting in situ STA reconstruction reached a resolution of 40 Å (at an FSC threshold of 0.5, as the dataset was not split into halves due to the low number of particles) and reproduced the key architectural features observed in vitro, including the six surrounding trimers and the outer, inner, and basal rings as well as the central rod (Fig. 7c). However, the limited number of particles prevented further classification, leaving open the question of whether uncapped pore complexes are also present in situ. Based on 159 particles at a resolution of ~40 Å, the in situ reconstruction supports the presence of a comparable architecture in cells but is not of sufficient resolution to validate the detailed molecular assignments or to distinguish conformational states.

## Discussion

By establishing a streamlined, high-throughput particle-picking pipeline based on deep-learning segmentation and surface meshing, we were able to selectively extract the second major building block of the palisade layer—a pore complex potentially involved in mRNA-release. Using an integrative approach that combined XL-MS, cryo-ET, STA, and AF3 modeling, we resolved this structure as a three-layered assembly composed of stacked hexamers of E8, E6, and a dodecamer of L3 forming an outer, inner, and basal ring, respectively. This architecture provided clear insight into how the complex interfaces with the surrounding A10 trimers. We found that the pore complex contacts six A10 trimers, each establishing a similar interface primarily with E6. Cross-links were consistent with interactions within the pore complex and between its components and the A10 palisade, providing support for the structural arrangement revealed by STA. Importantly, the in situ STA reconstruction closely mirrored the architecture resolved in vitro under transcription-initiated conditions, reinforcing the biological relevance of the structure. Consistent with the irregular organization of the palisade itself, the placement of pore complexes also appears random, with no detectable spatial patterning along the core surface (Supplementary Fig. 18d, e). The density corresponding to the underlying core wall is present in our averages but does not resolve, indicating that it does not follow the sixfold organization of the pore complex and its surrounding A10 trimers. The difficulty of resolving the core wall was also reported previously^6^ and is consistent with an irregular or different core-wall arrangement.

To place the structure in a functional context, we revisited previous studies on its protein components. All four proteins – E8, E6, L3, and I8—have been implicated in vaccinia virus early transcription. E8 is essential for transcriptional competence, as the temperature-sensitive E8 L81F mutant fails to synthesize early mRNA at the non-permissive temperature^17^. Similarly, a temperature-sensitive E6 P226L mutant is incapable of in vitro transcription under non-permissive conditions^21^. E6 is also required for proper assembly of MVs and without E6 expression few MVs accumulate with aberrant cores^15^. Although assembly of the brick-shaped core may be driven primarily by the core wall^6^, we speculate that E6, together with A10, might be essential for its shape and organization. L3, finally, is required for early transcription, as L3-deficient virions produce only trace amounts of early viral mRNA^16^. Our structure complements these biochemical data: the center of the complex forms a continuous hydrophilic channel (Supplementary Fig. 24), with negatively charged surfaces reminiscent of selective mRNA-translocation pores. Such features evoke conceptual parallels to the nuclear pore complex, whose electrostatic environment and modular ring architecture support nucleic-acid trafficking. The VACV pore shows similarities to the recently described coronavirus pore, formed in double-membraned ER-derived vesicles induced during infection and required for replication. This pore is also a hexameric structure composed of a membrane piercing part and a distinct cytoplasmic crown, although much larger, reminiscent of the E8 ring^22,23^. Its central channel is locally constricted, with a minimum diameter of ~2.8 nm, which would readily accommodate single-stranded RNA but may impose steric constraints on the passage of double-stranded RNA^24^. Thus, although both the nuclear- and coronavirus pores cross two lipid bilayers rather than a protein layer as for poxviruses, the organization of VACV pore is reminiscent of these assemblies and may be adapted to guide or regulate the passage of (viral) mRNA. Although direct evidence remains lacking, these structural and electrostatic properties are consistent with the idea that the pore complex may facilitate the release of newly synthesized viral mRNA from the core.

Assuming the VACV pore mediates mRNA release, its three-layered molecular architecture may provide each ring with a distinct function. The inner ring of L3 might provide a portal for the entry of core-synthesized mRNAs into the central channel. Consistently, it creates an opening towards the inside of the core by piercing through the core wall that otherwise appears as a continuous, closed layer. Interestingly, based on cross-linking data, L3 not only interacts with E6, A10 and A3 but also with two proteins required for transcription. These include D12 and I8, a transcription terminator and RNA-dependent helicase, respectively. The E6-layer in turn might provide the scaffold of the pore, held in place by its interaction with six A10 trimers. Finally, the E8 layer might provide the mRNA exit-site by facing the core outside, the cytoplasm, where translation occurs. Assuming important roles of L3 and E8 in mRNA entry into and exit from the pore, their dissociation observed under transcription conditions might reflect a change in the functional state of the pore. The uncapping we observe, as well as the breaking of the core wall during transcription, will be described in more detail in a study that is currently in preparation.

Across both untreated and transcription-initiated conditions, the central rod-like density persisted within the pore complex. By analyzing relative orientations and displacements of the rod with respect to the pore, we found that the rod is aligned with the pore axis, undergoes mostly in-plane rotational motions, and exhibits limited axial mobility. Its dynamics, together with its consistent presence under all conditions, suggest that the rod likely represents a dedicated structural element. At the same time, its overall shape is largely consistent with AF3 predictions for I8. Together with the cross-linking data and the established role of I8 in transcription, we propose that I8 is a plausible candidate for the rod-like density.

Isolated I8 displays RNA-dependent helicase activity^20^, is required for efficient early transcription in vitro, and virions lacking the protein are non-infectious^25^. Its sedimentation properties indicate that I8 is a monomer^20^, consistent with the size of the observed rod density. Extensive biochemical data led Gross and Shuman also to propose that I8 might be required for the release of mRNA from the viral core. Indeed, its location within the channel of the pore, and its persistence irrespective of whether transcription occurs, are consistent with this suggestion. Of note, the ~2 nm gap between the inner side of E6 and the rod is geometrically compatible with the passage of mRNA. The channel is predominantly electropositive, which differs from passive nucleic-acid conduits, that are typically electronegative. The poxvirus pore, however, is unlikely to be a passive conduit: mRNA export is highly ATP-dependent^4^, and if our tentative assignment of the central density to the helicase I8 is correct, export would be actively driven rather than occurring by free diffusion. Under such motor-driven translocation, transient attraction between the positively charged channel and the RNA backbone need not impede passage, as it might during passive diffusion. The precise route of mRNA relative to the central density – including whether passage requires transient axial displacement of the rod – remains to be established.

In a concurrent study, Calcraft et al. independently determined the structure of the VACV portal complex by cryo-ET^26^. The study identifies the same three components – the viral proteins E8, E6 and L3 – in the same hexameric arrangement, with E6 forming the central barrel, E8 the exterior rim and L3 an internal hexamer of dimers. The structural and molecular architecture is in close agreement with our data presented here, providing independent support for it. Overall, our study complements these data: providing additionally cross-linking restraints, systematic candidate testing, classification across transcriptional states, and a candidate assignment (I8) for the luminal density. The identification of D5 uncoating factor, located on the E8 rim, was observed on cytoplasmic cores and may explain why we did not observe it on our isolated cores. Another main difference was that Calcraft et al. did not observe a change in portal architecture upon NTP addition, whereas we observe a class lacking the E8 and L3 densities. This may reflect differences in sample preparation, NTP-incubation or classification strategies. In particular, Calcraft et al. classified particles against their previously determined portal map and retained particles contributing to well-resolved classes, whereas our classification was performed de novo, using initial references generated from random subsets of particles without relying on a pre-existing portal structure. Our approach may therefore have facilitated the detection of a broader range of portal states, including the class lacking E8 and L3.

Taken together, our work reveals a previously unknown structural module of the VACV palisade and defines its composition as stacked hexamers of E8, E6, and a dodecamer of L3 arranged in a top-to-bottom organization. The architecture, protein composition, electrostatics, and dynamic behavior of the pore complex collectively support the hypothesis that it may participate—directly or indirectly—in the release of early mRNA from the viral core. Yet several fundamental questions remain unresolved: the precise mechanistic role of the complex during transcription, the function of the central rod-like structure, and the regulation of E8/L3 dissociation during the early stages of infection. Future in situ structural and functional studies will be essential to determine whether this complex constitutes the long-sought conduit for mRNA export in a cytoplasmically replicating DNA virus.

## Methods

### Cryo-ET of VACV cores undergoing transcription in vitro

In vitro VACV cores were obtained by detergent-stripping of purified virions^7,27^. Purified VACV strain Western Reserve (VACV WR; ATCC VR-1354) at 5–10 × 10^9^ PFU/mL was incubated with an equal amount of a detergent mix (final concentration 10 mM DTT and 0.1% NP-40 dissolved in 50 mM Tris, pH 8.5) in a ThermoMixer (Eppendorf) for 30 min at 37 °C and 500 rpm. The cores were separated from the membranes by ultracentrifugation through a 36% sucrose cushion at 100,000 x *g* for 30 min using a tabletop ultracentrifuge (Beckman) and TLS55 rotor. The pellet was dissolved in 100 µl 50 mM Tris, pH 8.5. For validation and quality control, a small number of cores was applied to a nickel grid coated with formvar, negatively stained with phosphotungstic acid, and analyzed by conventional transmission electron microscopy (JEOL JEM-1400Flash, operated at 120 kV).

1.5 µL of core solution was sonicated 3 × 60 s in a waterbath sonicator and mixed with 1.5 µL of transcription mix (final concentration 1 mM GTP, 1 mM CTP, 1 mM UTP, 2.5 mM ATP, 10 mM DTT, 2 mM MgCl_2_, 10-nm gold coupled to protein A in 50 mM Tris, pH 8.5). The solution was applied to freshly glow-discharged 200-mesh R2/2 copper grids (Quantifoil), and incubated for 5 min in the blotting chamber of an EM GP2 plunge freezer (Leica Microsystems) at 37 °C and 100% relative humidity. After incubation, the grids were backside-blotted with Whatman paper 1 for 2.5 s and plunge frozen in liquid ethane at −184 °C.

Tilt series (TS) of in vitro cores were acquired using Titan Krios G2 (Thermo Scientific) operated at 300 kV and equipped with a BioQuantum-K3 imaging filter (Gatan) using SerialEM^28^. Grid squares were screened at x6500 nominal magnification and 30 µm defocus to assess target holes containing cores. TS were acquired in a dose-symmetric scheme, at ±60 in 3 degrees increments. Total dose per TS was ~133 e⁻/Å² and target defocus was set to run from −2 µm to −4.5 µm in 0.5 µm increments. Nominal magnification for TS acquisition was x64,000 resulting in a pixel size of 1.393 Å. TS were obtained with an inserted 20 eV energy slit and a 70 µm objective aperture (Table 2).

**Table 2:** Data collection parameters for all datasets

Data collection and processing	In vitro cores (transcription condition)	In vitro cores (untreated condition)		In situ cores
Microscope	Titan Krios G2	Titan Krios G4	Titan Krios G2	Titan Krios G2
Detector	K3	Falcon4	K3	K3
Energy filter	BioQuantum	SelectrisX	BioQuantum	BioQuantum
Magnification	x64,000	x105,000	x64,000	x81,000 & x53,000
Voltage (kV)	300	300	300	300
Objective aperture (µm)	70	70	70	70
Energy filter slit width (eV)	20	10	20	20
Electron exposure per tilt (e⁻/Å²)	~3.3	~3	~3.3	~3.1
Total electron dose (e⁻/Å²)	~133	~125	~133	~120
Dose rate (e⁻/pixel/sec)	~6	~6	~6	~6
Fractions per tilt	10	10	10	10
Defocus range (µm)	-2 to -4.5	−1.5 to −4.5	–2 to -4.5	–3 to –4.5
Tilt range (degrees)	±60	±60	±60	±57
Tilt step (degrees)	3	3	3	3
Acquisition scheme	Dose-symmetric	Dose-symmetric	Dose-symmetric	Dose-symmetric
Tilt groups	2	2	2	2
Pixel size (Å)	1.393	1.223	1.393	1.112 & 1.697
Associated EMPIAR ID	EMPIAR-13805	EMPIAR-13803		EMPIAR-13806
Associated EMDs	EMD-57290	EMD-57291		EMD-58349
	EMD-57292	EMD-57293		
	EMD-57294	EMD-57295		
	EMD-58326	EMD-58328		
	EMD-58327			

For information on STA processing of the listed maps see Supplementary Tables 11,12,14, and 15.: Data collection parameters for all datasets. For information on STA processing of the listed maps see Supplementary Tables 11,12,14, and 15.

In total, 89 newly acquired tilt series (TS) were processed as previously described^7^. Briefly, tilt series were dose-filtered using cryoCAT, and defocus parameters were estimated with CTFFIND4^29^. Corrupted images within tilt series were manually removed prior to fiducial-based alignment performed in eTomo (IMOD)^30^. Two sets of tomograms were reconstructed: one using novaCTF^31^ with 3D-CTF correction for downstream STA, and a second using weighted back projection (WBP) in eTomo with a SIRT-like filter for visualization.

### Cryo-ET of VACV in vitro cores in the untreated condition

To analyze the untreated condition, a previously published dataset (EMPIAR-11674) comprising 38 tilt series, acquired on a Titan Krios G4 with a Falcon4 detector and SelectrisX energy filter at a pixel size of 1.56 Å (Liu et al., 2024), was extended with additional data acquired under otherwise identical conditions on two microscopes: a Titan Krios G4 (Falcon4 detector, SelectrisX energy filter) at a nominal magnification of x105,000 (pixel size 1.223 Å), and a Titan Krios G2 (K3 detector, BioQuantum energy filter) at a nominal magnification of x64,000 (pixel size 1.393 Å). All parameters are given in Table 2. The newly acquired tilt series were processed as described above, and for STA all tomograms were rescaled using cryoCAT to the pixel size of the original dataset (1.56 Å). This resulted in a combined set of 100 tomograms for the untreated condition, alongside 89 tomograms obtained for the in vitro transcription-initiated condition.

### Cryo-ET of VACV cores undergoing transcription in situ

HeLa cells (DSMZ, ACC 57) were obtained from the DSMZ collection. The cell line was not authenticated in-house and tested negative for mycoplasma contamination. For in situ cryo-ET of VACV cores, the cells were seeded onto 200-mesh R1.2/20 SiO₂/Au grids (Quantifoil). The grids were constrained to an area of 30 mm² using FLEXIPERM silicon gaskets (Sarstedt), matching the area of a standard 96-well plate, to improve cell adhesion to the grids. Prior to seeding, grids were glow discharged, laminin coated (Mouse Laminin, Sigma-Aldrich), and UV-sterilized. Per grid 1.5 × 10^4^ cells were seeded, attached for 3–4 h and then incubated overnight in 200 µL fresh DMEM (Gibco) supplemented with 10% fetal calf serum (FCS).

After incubation, the medium was changed to DMEM w/o FCS, but with 20 mM HEPES to stabilize pH and facilitate infection. The cells were then infected with multiplicity of infection (MOI) 100 of VACV WR (optionally with 5 µg/mL actinomycin D). To synchronize infection, the cells with added virus were kept at RT for 15 min, and mixed by pipetting every 5 min. Infection then took place at 37 °C and 5% CO_2_ for 30 min, before fixation by adding a 2x fixative solution (0.2 M PHEM buffer with 8% PFA) dropwise to the medium.

The grids with infected and fixed cells were then plunge frozen in a GP2 plunge freezer (Leica). 3.5 µL of PHEM buffer was added to the grid in the blotting chamber from the backside and the grid was instantly back-blotted for 8 s on Whatman paper 1 before freezing in liquid ethane at −184 °C. Temperature in the blotting chamber was set to 8 °C and humidity to 80%.

Cryo-lamellae were prepared using an Aquilos2 Cryo-FIB-SEM (Thermo Scientific) using Maps and AutoTEM software (Thermo Scientific). Target thickness was set to 160 nm, and milling angle to 10 degrees. Eucentricity and positioning steps were prepared manually for each grid; milling and polishing were performed automatically overnight. An additional manual polishing step with 0.5 degrees overtilt was performed where necessary. Per session approximately 20 lamellae were obtained. Initial samples were prepared using VACV A3-YFP, a virus strain with fluorescence-labeled cores, and Hoechst 33342 staining, to assess core distribution in the cells. For that, the built-in cryo-fluorescence microscope of Aquilos2 was used. Once the results were satisfactory, wild-type virus without additional staining was used. The overview of this workflow is depicted in Supplementary Fig. 25.

TS of the lamellae were acquired using a Titan Krios G2 (Thermo Scientific) operated at 300 kV and equipped with a BioQuantum-K3 imaging filter (Gatan). Lamellae were screened at x11,500 nominal magnification and 70 µm defocus with a higher-than-usual dose to be able to locate VACV cores in the cell using SerialEM. TS were acquired in a dose-symmetric scheme, pre-tilted to the milling angle and acquired at ±57 degrees in 3 degrees increments. Total dose per TS was ~120 e⁻/Å² and target defocus was set to run from −3 µm to −4.5 µm in 0.5 µm increments. Nominal magnification for TS acquisition was x53,000 resulting in a pixel size of 1.697 Å and x81,000 resulting in a pixel size of 1.112 Å. TS were obtained with an inserted 20 eV energy slit and a 70 µm objective aperture (Table 2).

Frames of TS were motion-corrected on-the-fly during acquisition using SerialEM. CTF estimation of the resulting stacks was performed with Gctf^32^ for in situ TS. Dose filtering was performed using cryoCAT, followed by manual cleaning of the stacks. For alignment and reconstruction, eTomo was used, using patch-tracking alignment for in situ TS. Reconstruction was performed using a SIRT-like filter equivalent to 15 iterations in eTomo for segmentation and visualization, and 3D-CTF-corrected reconstruction using NovaCTF for STA. Tomograms shown in Fig. 7 were deconvolved using cryoCAT.

### Training of the 3D U-Net segmentation model

In total, 14 tomograms reconstructed with SIRT-like filter at binning 8 from IMOD were used for training. These tomograms contained VACV cores from both untreated and transcription-initiated conditions. They covered three pixel sizes (1.56 Å, 1.223 Å, and 1.393 Å) and three image dimensions (464 by 464, 512 by 512, and 512 by 720 pixels). In addition, four tomograms were randomly selected for validation, ensuring that at least one tomogram from each condition was included. The diversity in imaging and sample conditions reduces instrument-specific bias and improves model robustness.

To generate high-quality ground truth data for network training, a model-assisted iterative labeling strategy was employed. In the first iteration, a 3D U-Net^33^ was trained using weak labels generated from seven tomograms of in vitro membrane-free cores, each with a pixel size of 1.56 Å. The boundaries of the VACV cores were manually annotated as layers in Napari^34^, and then processed into segmentations using cryoCAT^13^. The model with the lowest validation loss was selected as the best-performing network. This trained model was then applied to the same tomograms and five additional tomograms with a pixel size of 1.223 Å. The prediction outputs were thresholded at around 0.4, and the largest connected components were extracted to form segmented volumes. These volumes were further refined and post-processed using cryoCAT, with manual corrections performed in Dragonfly^35^. In the second iteration, the 3D U-Net was retrained using the manually corrected segmentations generated from the previous iteration. The updated model was then applied to predict masks for all training and validation tomograms, including the in vitro transcription-initiated cores with a pixel size of 1.393 Å. All predicted masks were visually inspected and manually refined in Dragonfly to ensure segmentation accuracy. These finalized masks were subsequently used to train the network for automatic segmentation of the complete tomogram dataset^36^. Comparison of predictions on the validation data between the first- and final-iteration models revealed a clear improvement in network performance (Fig. 2).

Model training was carried out on a workstation equipped with two NVIDIA GeForce RTX 3090 GPUs (24 GB memory each). During training, each training tomogram and its mask were augmented with horizontal flipping and rotation of 90°,180° and 270° to increase the size of training dataset. Model performance was evaluated on four independent validation tomograms. The segmentation achieved a Dice similarity coefficient (DSC) of 0.732 ± 0.123 and a Jaccard index (IoU) of 0.588 ± 0.144. Pixel-wise evaluation yielded a precision of 0.914 ± 0.026 and a recall of 0.628 ± 0.166, indicating high prediction accuracy with moderate sensitivity.

The final prediction outputs were postprocessed using classical morphological operations to bridge small gaps between segmented objects. Challenging cases were manually corrected in Dragonfly. A segmentation mask was produced for every tomogram.

### Initial reference generation

To generate an initial template, 32 pore complexes were manually identified from a previously published dataset of 34 tomograms of the in vitro untreated condition (EMPIAR-11674) and averaged at bin 8 using novaSTA^37^; because pore complexes are difficult to recognize in central views, only top-view pores were picked. Subtomogram averaging of these particles without imposed symmetry (C1) already revealed a hexameric arrangement, and a C6-symmetrized average of the same particles showed the six-fold feature at higher contrast. This average was rescaled to bin 4 and used as the template for template matching. Full parameters for each step are given in Supplementary Table 9.

### Three-dimensional template matching

A high-confidence 3D template-matching (3D-TM) pipeline based on GAPSTOP^TM^ was implemented to identify pore complexes within tomograms. The rescaled reference (see above) was used for a first round of 3D-TM on seven tomograms, performed without imposed symmetry (C1), together with the corresponding wedge list and filter definitions. TM produced cross-correlation (CC-score) volumes in which high-scoring peaks corresponded to candidate pore positions; peaks were isolated per tomogram using manually defined thresholds, compiled into particle lists, and curated manually in ArtiaX^38^, yielding 157 high-confidence particles. These were aligned with C6 symmetry over four iterations at bin 4 to generate an improved reference, which was then used for a final round of 3D-TM, again without imposed symmetry (C1), across all tomograms of both conditions. The TM output was masked using segmentations generated by the self-trained 3D U-Net model to produce the candidate particle lists. Symmetry and all other parameters for each step are provided in Supplementary Table 9. One example result on the in vitro transcription data is shown in Fig. 2c, where the CC-score map displays sharp, well-isolated, high-contrast peaks relative to the background.

### Particle cleaning

A surface mesh describing the geometry of the VACV core surface was generated from each segmentation volume using the marching cubes algorithm implemented in scikit-image^39^, with a step size of 5 to reduce computational cost (Fig. 2). A point cloud was then generated by oversampling this mesh, with each point carrying both its spatial coordinates and its local surface orientation (normal). Points were classified as inward- or outward-facing by comparing each surface normal with the vector pointing toward the core center, together with the point’s position relative to the center. The outward-facing surface points and their normals were used to evaluate the particles identified by template matching: particles whose orientations differed by more than 90° from the nearest outward surface point were excluded from further processing.

### Pore complex symmetry validation

The six-fold symmetry of the pore, initially apparent by visual inspection of the reference and reconstructions, was validated by an in-plane angle randomization test to confirm it originates from the data rather than from the C6-averaged reference. The particle lists from the final TM and geometry cleaning (2,678 and 2,590 particles for the transcription and untreated conditions) were used directly, with no intervening symmetrized alignment. The in-plane angle of every particle was randomized, producing a featureless, tube-like average without six-fold features. Starting from these randomized orientations, STA was performed with C1 symmetry using a large cylindrical mask that partially included the surrounding A10 trimers, and subsequently with a tight mask focused on the pore alone (Supplementary Table 10, Supplementary Fig. 3). In both conditions and with both masks, the six-fold arrangement was recovered across all three protein layers, in an orientation independent of the template-matching reference; the pore-to-wall connection at the level of E6 was likewise recovered independently in the two conditions in different absolute orientations. As the surrounding A10 trimers contribute to any rotational or Fourier-based symmetry metric, symmetry was assessed by direct visual inspection of the pore density.

### Subtomogram averaging and classification of the pore complex

In total, 2,678 pore complexes were identified under in vitro transcription conditions, and 2,590 were identified in untreated cores. STA was performed in STOPGAP^40^ with C6 symmetry (validated as described above). Alignment was performed using a cylindrical mask that included the surrounding A10 trimers, followed by a tighter mask excluding A10 and encompassing only the pore complex, and finally focusing on the asymmetric unit. A full list of parameters is provided in Supplementary Tables 11 and 12.

For classification, five de novo initial references were generated from random subsets of 300 particles each. Classification was performed in STOPGAP separately for each condition, with each run consisting of 29 iterations. Simulated annealing was applied during the first 10 iterations, with the temperature factor decreasing from 10 to 1, followed by a random search strategy for the remaining iterations^41^. Convergence of the classification was observed for both conditions (Supplementary Fig. 26), and the full set of classification parameters is provided in Supplementary Table 13.

### Subtomogram averaging and classification of the rod density

To align and classify the central rod independently of the pore, focused classification was performed on the rod density in STOPGAP. A focused mask enclosing only the central rod and excluding the surrounding pore rings (E8, E6 and L3) was used, so that alignment was driven by the rod rather than the pore walls. C1 symmetry was applied throughout, as the rod does not share the six-fold symmetry of the pore. The rod-focused alignment was performed independently of the full-pore alignment, and the two sets of particle orientations were compared to separate rod movement from pore movement. Five de novo initial references were generated from random subsets of 300 particles each, and classification was run separately for each condition, with 24 iterations per run: simulated annealing during the first 10 iterations (decreasing from 10 to 1), followed by a random search strategy for the remainder^41^. In both transcribing and untreated conditions, all classes converged to a single class without improvement in the density, which remained featureless. Full parameters, including mask dimensions, are given in Supplementary Table 13.

### Subtomogram averaging and classification of A10 trimer

For the transcribing condition, A10 trimers were identified by oversampling-based particle picking as described above and in ref. ^7^, followed by cleaning to remove false positives and subsequently also misaligned particles. The final number of particles was 168,352, yielding a reconstruction at a pixel size of 2.786 Å and a final resolution of 7.7 Å. For the untreated condition, STA was not repeated, as the A10 trimer structure had been resolved previously^6,7^. Full set of STA parameters is provided in Supplementary Table 14.

To analyze A10 trimers surrounding the pore complex, particle positions and orientations were defined relative to the pore coordinates, and a new particle list was generated accordingly. STA was performed for both transcribing and untreated conditions using this particle set (for 16,068 and 15,540 particles, respectively) with five iterations of alignment. However, no improvement in the reconstruction was observed in either case. Therefore, the reported structures correspond to averages obtained directly from the newly generated particle lists without further alignment.

### Subtomogram averaging of the in situ pore complex

Particles were picked as described above from tomograms with a pixel size of 6.788 Å. In total, 159 particles were identified and used for STA. Due to the limited number of particles, STA was performed at a binned pixel size of 13.576 Å. The dataset was not split into independent halves, and the reported final resolution of 40 Å corresponds to an FSC threshold of 0.5. Full set of STA parameters is provided in Supplementary Table 15.

### AlphaFold3 and AF3x prediction and systematic fitting

AlphaFold3 (AF3; version 3.0.1, run as a local installation) was used to generate hexameric models of E8, E6, and L3. In addition, cross-linked residue pairs from XL-MS were incorporated into AF3x (version 0.0.0.20250316, run as a local installation), a cross-link-restrained implementation, restricting the input to cross-links with inter-residue distances below 30 Å.

For model fitting, the pore complex density was Gaussian-filtered (σ = 2) in UCSF ChimeraX to reduce high-frequency noise. The density map was then masked to separately isolate the outer, inner, and basal ring regions. AF3-predicted hexameric models of E8 and L3 were fitted into the corresponding masked densities following the procedure described in Zimmerli et al^42^. For L3, the predicted flexible N-terminal region (Met1–Gly65) was removed prior to fitting. Notably, attempts to predict the L3 dodecamer using both AF3 and AF3x did not yield plausible assemblies. Therefore, the final L3 model was generated by fitting a single protomer from the AF3 hexamer prediction into the density map and expanding it using C6 symmetry in UCSF ChimeraX to reconstruct the full dodecamer. All models were filtered to 8 Å resolution, and corresponding simulated density maps were generated. These maps were systematically fitted into the STA density using UCSF ChimeraX through the Assembline framework. Fitting was performed using 10,000 random initial placements, requiring at least 30% overlap between the model-derived map and the experimental density.

Because the AF3-predicted E6 hexamer adopted a conformation that differed from the pore complex density, E6 was treated differently from E8 and L3 during model fitting. Inspection of the E6 prediction revealed two flexible boundaries, at Lys163-Lys164 and Asn409-Glu410, delimiting three segments along the sequence, consistent with the three blocks apparent in the predicted aligned error (PAE) plots (Supplementary Fig. 12c). As the N- and C-terminal segments together form the lower part of the hexamer while the intervening middle segment extends beyond the density, the model was divided into two rigid bodies rather than three: a lower domain comprising the N- and C-terminal segments (Met1–Lys163 and Glu410–Ser567), which fit the density as predicted, and an upper domain comprising the middle segment (Lys164–Asn409), which overflows the density in the AF3 prediction. Among the AF3x predictions of the pore complex assembly, only one model exhibited a closed E6 hexamer (Supplementary Fig. 12a), ranking second among the predictions. The upper domain of E6 from this AF3x model was therefore used to represent the overflowing middle segment of the E6 hexamer. Accordingly, the lower domain of the AF3 E6 hexamer model and the upper domain extracted from the AF3x pore complex model were systematically fitted into the inner ring density of the pore complex (Fig. 3e, Supplementary Fig. 12d).

To ensure a physically plausible assembly, an additional global optimization step was performed using Assembline. The upper (AF3x-derived) and lower (AF3-derived) domains of the E6 hexamer were defined as separate rigid bodies connected by flexible linkers at both segment boundaries (Lys164–Asp166 and Ile407–Thr412). These linkers were represented as single-residue beads with a connectivity restraint weight of 50, allowing limited flexibility while maintaining continuity between the two domains.

### Fitting of candidate models into the pore density

Candidate proteins were predicted with AF3 across oligomeric states from monomer to dodecamer (see above). Each prediction was rigid-body fitted into the STA reconstruction of the pore complex with the fitmap command in UCSF ChimeraX. Prior to fitting, the central rod-like density was masked out and the map was contoured at a permissive level (0.35) so that placement was not biased toward any subregion. For each model a global search was performed from 200 random initial placements (fitmap with resolution 8, metric correlation, search 200), and the best-scoring solution was retained. Fit was quantified by the cross-correlation and the correlation about the mean between the map and a model density simulated at 8 Å on the map grid.

To distinguish models contained within the pore density from those exceeding it, we additionally computed an overflow measure. Each fitted model was converted to a density at 8 Å on the map grid (molmap), both maps were thresholded at the fitting level (0.35), and overflow was defined as the fraction of the model volume lying outside the pore density. A well-accommodated subunit lies largely within the density (low overflow), whereas a model exceeding the dimensions of the density gives high overflow.

### L3 basal assembly

Predictions of L3 were generated with AlphaFold3 and AF3x as monomers and as oligomers up to the dodecamer. As higher-order oligomers did not converge on a consistent dodecameric arrangement (yielding overlapping hexamers or approximately D6-symmetric stacked hexamers), the basal assembly was modeled in a density-guided manner in UCSF ChimeraX. A single L3 protomer was rigid-body fitted into an isolated lobe of the basal density using fitmap (global search, map simulated from the model at 8 Å resolution), and the fitted protomer was expanded under Cn symmetry (*n* = 1-12) about the pore axis using the sym command. Each expansion was fitted to the basal density and scored by cross-correlation (CC) and correlation about the mean (CAM); we note that CAM decreases with increasing subunit number as the model samples progressively weaker peripheral density, and CC was therefore used as the primary metric for comparing symmetries. Compatibility of each expansion with the density (absence of overflow) was assessed with measure mapvalues, comparing the mean map value sampled at atom positions for each expansion with that of the single fitted protomer. As an independent test, six L3 protomers were fitted individually into the six lobes of each ring and scored as above. Inner and outer rings of the basal assembly were treated separately. Contour level 0.35 was used throughout for display and masking; the same fitting protocol and map resolution were applied to all comparisons. Cross-links involving L3 were mapped onto the resulting model and inspected for Cα–Cα distance compatibility ≤30–35 Å for DSSO.

### AlphaFold3 seed scan of the E6 hexamer

To assess how reproducibly AF3 predicts the E6 hexamer and whether it recovers the closed conformation seen in the density, 500 predictions of the E6 homohexamer were generated across independent seeds and evaluated for symmetry and for conformation separately, as a symmetric ring can be either open or closed.

Symmetry was determined from the rigid-body transform relating each protomer to its neighbor. For every model, protomers were ordered around the ring and the transform mapping the ring onto its one-step-rotated copy was obtained by Kabsch superposition; its rotation angle gives the rotation per subunit (60° for an ideal hexamer), the consistency of its rotation axis across protomer pairs measures how regular the ring is (1.0 for a perfect ring), and the translation along the axis (screw rise) distinguishes a flat ring ( ≈ 0) from a spiral. A small superposition residual (RMSD between adjacent protomers) confirms the protomers are equivalent.

Conformation – that is, whether the ring is closed as in the density or splayed open – was quantified in two ways. First, each model was compared to the fitted E6 model by a six-fold-aware Cα RMSD: the two rings were superposed testing every cyclic protomer registration and both ring directions, and the lowest RMSD retained, so that a high value reflects a genuinely different conformation rather than a mismatched protomer numbering. Second, as a reference-free measure of opening, the ring radius (mean protomer distance from the symmetry axis) and axial span were computed directly from each model; an open ring expands radially and elongates along the axis. Models were grouped by these conformational descriptors using agglomerative (Ward) clustering, and the representative (medoid) of each group was inspected in ChimeraX. Adjacent-protomer interface confidence was read from the AF3 chain-pair ipTM and PAE for neighboring protomers.

All 500 predictions formed regular six-fold rings (rotation per subunit 60.0 ± 0.0°, axis consistency 0.998, screw rise ≈ 0, adjacent-protomer superposition RMSD ≈ 2 Å). None matched the closed conformation of the fitted model: the six-fold-aware Cα RMSD was 13.8 Å at best and 24.7 Å at the median, and the ring radius (51–65 Å) was consistently larger than in the fitted model, i.e. every prediction was more open. Analysis used a custom Python script (gemmi, NumPy, scikit-learn), available at Zenodo^19^.

### Evolutionary conservation analysis

Per-residue evolutionary conservation was calculated for E6, E8 and L3 using the ConSurf web server^43^. Homologs were collected from the UniRef90 database with HMMER (one iteration, E-value cutoff 1 × 10⁻⁴), and filtered to a maximum sequence identity of 95% (CD-HIT), a minimum sequence identity to the query of 35%, a minimum query coverage of 60%, and a maximum overlap between homologs of 10%. Multiple sequence alignments were built with MAFFT, phylogenetic trees were constructed by neighbor joining with maximum-likelihood distances, and conservation scores were calculated using the Bayesian method and mapped onto a 1–9 grade scale (9 = most conserved). The amino-acid substitution model was selected for each protein with ProtTest 3.4.2^44^ according to the AICc criterion.

For E6, 32 homologs were retrieved and the calculation was performed on 28 unique sequences (query included; average pairwise distance 0.71, range 0.13–1.25) using the LG substitution model. For E8, 24 homologs were retrieved and the calculation was performed on 24 unique sequences (average pairwise distance 0.62, range 0.10–1.10) using the WAG model. For L3, 37 homologs were retrieved and the calculation was performed on 28 unique sequences (average pairwise distance 0.90, range 0.07–1.47) using the LG model. Conservation scores were computed on the individual monomers, independently of the structural model, the density, or the oligomeric state, and were subsequently mapped onto the fitted model for analysis of the inter-protomer interfaces.

### Sensitivity of the predicted L3 assembly to targeted substitutions

To test in silico whether the three-stranded β-sheet formed between neighboring protomers of the inner L3 ring is required for the predicted hexameric arrangement, four hexameric L3 assemblies were predicted with AlphaFold3: the wild-type sequence and three substitution variants. In the first variant, residues 322–329 and 338–341 – the two strands that form the inter-protomer sheet – were replaced by proline, whose backbone lacks the amide hydrogen required for β-sheet hydrogen bonding and whose ring restricts backbone conformation. Two controls were included to distinguish the effect of removing β-sheet compatibility from the effect of substituting twelve residues. In the first control, the same twelve positions were replaced by valine, a β-branched residue with high β-sheet propensity. In the second, twelve prolines were introduced into two surface loops remote from any inter-protomer interface (residues 137–140 and 147–154), matching the strand substitution in both the number of substitutions and their division into two segments. Sequences of all four constructs are available at Zenodo^19^, together with the corresponding predictions. Predicted models were analyzed as output, without energy minimization or other refinement.

All comparisons were performed in UCSF ChimeraX using Cα atoms only. Residues 1–65 were excluded from every superposition and from every reported value, as they are predicted to be disordered and are not resolved in the subtomogram average; the analyzed region is therefore residues 66–350. Only residues present in all six chains of all four models were used.

Protomers were compared individually. Each protomer of each variant was superposed onto the corresponding wild-type protomer using body residues only—residues 66–350 excluding the substituted strand region (315–345) and the substituted loops (137–140 and 147–154)—so that the substituted regions did not contribute to the reference frame. Per-residue Cα deviations were calculated in this frame across the whole analyzed region, including the excluded regions, and averaged over the six protomers. Root-mean-square deviations were calculated separately for the substituted strands (322–329, 338–341), the surrounding strand region (315–345), the substituted loops, and the remainder of the protomer, and are reported as mean ± s.d. over the six protomers. Regional values spanning the N-terminal and C-terminal parts of the protomer were calculated from the mean per-residue deviation profile rather than per protomer.

The arrangement of protomers was assessed separately. All six protomers of each variant were superposed simultaneously onto the wild-type hexamer using the same body residues, by Kabsch superposition of the concatenated Cα coordinates. Chain identifiers are assigned arbitrarily by the prediction and did not follow the same order around the ring in the four models, so protomers were paired by their position in the ring rather than by chain identifier. The ring order of each model was determined from inter-protomer contacts, and all twelve pairings consistent with a closed ring of six protomers (six rotational offsets x two directions) were evaluated; the lowest root-mean-square deviation obtained is reported, together with the pairing that produced it (Supplementary Table 7). This value measures the overall deviation of the assembly and does not distinguish between differences in protomer conformation, in protomer orientation within the ring, and in ring dimensions; it is therefore reported alongside the per-protomer values above rather than in place of them.

Buried surface area and hydrogen bonds between adjacent protomers were measured for each model with the measure *buriedarea* and *hbonds* commands in ChimeraX using default parameters, for the protomer pair identified as adjacent in that model. Per-protomer, per-residue and ring-pairing values for all four models are provided as Source Data. Analysis scripts are available at Zenodo^19^.

### Crosslinking mass spectrometry

VACV cores were prepared as described earlier. The core pellet was washed 5 times in PBS or 20 mM HEPES by centrifugation at 100,000 x *g* for 30 min followed by resuspension in the respective buffer. Disuccinimidyl sulfoxide (DSSO) was dissolved freshly in water-free DMSO and added to the cores with a final concentration of 3 mM. Following a 30 min incubation at 20 °C and 1000 rpm in a ThermoMixer (Eppendorf), the reaction was stopped by adding TRIS pH 8 buffer to a final concentration of 30 mM followed by 30 min incubation at 20 °C and 1000 rpm.

The core preparations were supplemented with urea to a final concentration of 8 M and lysed by one minute of intermittent treatment with a sonication probe tip (30 % amplitude). 250 units of Benzonase® Nuclease (Merck) were added to each sample and incubated for 2 h at 25 °C. Then, Tris(2-carboxyethyl)phosphine hydrochloride (TCEP) and chloroacetamide (CAA) were added to final concentrations of 5 mM and 40 mM, respectively. Samples were incubated for 1 h at 30 °C while shaking to reduce and alkylate proteins. Subsequently, Lys-C (1:75 (*w/w*)) was added and incubated for 2–4 h at 30 °C. After dilution to 2 M urea with 50 mM triethylammonium bicarbonate buffer pH 8.0, 1:100 (*w/w*) of Trypsin was added and samples were digested overnight while shaking. Formic acid (FA) was added to a final concentration of 1 % to stop the proteolytic digestion. Peptides were desalted using 1 mL Sep-Pak® C8 cartridges (Waters) according to the manufacturer’s protocol.

### SCX fractionation

Peptides were resuspended in 0.05 % FA and 20 % ACN in water (SCX solvent A) and fractionated using strong cation exchange chromatography (SCX) using an Agilent 1260 Infinity II HPLC system and a PolySULFOETHYL A 100 × 2.1 mm column (PolyLC). 100 s fractions were collected during a 95 min gradient from 0 % SCX solvent A to 80 % SCX solvent B (500 mM NaCl, 20 % ACN, 0.05 % FA). Fractions were desalted using C8 stage tips and dried in a vacuum concentrator. Dried samples were stored at −20 °C before resuspension in 1 % ACN, 0.05 % TFA for LC-MS data acquisition.

### LC-MS settings for XL-MS

XL-MS data was acquired on an Orbitrap Fusion Lumos Tribrid Mass Spectrometer (Thermo Fisher Scientific) equipped with a FAIMS Pro Duo interface (Thermo Fisher Scientific) and on-line separation using an Ultimate 3000 RSLC nano LC system (Thermo Fisher Scientific) and a 50 cm in-house packed (Poroshell 120 EC-C18, 2.7 µm, Agilent Technologies) reversed-phase analytical column. Approximately 1 µg of peptides were loaded and separated during a 180 min gradient from 0.1 % FA in water to 0.1 % FA in 80 % (v/v) ACN at 250 nL/min. FAIMS compensation voltages were altered between −50, −60 and −75 V.

Cross-linked peptides were detected using a MS2 acquisition scheme with a stepped HCD fragmentation method. MS1 scans were acquired in a range from 375 to 1600 m/z and at a MS1 resolution of 120,000. Maximum injection time was set to 50 ms. Precursor charge states 4–8 were selected and fragmented using HCD collision energies of 21 %, 27 % and 33 %. MS2 spectra were acquired with a 1.6 m/z isolation window and at a resolution of 60,000. Dynamic exclusion was set to 60 s.

### XL-MS data analysis

Cross-linking database search was performed using Scout V2. MS1 and MS2 mass tolerances were set to 10 ppm and 20 ppm, respectively. Peptides with a length of 7 to 60 amino acids were considered. 3 missed cleavages and 2 variable modifications were accepted. Fixed modification: cysteine carbamidomethylation. Variable modification: methionine oxidation. Enzymatic digest was set to Trypsin (fully specific) and the cross-linker was set to DSSO (158.0038 Da, short arm 54.0106 Da, long arm 85.98234 Da) reacting with lysine, serine, tyrosine and threonine as well as N-termini. A concatenated database consisting of the VACV proteome (Uniprot Proteome ID UP000000344) and human database of host cell proteins as identified from a bottom-up proteomic run was used.

The results were filtered consecutively to 1 % FDR on cross-link spectrum match, residue-pair and protein-protein interaction level using the conventional FDR option in Scout V2.

To plot cross-links onto the proposed structure, a custom R script and the bio3d package were used. For this, cross-links were filtered to 1 % FDR consecutively on crosslink-spectrum-match and residue pair level. Cross-Links were visualized by PyMOL^45^. Networks were generated using Cytoscape^46^ with the XlinkCyNET plugin^47^.

### Spatial analysis of the pore complex

Spatial and orientational relationships between cap ring complexes and central rods were analyzed identically for both untreated and transcription conditions. Analysis was performed using the TANGO module^48^ with in cryoCAT, with ring complexes defined as query particles and rods as nearest neighbors. For the latter, the particle list from the classification as described above was used as input. For each ring–rod pair, relative orientations were extracted and visualized using rectangular polar histograms, showing the distribution of rotation angles (Supplementary Fig. 21). Shifts along the canonical coordinate axes were extracted from the TANGO outputs. Particles were then grouped based on axial (z) displacements, and subtomogram averages were generated from the original query particles using three bins: shifts ≤−2 nm, shifts within ±0.5 nm, and shifts ≥+2 nm (Supplementary Fig. 21b). To assess whether axial displacements correspond to specific structural states, translation data were combined with classification results; however, no enrichment of particular classes within defined shift ranges was observed (Supplementary Fig. 21c).

### Reporting summary

Further information on research design is available in the Nature Portfolio Reporting Summary linked to this article.

## Supplementary information

Supplementary information

Description of additional supplementary files

Supplementary video 1

Reporting Summary

Transparent Peer Review file

## Source data

Source data

## Data Availability

The electron microscopy density maps generated in this study have been deposited in the Electron Microscopy Data Bank (EMDB) under accession codes EMD-57290 (pore complex with surrounding A10 trimers from in vitro cores, transcription condition), EMD-57291 (pore complex with surrounding A10 trimers from in vitro cores, untreated condition), EMD-57292 (pore complex, transcription condition), EMD-57293 (pore complex, untreated condition), EMD-57294 (single asymmetric subunit of the pore complex from in vitro cores, transcription condition), EMD-57295 (single asymmetric subunit of the pore complex from in vitro cores, untreated condition), EMD-58326 (A10 trimer from in vitro cores, transcription condition), EMD-58327 (A10 trimers contacting the outer surface of the vaccinia virus pore complex, transcription condition), EMD-58328 (A10 trimers contacting the outer surface of the vaccinia virus pore complex, untreated condition), and EMD-58349 (pore complex from in situ cores).The raw tilt series generated in this study have been deposited in the Electron Microscopy Public Image Archive (EMPIAR) under accession codes EMPIAR-13805 (transcription condition), EMPIAR-13803 (untreated condition) and EMPIAR-13806 (in situ tilt series). The previously published raw tilt series used in this study are available in EMPIAR under accession code EMPIAR-11674 (untreated condition). The mass spectrometry proteomics data generated in this study have been deposited in the ProteomeXchange Consortium via the PRIDE partner repository under accession code PXD076400. The AlphaFold3 and AF3x predicted models, additional predictions, analysis scripts and derived data generated in this study—including the oligomeric-state sweeps, the E6 hexamer seed scan, the rod candidate models, the L3 substitution constructs, and the conservation analyses – have been deposited at Zenodo^19^ under accession code [https://doi.org/10.5281/zenodo.21479484]. The previously published structural model used in this study is available in the Protein Data Bank under accession code 8P4K. Source data are provided with this paper.

## Code availability

All code required to reproduce the analyses presented in this study is publicly available within the cryoCAT [https://github.com/turonova/cryoCAT] toolbox or at Zenodo under accession code [https://doi.org/10.5281/zenodo.17817056]. The analysis scripts specific to this study are available at Zenodo [https://doi.org/10.5281/zenodo.21479484]. The model used for 3D U-Net–based core picking as well as a tutorial for the training and segmentation are publicly available on Zenodo under accession code [https://doi.org/10.5281/zenodo.20262954].
