# Supplementary material for: A multimeric pore complex in the poxvirus core revealed by integrative structural analysis
Source: Nat Commun. 2026 Oct 30;17:11292. doi: 10.1038/s41467-026-79013-7 (PMC13722698; doi:10.1038/s41467-026-79013-7)
Supplement: Supplementary file 1 [file 41467_2026_79013_MOESM1_ESM.pdf]

SUPPLEMENTARY INFORMATION

A multimeric pore complex in the poxvirus core revealed by integrative structural analysis

Jiasui Liu[1,†], Simon Schäfer[2,3,†], Svenja Lehnung[2,3,†], Lukas Baumann[4,5], Lars Mühlberg[4], Cornelia Steiner[2], Markus Schreiber[1,6], Mark Linder[7], Sonja Welsch[7], Fan Liu[4,8], Boris Bogdanow[4,5,*], Jacomina Krijnse Locker[2,3, *], Beata Turoňová[1,9*]

[1]Department of Molecular Sociology, Max Planck Institute of Biophysics, Frankfurt am Main, Germany

[2]Division of immunology, Paul Ehrlich Institute, Langen, Germany

[3]Microscopy of Pathogens, Paul Ehrlich Institute, Langen, Germany

[4] Department of Structural Biology, Leibniz-Forschungsinstitut für Molekulare Pharmakologie (FMP), Robert-Roessle-Str. 10, 13125 Berlin, Germany

[5] Charité - Universitätsmedizin Berlin, corporate member of Freie Universität Berlin and Humboldt-Universität zu Berlin, Institut für Virologie, Berlin, Germany

[6] IMPRS on Cellular Biophysics, Frankfurt am Main, Germany

[7] Central Electron Microscopy Facility, Max Planck Institute of Biophysics, Frankfurt am Main, Germany

[8] Charité Universitätsmedizin Berlin, Berlin, Germany

[9] Cluster of Excellence SubCellular Architecture of Life (SCALE), Goethe University Frankfurt, Frankfurt, Germany

† Authors with equal contributions

*** Corresponding authors** (BB: boris.bogdanow@charite.de, JKL: Jacomina.KrijnseLocker@pei.de, BT: beata.turonova@biophys.mpg.de)

SUPPLEMENTARY FIGURES

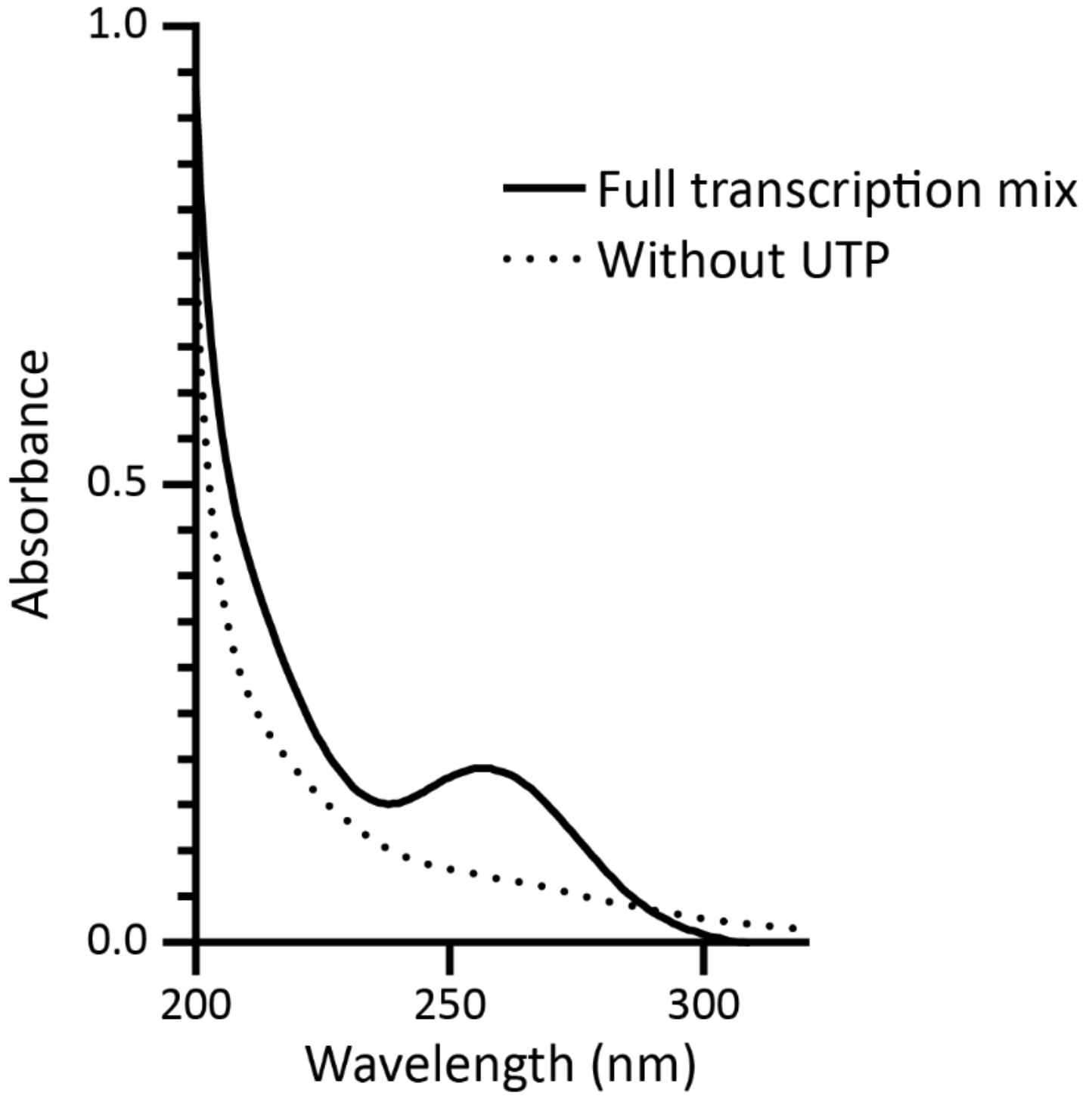

Supplementary Figure 1: Absorption spectrum of isolated VACV RNA. VACV cores were obtained by detergent-stripping of isolated and purified MVs, as described in Methods. 15 µl of cores were mixed with 15 µl transcription mix in a reaction tube (final concentration 1 mM GTP, 1 mM CTP, 1 mM UTP, 2.5 mM ATP, 10 mM DTT, 2mM $MgCl_2$) or an identical mixture without UTP. The mixture was incubated at 37°C for 30 min in a ThermoMixer (Eppendorf) to allow for transcription. RNA was isolated using RNeasy Plus Kits (QIAGEN) according to the manufacturer's protocol. Absorption was measured in a Nanophotometer (IMPLEN). The spectrum shows a specific absorption at 260 nm after incubation of the cores with a full reaction mix (solid line), compared to a reaction with a mix lacking one nucleotide necessary for transcription (dashed line).

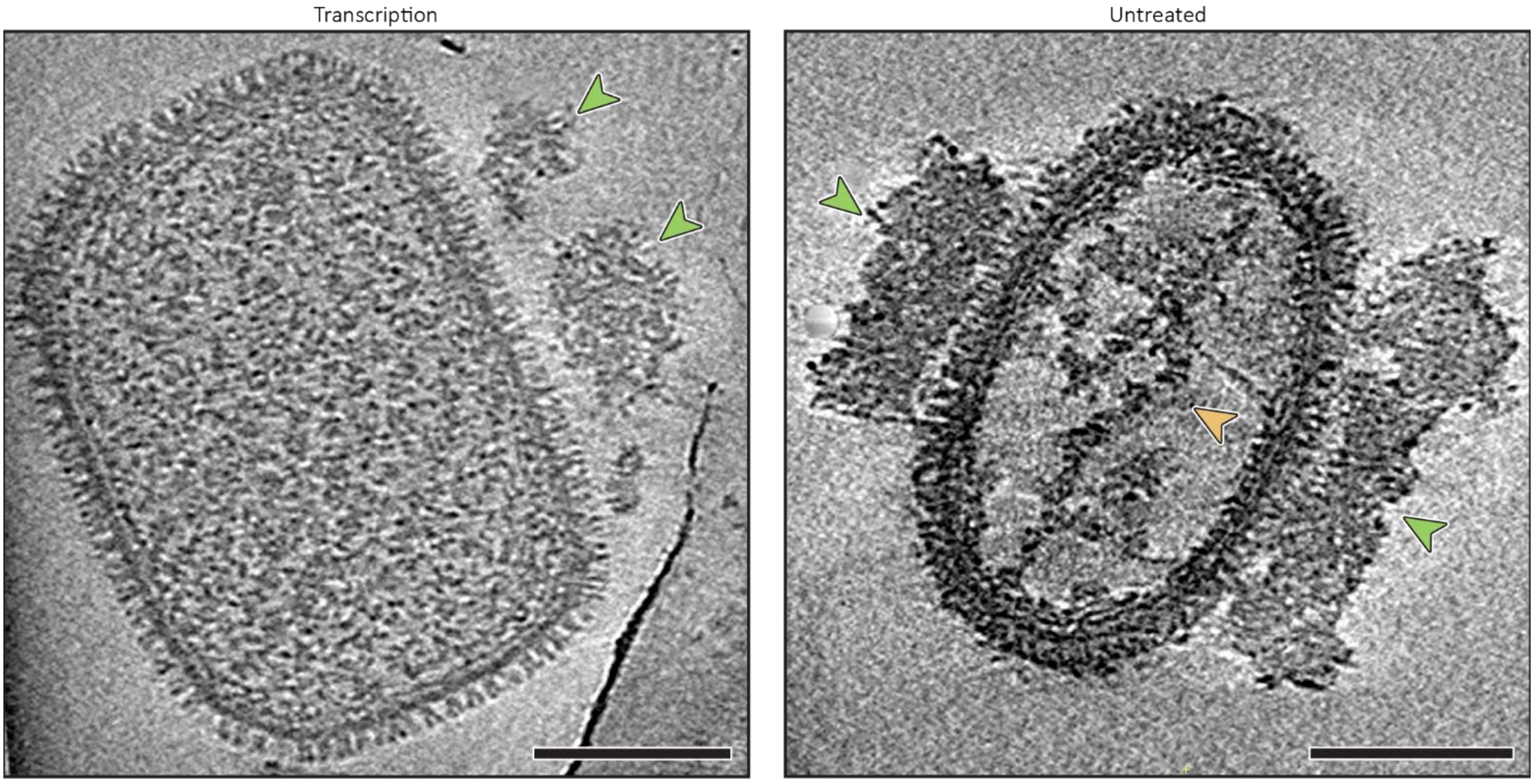

Supplementary Figure 2: Core morphology under transcribing and untreated conditions. Central slices through tomograms of VACV cores in the transcribing (left) and untreated (right) conditions. In the untreated condition the genome appears as a condensed density within the core (beige arrowhead), whereas in the transcribing condition the density is weaker and more evenly dispersed throughout the core interior. Green arrowheads indicate the lateral bodies, which remain in close apposition to the palisade layer in untreated cores but are separated from it in the transcribing condition. These are qualitative observations from the tomographic data. Scale bar 100 nm.

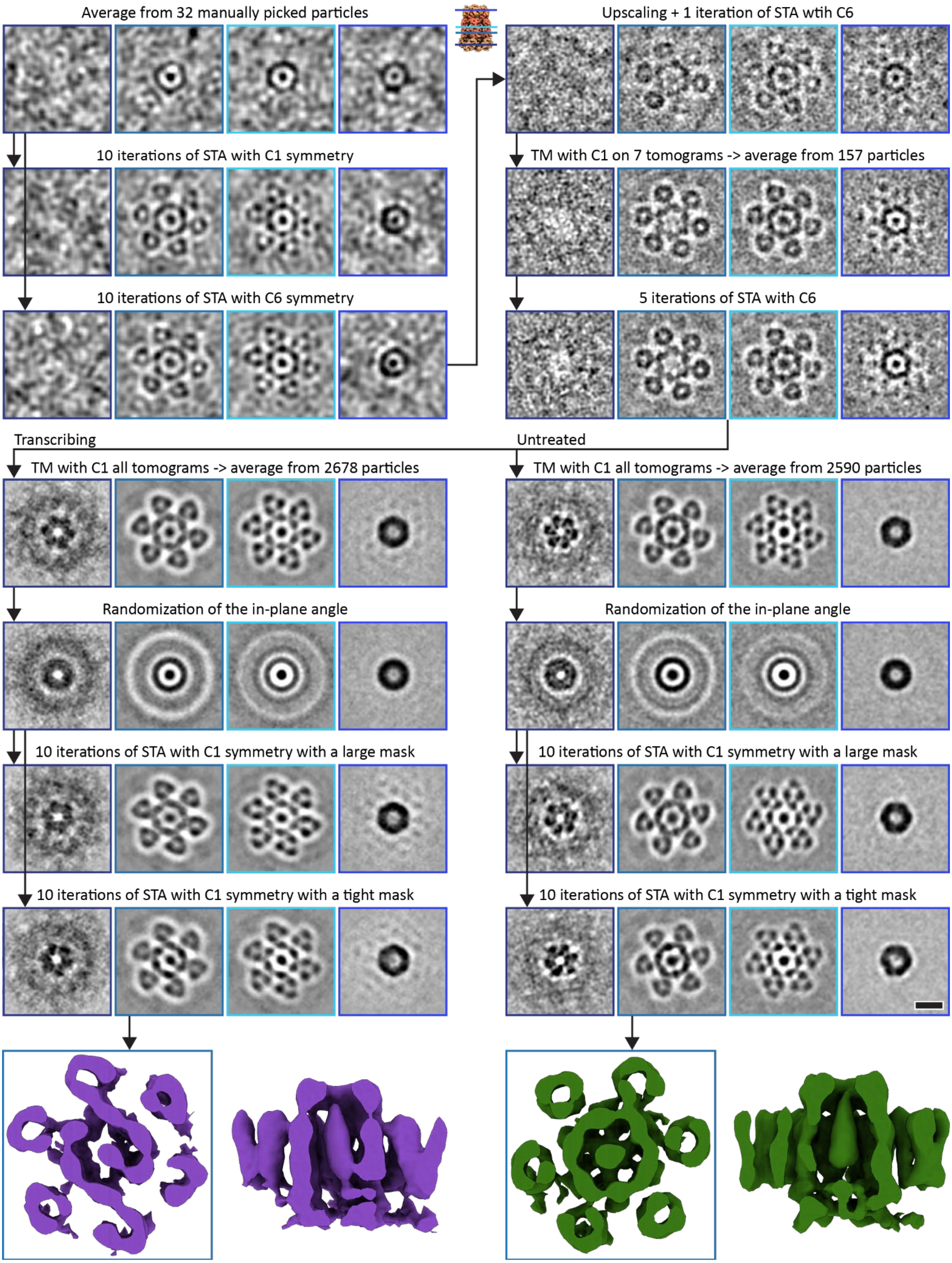

Supplementary Figure 3: Validation of the six-fold symmetry of the pore complex. XY slices through the averages at four heights (corresponding to the lines highlighted on the pore thumbnail). They correspond to the middle of E8, the middle of E6, the lower part of E6, and the middle of L3. The processing steps, labelled on the figure, are detailed in Supplementary Tables 9 and 10. Six-fold

features are visible in the C1 and C6 averages for the slices with E6 before the final template matching (TM), indicating that the symmetry is present without being imposed. For both the transcribing and untreated conditions, randomizing the in-plane angles of the final particle lists yields a featureless, tube-like average from which C1 alignment recovers the six-fold arrangement in all three protein layers; with the tight mask, the connection to the E6 wall is apparent in both conditions. The recovered symmetry for the untreated condition is not aligned with the original TM reference, further confirming the absence of C6 bias in the alignment. The bottom row shows isosurfaces of the tight-mask maps for each condition, viewed from the top (left) and from the side (right); the top views are clipped at the plane where a connection between the rod and the pore wall is apparent in the map, and the side views correspond to the same region. In the transcribing condition this connection is pronounced and located at the level of E6 and L3, whereas in the untreated condition it is not rendered at the isosurface threshold used, although a faint connection towards E8 is visible. These renderings are shown only to illustrate that the rod, and some of its connections to the pore, are preserved in the absence of imposed symmetry; no further conclusions are drawn from the differences between conditions. Scale bar, 10 nm.

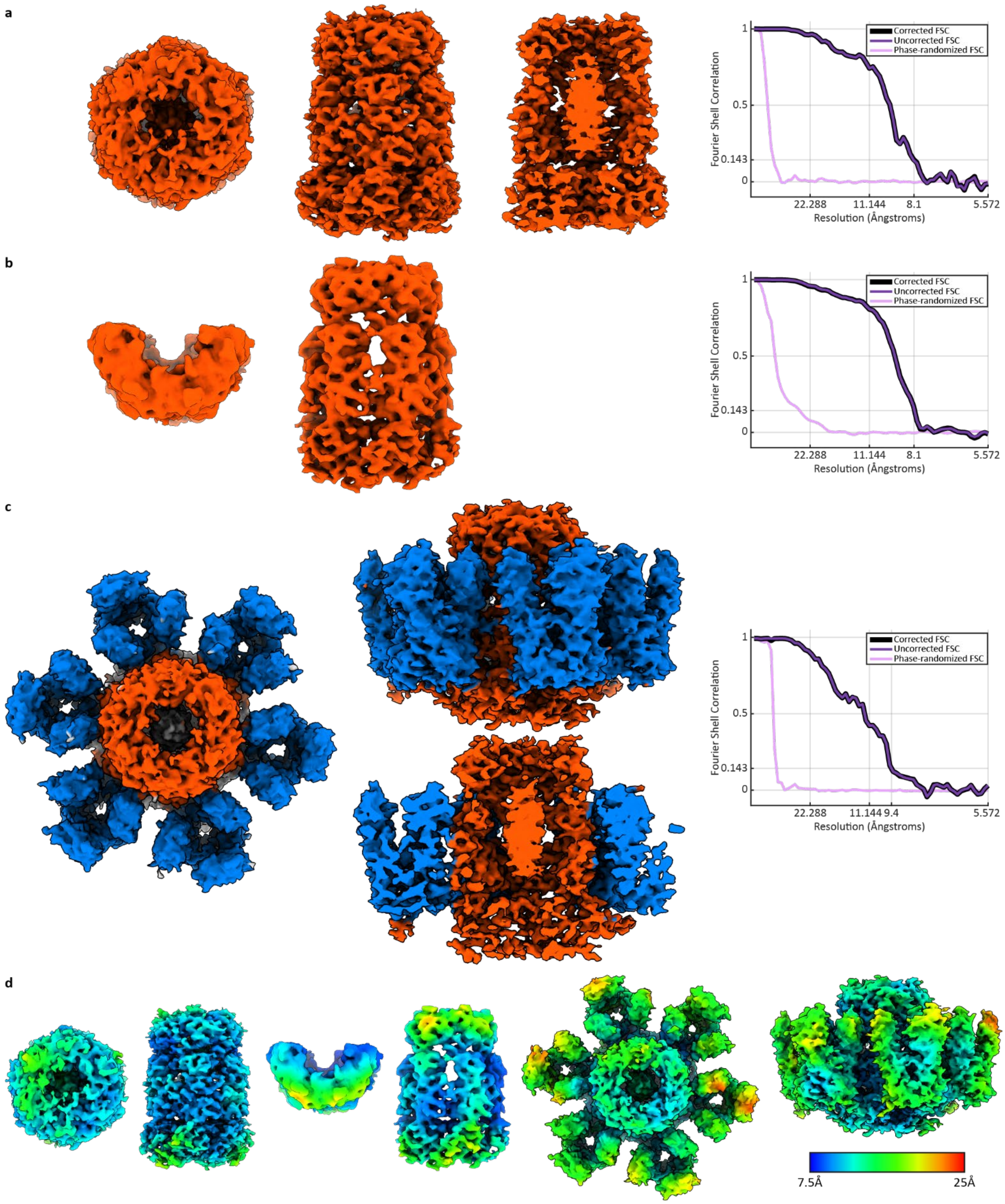

Supplementary Figure 4: Subtomogram averaging (STA) maps of the pore complex under transcribing conditions. a. STA map of the pore complex shown from the top (left) and side (middle). The adjacent panel shows a central slice through the map, revealing a rod-like density within the pore. The Fourier shell correlation (FSC) curve is shown on the right. **b.** STA map of an asymmetric subunit of the pore complex, displayed from the top (left) and side (middle), with the corresponding FSC curve on the right. As the protomer is slightly twisted, a cylindrical alignment mask was used rather than a tight, shaped one, so the map also includes parts of the neighboring protomers. **c.** STA map of the pore complex (dark orange) together with surrounding A10 trimers (blue). Shown are the

top view (left), side view (middle, top), and a cut-open side view (middle, bottom). The corresponding FSC curve is displayed on the right. A10 trimers were fitted into the map using UCSF ChimeraX to assign densities based on proximity (12 Å cutoff). **d.** The same maps as in (a–c), colored by local resolution. The local-resolution scale (7.5–25 Å) is shared across all local-resolution maps in this manuscript to allow direct comparison between reconstructions.

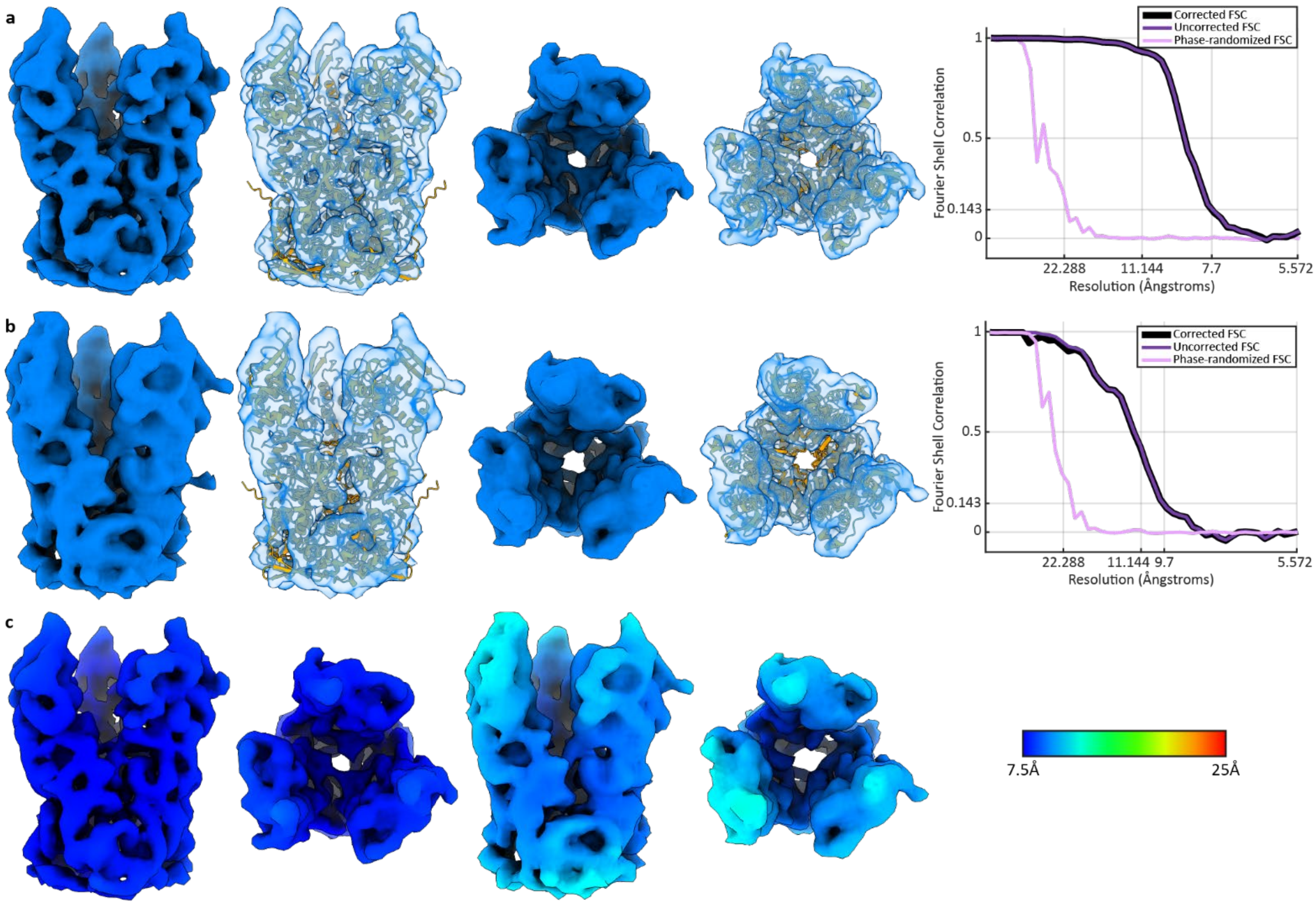

Supplementary Figure 5: Subtomogram averaging (STA) maps of A10 trimers under transcribing conditions. a. STA map of all A10 trimers (number of particles: 168,352). Left: iso-surface rendering of the sharpened map with the fitted atomic model (PDB 8P4K) shown alongside. Middle: corresponding top views. Right: Fourier shell correlation (FSC) curve indicating the resolution of the reconstruction. **b.** Same as in (A), but for the subset of A10 trimers surrounding the pore complexes (total number of particles: 16,068). **c.** The same maps as in (a–b), colored by local resolution. The local-resolution scale (7.5–25 Å) is shared across all local-resolution maps in this manuscript to allow direct comparison between reconstructions.

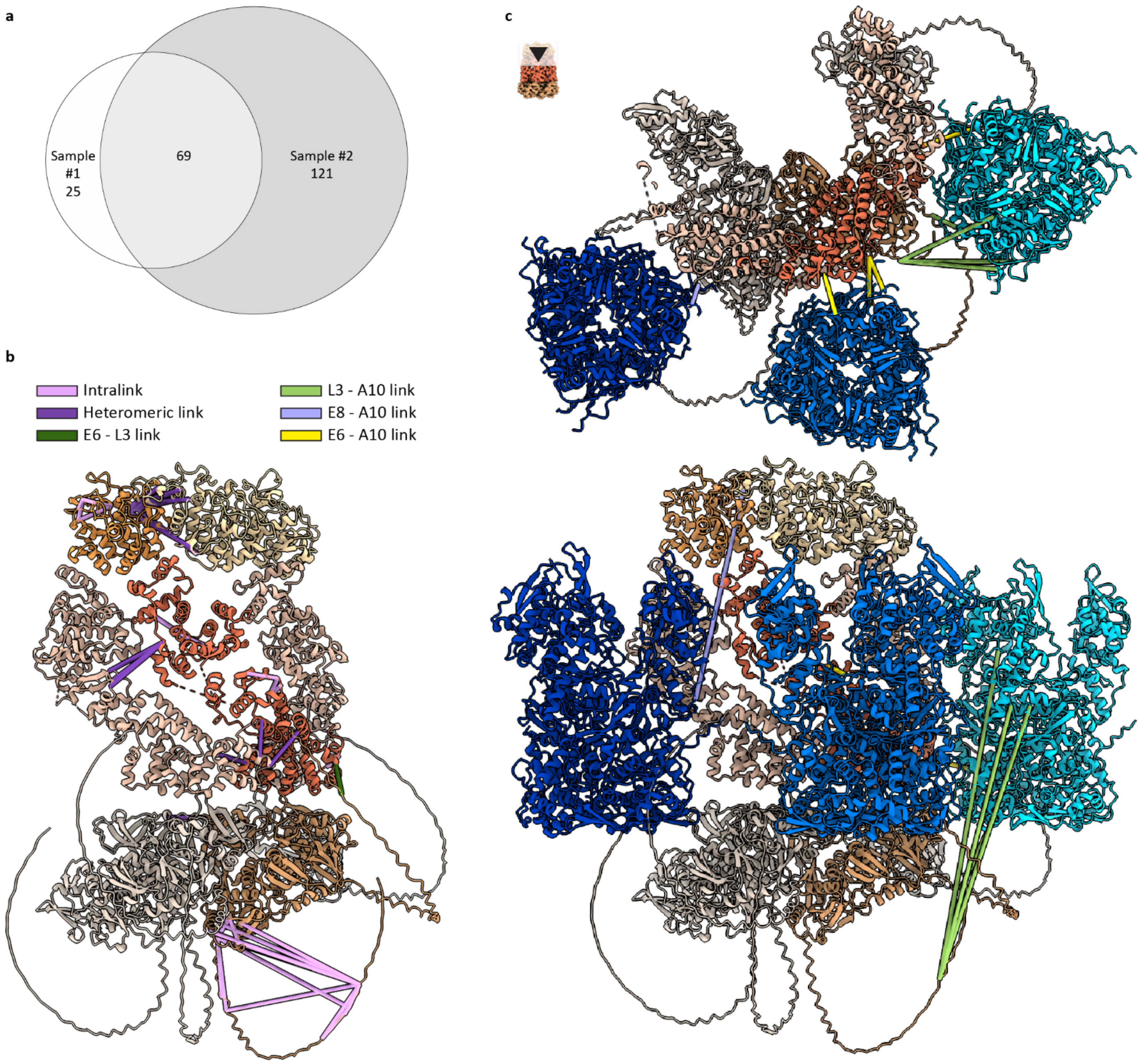

Supplementary Figure 6: Cross-linking mass spectrometry (XL-MS) analysis. a. Unique and shared protein–protein interactions (PPIs) identified in the two VACV core samples analyzed by XL-MS at 1% false discovery rate (FDR). **b–c**. Cross-links mapped onto structural models. (b) Cross-links within the pore complex. (c) Cross-links between the pore complex and the model containing A10. Cross-links were assigned based on minimal inter-chain distances, selecting the shortest distances between corresponding subunits within each ring. The same distance-based criterion was applied to both intra-chain links and heteromeric links within the hexameric/dodecameric assemblies. Models are colored by protein identity: the E8 hexamer is shown in apricot (one protomer in a darker shade, remaining subunits in lighter shades), the E6 hexamer in orange (one protomer highlighted, others lighter), and the L3 dodecamer in cappuccino tones (one protomer highlighted, others lighter). The A10 trimers are colored as follows: left trimer in navy blue, central trimer in blue, and right trimer in cyan. Source data are provided as a Source Data file.

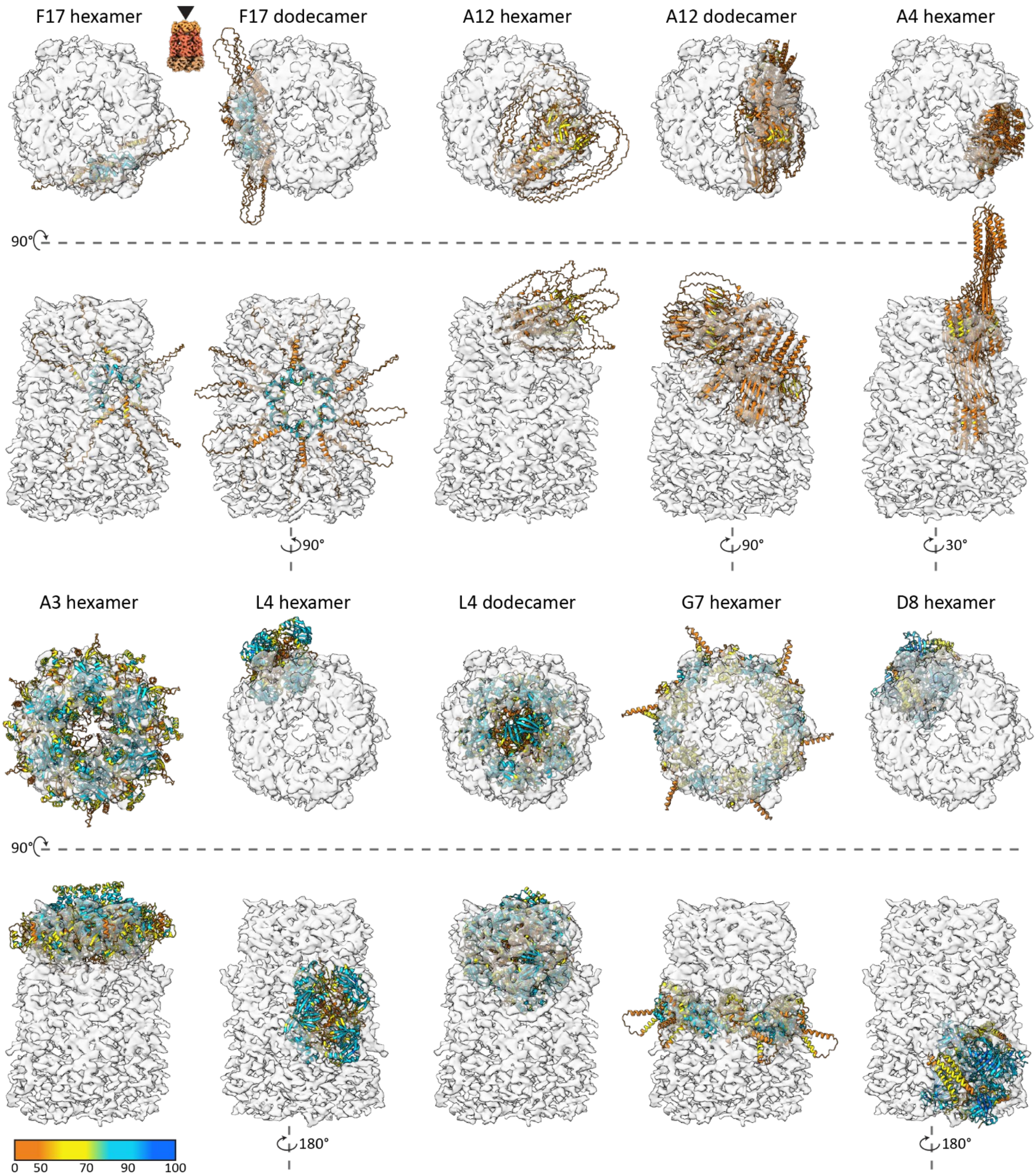

Supplementary Figure 7: Fitting of alternative candidate proteins into the pore density. Representative AF3 predictions of A10 cross-linking partners in the oligomeric states indicated, after global fitting into the STA density (grey). For each candidate, the top view (upper row) and side view (lower row) are shown; the direction of view is indicated on the thumbnail (top left, arrowhead). Additional rotations applied for clarity are indicated by the dashed axes and corresponding angles. Models are colored by per-residue confidence (predicted Local Distance Difference Test, pLDDT), with values indicated on the scale bar. In each case the candidate either fails to fill the pore density, extends substantially beyond it, or does not match its shape; none of the alternative candidates accounts for the density to the extent of the assigned components E6, E8 and L3.

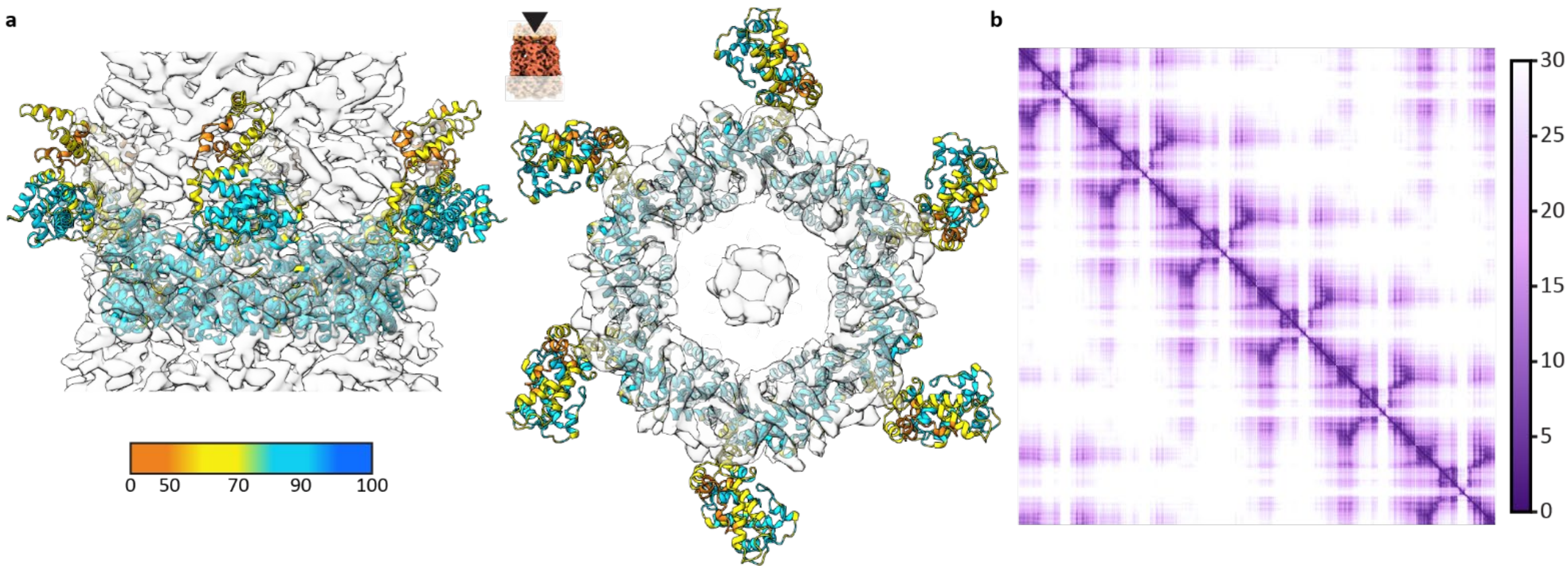

Supplementary Figure 8: Fitting of the E6 AlphaFold3 prediction into the pore STA map. a. AlphaFold3 (AF3) prediction of the E6 hexamer fitted into the sharpened pore complex map. The model is colored by per-residue confidence (predicted Local Distance Difference Test; pLDDT), with values indicated on the scale bar. The interface predicted template modeling (ipTM) score is 0.4. Both side (left) and top (right) views show that the upper region of the model, corresponding to lower confidence scores, extends beyond the experimentally resolved density. **b.** Predicted Aligned Error (PAE) plot for the AF3 model. The PAE plot indicates the expected positional error between residue pairs, with lower values (darker colors) reflecting higher confidence in the relative positioning and higher values (lighter colors) indicating increased uncertainty, particularly in flexible or poorly resolved regions.

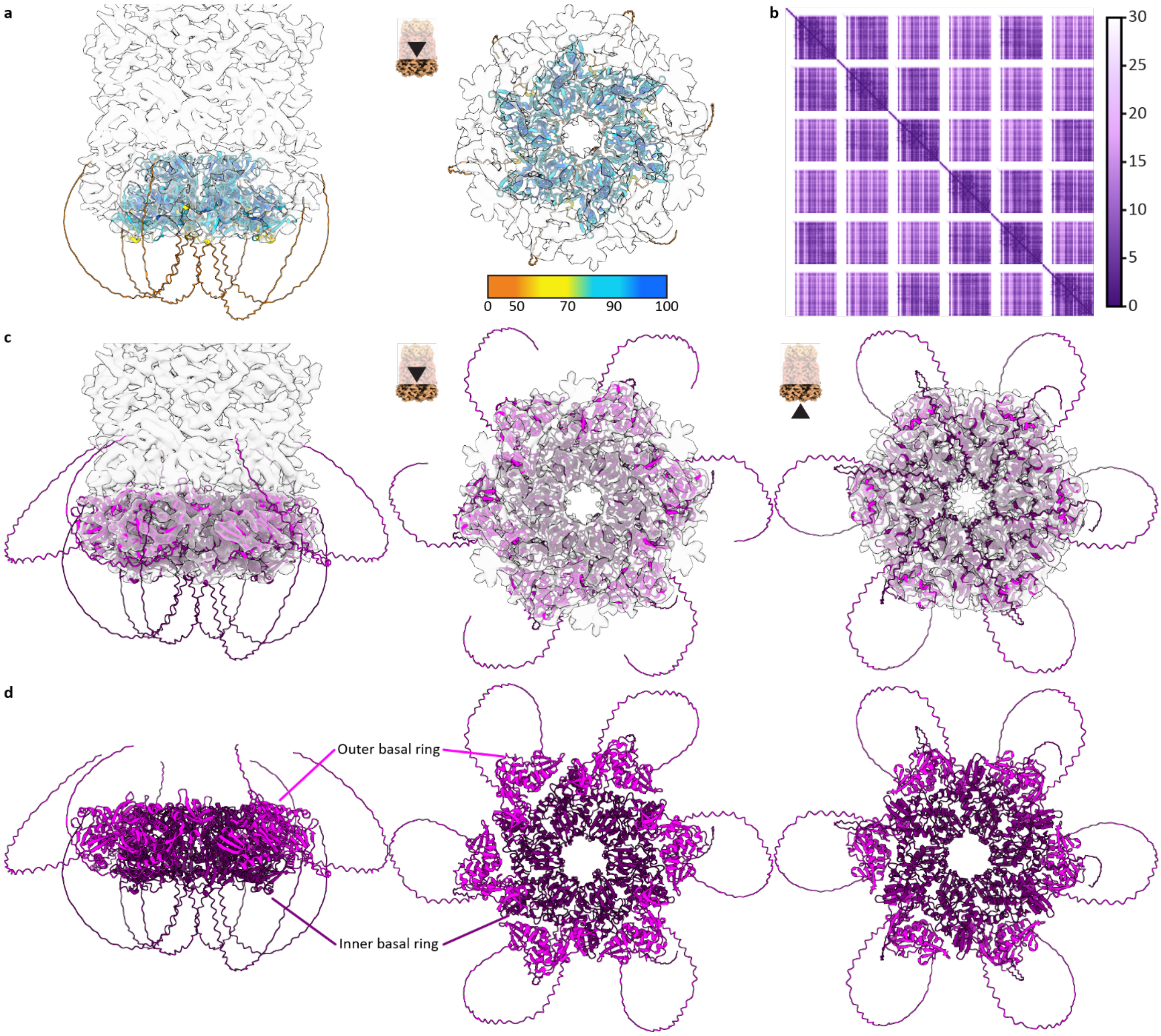

Supplementary Figure 9: Fitting of the L3 AlphaFold3 prediction into the pore STA map. **a.** AF3 prediction of the L3 hexamer fitted into the sharpened pore complex map. The model is colored by per-residue confidence (pLDDT), with values indicated on the scale bar and ipTM for this prediction is 0.73. While the overall fit of the model to the density is reasonable, substantial regions of the map remain unoccupied, suggesting that additional components or conformations contribute to the observed density. The N-terminal region (residues 1–56) shows lower confidence and likely represents a flexible segment that is not well resolved in the map. Side (left) and top (right) views are shown. **b.** Predicted Aligned Error (PAE) plot for the AF3 model. The PAE plot indicates variable confidence across the structure, with higher predicted errors consistent with flexible or less well-defined regions, including the N-terminus. Residues corresponding to the original AF3-predicted hexamer (inner basal ring) are shown in violet, while the additional fitted protomers forming the outer basal ring are shown in magenta. **c.** L3 dodecamer fitted into the density, shown in side (left), top (middle), and bottom (right) views, illustrating the overall agreement between the model and the experimental map. The inner basal ring (original AF3 hexamer) is colored violet, and the newly fitted outer ring protomers are colored magenta. **d.** Same views as in (c), but with the density omitted to show the isolated L3 dodecamer model.

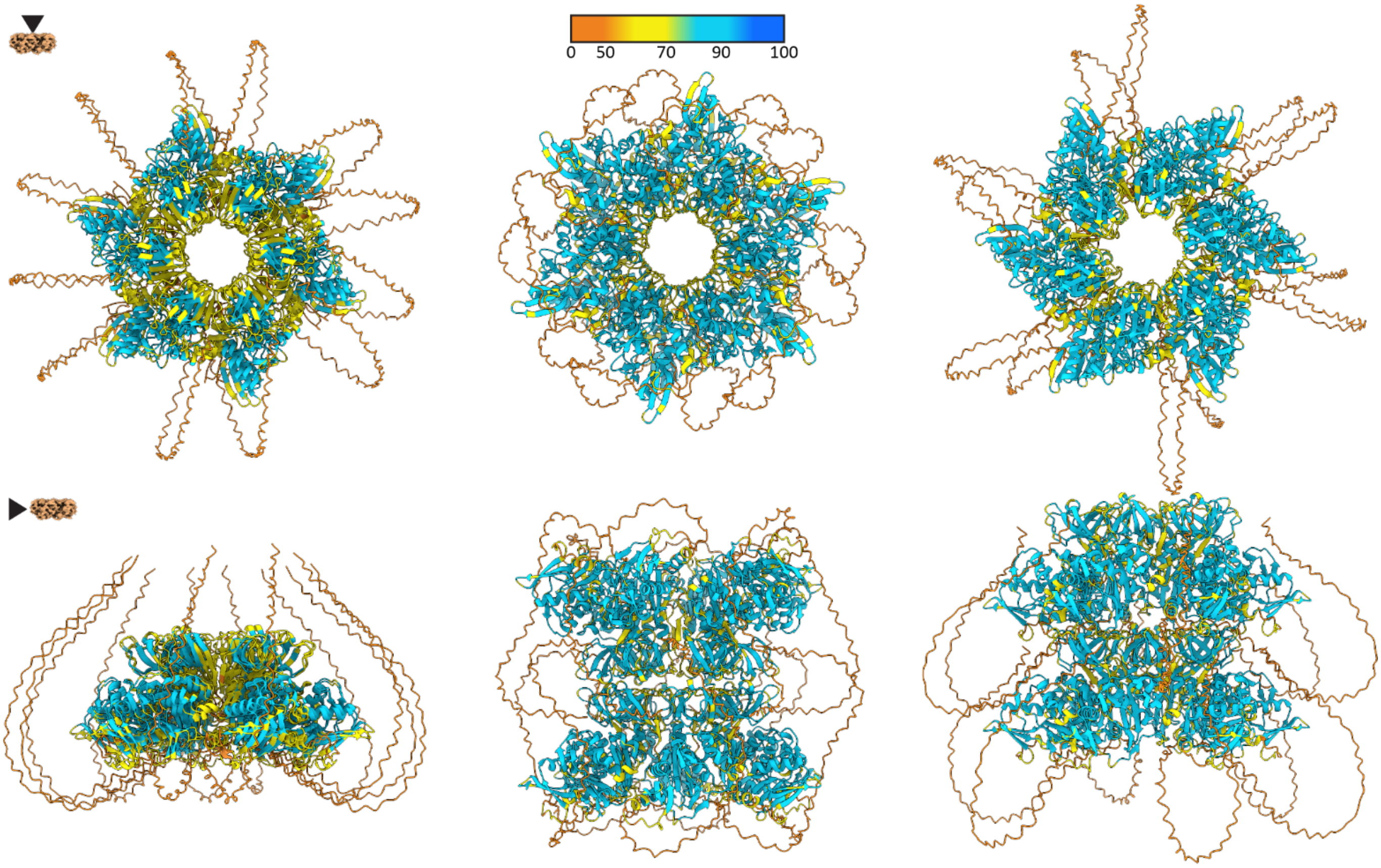

Supplementary Figure 10: AF3 predictions of the L3 dodecamer. Three representative AF3 predictions of a dodecameric L3 assembly, shown as top views (upper row) and side views (lower row), colored by per-residue confidence (predicted Local Distance Difference Test, pLDDT; scale shown in the figure). Left: all twelve chains occupy approximately the space of a single hexameric ring, giving a highly overlapping arrangement. Middle: two hexameric rings stacked with their upper faces towards each other. Right: two hexameric rings stacked one above the other. None of the predictions reproduces the concentric double-ring arrangement observed in the density, which was therefore built in a density-guided manner.

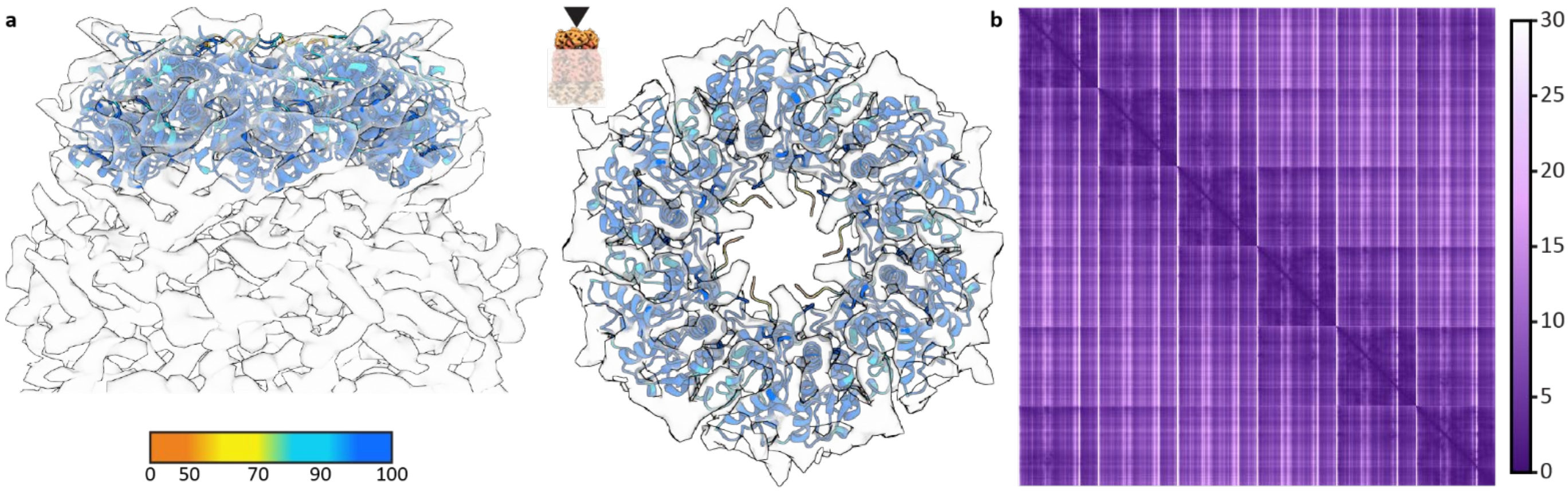

Supplementary Figure 11: Fitting of the E8 AlphaFold3 prediction into the pore STA map. a. AF3 prediction of the E8 hexamer fitted into the sharpened pore complex map. The model is colored by per-residue confidence (pLDDT), with values indicated on the scale bar. The ipTM is 0.87. Both side (left) and top (right) views demonstrate an excellent agreement between the predicted model and the experimental density, with the structure closely matching the map throughout. High-confidence regions correspond well to well-resolved density, indicating a reliable fit. **b.** Predicted Aligned Error (PAE) plot for the AF3 model. The PAE plot shows consistently low error values across most residue pairs, supporting high confidence in the relative positioning of domains and overall structural organization.

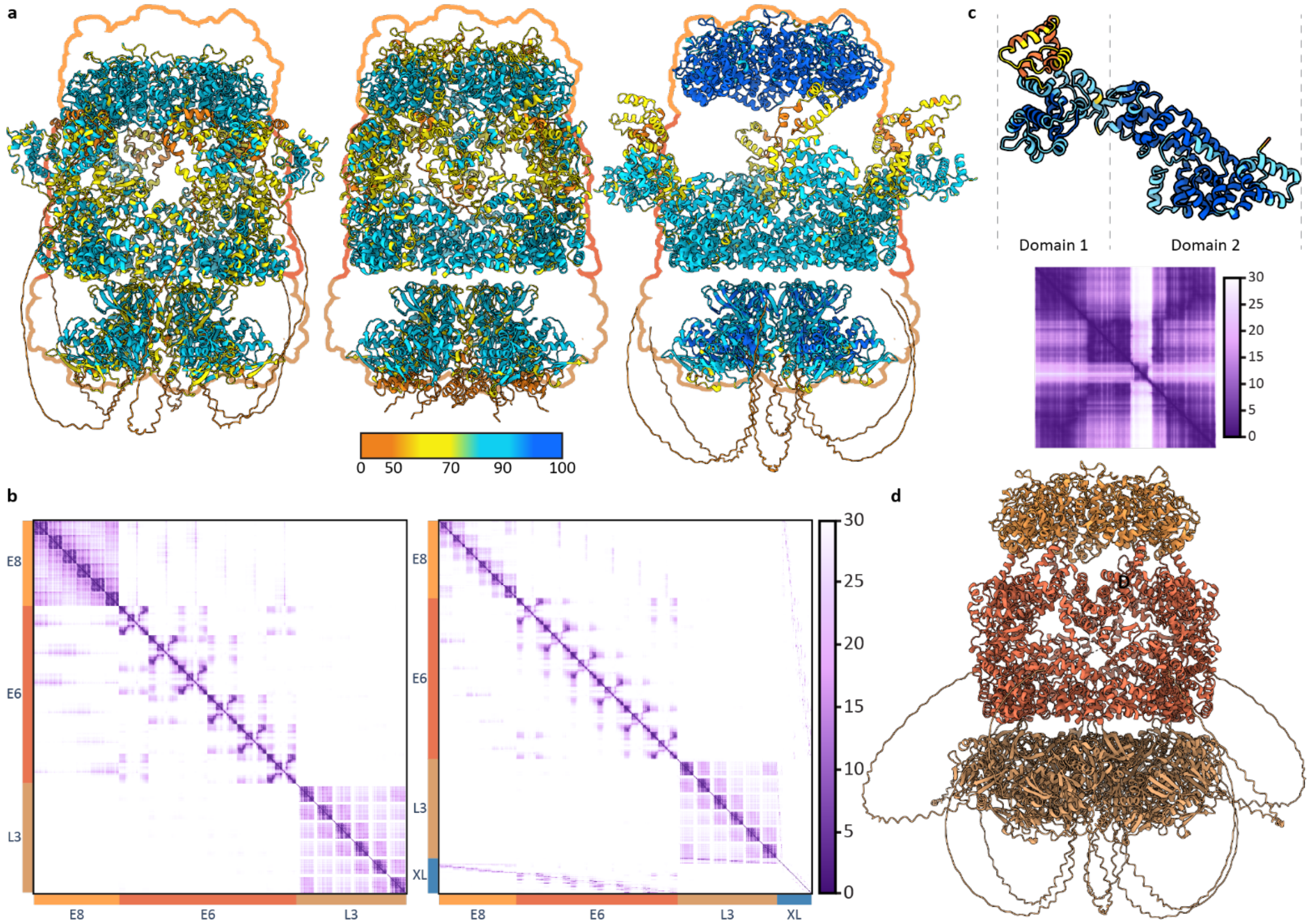

Supplementary Figure 12: AlphaFold3 and AF3x predictions of the pore complex. a. AF3 prediction of the complete pore complex, shown using the L3 hexamer (the dodecamer assembly did not converge reliably and resulted in substantial steric clashes). The colored silhouette represents the structure obtained by STA. The AF3 model shows correct relative positioning of the hexameric assemblies; however, the vertical spacing between them is compressed compared to the experimental density. In addition, the E6 hexamer adopts an open conformation, similar to the isolated prediction, and does not fully account for the observed density. The ipTM for this prediction is 0.41. The AF3x prediction (middle) is shown for comparison against the same STA-derived silhouette (ipTM is 0.39). In this case, the spacing between the L3 and E6 hexamers agrees better with the map, although the distance between E6 and E8 remains too short. While the E6 hexamer appears more closed in its upper region, it still does not fully match the STA density. Individual hexamer predictions fitted into the map are shown on the left. All models are colored by per-residue confidence (pLDDT), with values indicated on the scale bar. **b.** Predicted Aligned Error (PAE) plots corresponding to the models in (a): AF3 (left) and AF3x (right). PAE plots for the individual hexamer predictions are shown in Supplementary Figures 8 (E6), 9 (L3), and 11 (E8). **c.** AF3 prediction of the E6 monomer, colored by pLDDT as in (a), with the corresponding PAE plot. Domain 2 shows good agreement with the experimental density, whereas Domain 1 extends beyond the density (as also observed in a) and was positioned into the map by rigid-body fitting. **d.** Final model of the pore complex assembled from individual AF3 predictions: E8 hexamer (apricot), the fitted E6 model (orange), and L3 (cappuccino). An L3 protomer from the hexameric prediction was fitted into the remaining density and expanded with C6 symmetry in UCSF ChimeraX to generate the dodecameric assembly.

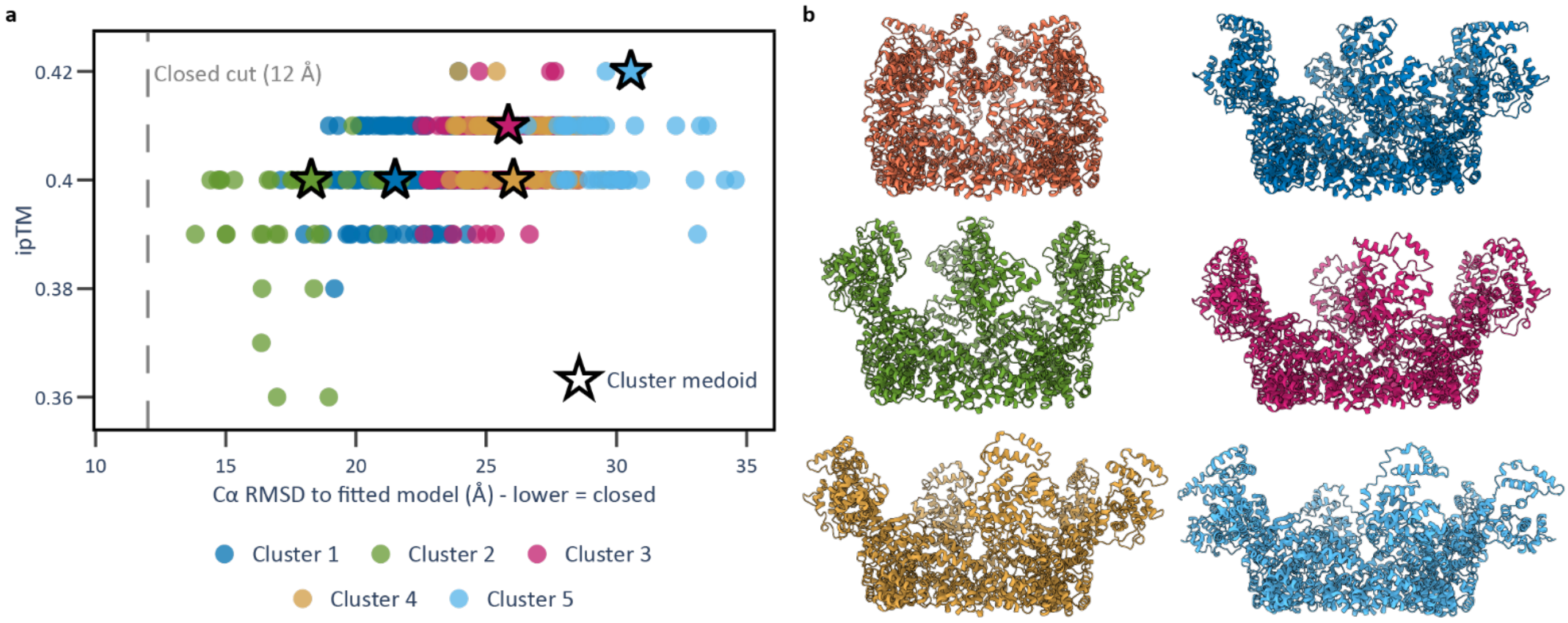

Supplementary Figure 13. AF3 seed scan of the E6 hexamer. 500 AF3 predictions of the E6 hexamer, generated across 100 independent seeds, evaluated for symmetry and conformation (Methods). **a.** Cα RMSD of each prediction to the fitted E6 model (lower = closer to the closed conformation) versus ipTM, colored by conformational cluster (agglomerative clustering, 1–5); stars mark cluster medoids and the dashed line the closed-conformation cut (12 Å), n = 500. All predictions form regular six-fold rings but none reaches the closed state (minimum 13.8 Å, median 24.7 Å RMSD), at uniformly low ipTM. **b.** Side views of the representative prediction from each cluster, colored as in (a), with the fitted E6 model (orange) shown first for reference. The predicted rings are consistently more open than the fitted model; cluster 2 is the least open. Source data are provided as a Source Data file.

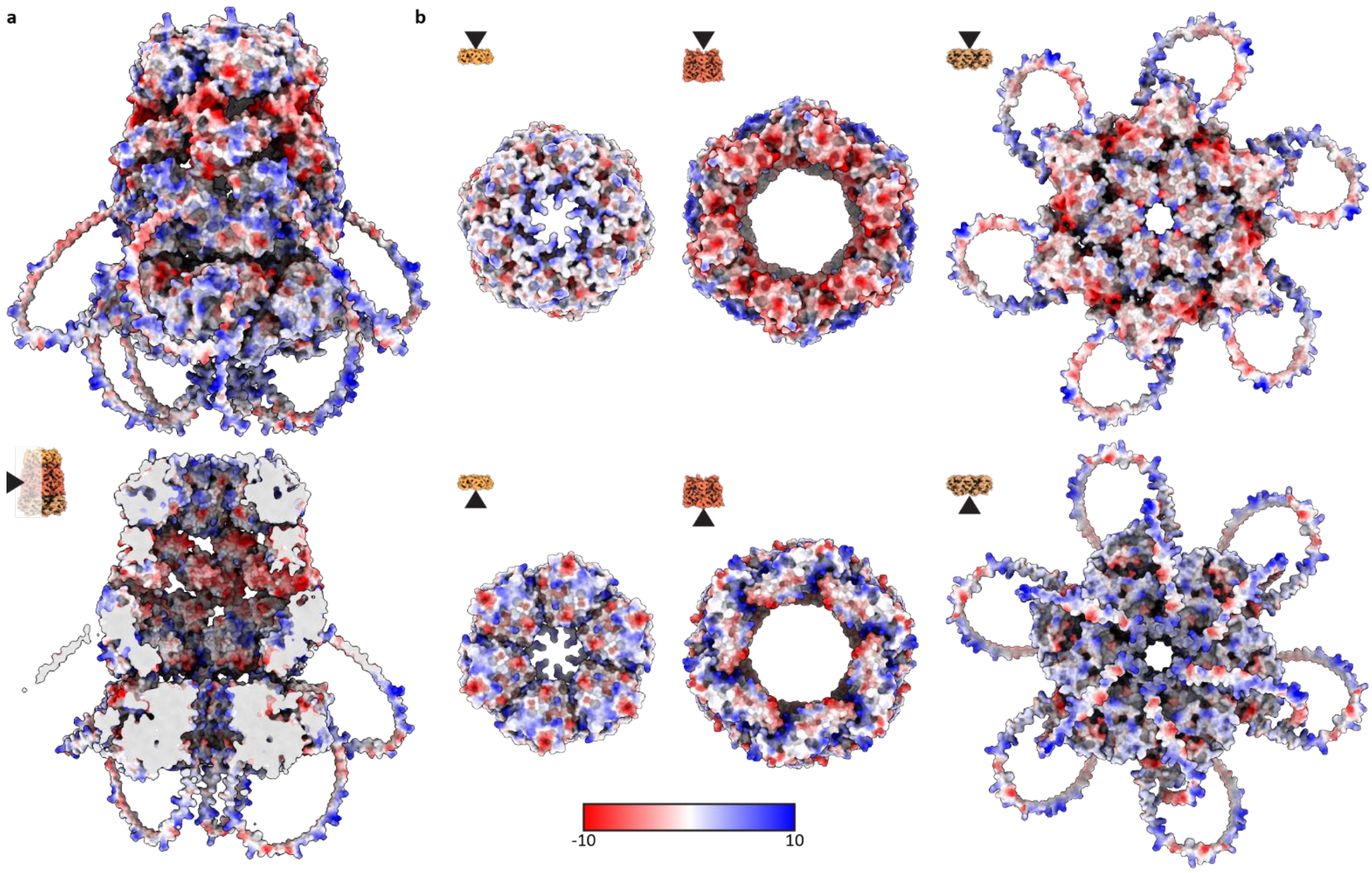

Supplementary Figure 14: Coulombic surface potential of the pore complex. a. Side view of the assembled pore complex model composed of the E8 hexamer, E6 hexamer, and L3 dodecamer. The top panel shows the intact structure, while the bottom panel displays a cut-open view to reveal the inner channel. The Coulombic surface potential is shown on a red–blue scale (red, negative; blue, positive), with values indicated on the scale bar. The channels of E8 and L3 exhibit a strongly positive potential, whereas the upper region of the E6 hexamer is predominantly negatively charged, including a thin negatively charged ring at its base. **b.** Top (upper row) and bottom (lower row) views of the individual ring assemblies, shown in the order E8, E6, and L3. The electrostatic potential distribution highlights distinct charge properties of each ring and their contributions to the overall pore environment.

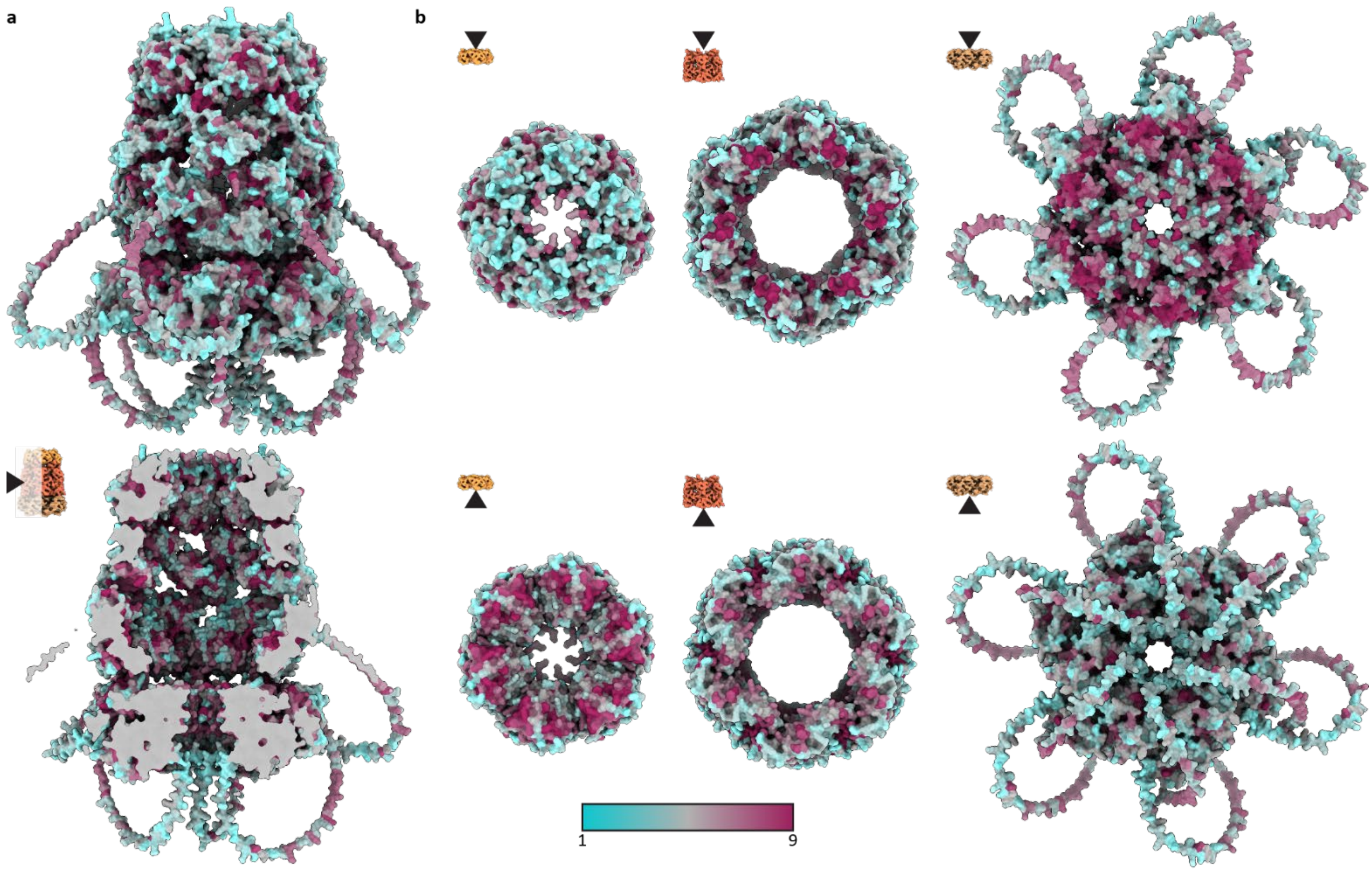

Supplementary Figure 15: Evolutionary conservation of the pore complex. a. Side view of the assembled pore complex model composed of the E8 hexamer, E6 hexamer, and L3 dodecamer. The top panel shows the complete assembly, while the bottom panel displays a cut-open view to reveal the inner channel. Residues are colored by ConSurf conservation grade on a scale from 1 (most variable) to 9 (most conserved), with values indicated on the scale bar. Conservation grades were computed on the individual monomers, without reference to the structural model, the density, or the oligomeric state (see Methods). **b.** Top (upper row) and bottom (lower row) views of the individual ring assemblies, shown in the order E8, E6, and L3. The conservation pattern highlights the distribution of evolutionarily constrained residues across each ring.

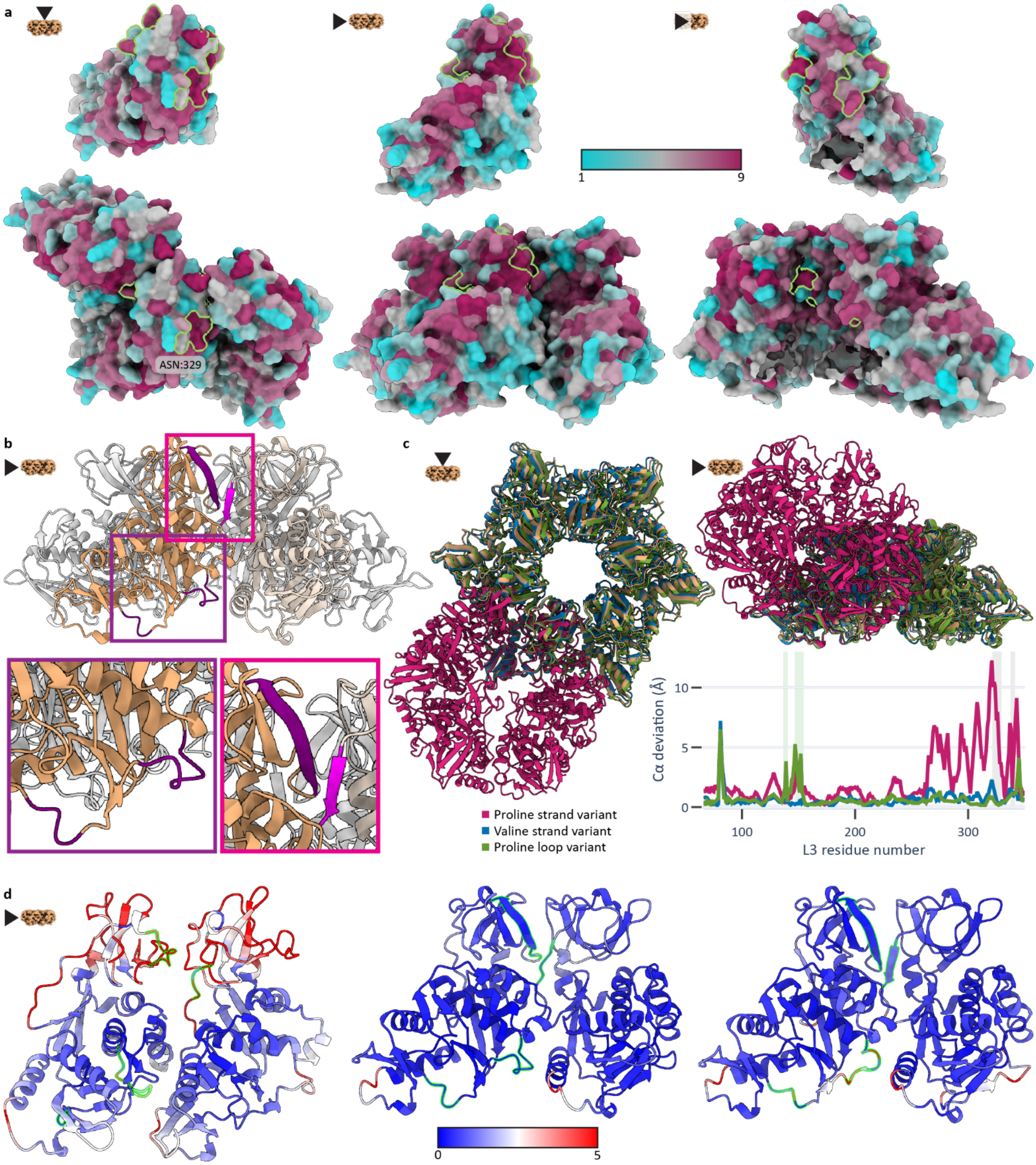

Supplementary Figure 16: *In silico* substitution analysis of the inter-protomer β-sheet in the predicted L3 hexamer. a. Surface representation of a single L3 protomer (top row) and of three adjacent protomers (bottom row), colored by ConSurf conservation grade (1 = most variable, 9 = most conserved; scale bar), shown from the top (left), outside (middle) and channel (right). Residues of the inter-protomer β-sheet region (strands 322-329 and 338-341, including the conserved acidic residues Asp319, Glu324, Glu326, Asp327 and Glu338) are outlined in light green. The conserved patch is exposed on the isolated protomer but becomes occluded in the assembly, with the single residue remaining exposed (Asn329) among the least conserved – the sequence signature expected of a protein-protein interface. Conservation was computed on the monomer alone, independently of the model, density or oligomeric state. **b.** AlphaFold3 predictions of the wild-type L3 hexamer and

three substitution variants (Methods). Wild-type hexamer, side view, with two neighboring protomers highlighted: violet marks the substituted regions of one protomer – the longer strand (322-329) and both control loops (137-140, 147-154); pink marks the shorter strand (338-341) from the neighbor. Boxed regions are enlarged below, showing the strands pairing across the interface and the control loops lying away from it. **c.** Superposition of the four predicted hexamers: wild type (cappuccino), proline-strand (dark pink), valine-strand (blue) and proline-loop (green); top (left) and side (right) views. The valine-strand and proline-loop variants reproduce the wild-type assembly (whole-ring Cα RMSD 1.05 and 1.11 Å), whereas the proline-strand variant forms a hexamer whose protomers adopt a different orientation, so the two rings cannot be brought into correspondence. Bottom right, per-residue Cα deviation from wild type, averaged over the six protomers, colored as above; shaded bands mark the substituted strands and control loops. **d.** Two adjacent protomers of each variant (proline-strand, valine-strand, proline-loop; left to right), colored by per-residue Cα deviation from wild type (0–5 Å; scale bar), with substituted regions in lime. Residues 1-65 are predicted disordered and not shown. Source data are provided as a Source Data file.

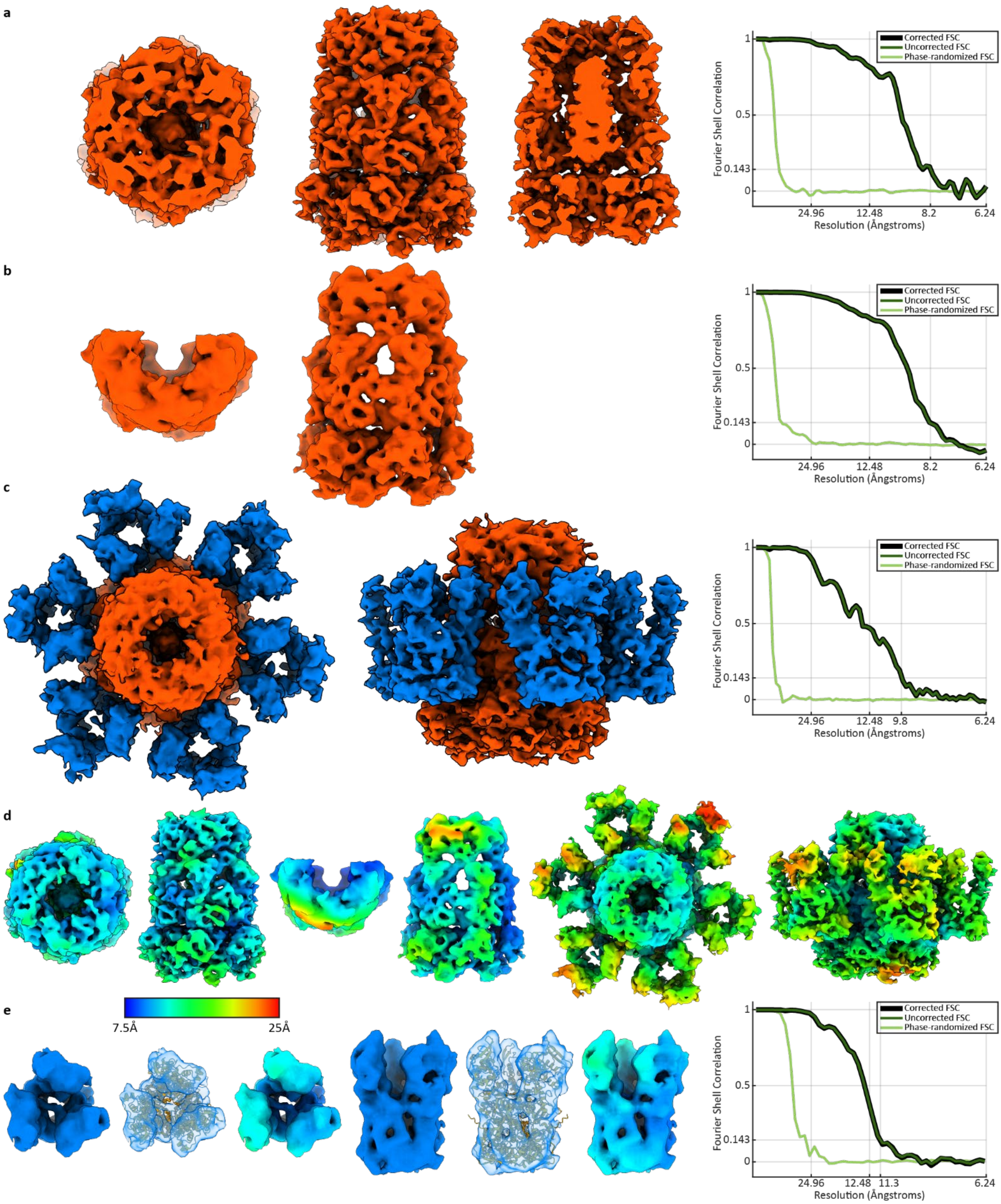

Supplementary Figure 17: STA maps of the pore complex under untreated condition. a. STA map of the pore complex shown from the top (left) and side (middle). The adjacent panel shows a central slice through the map, revealing a rod-like density within the pore. The FSC curve is shown on the right. **b.** STA map of an asymmetric subunit of the pore complex, displayed from the top (left) and side (middle), with the corresponding FSC curve on the right. **c.** STA map of the pore complex (dark orange) together with surrounding A10 trimers (blue). Shown are the top view and the side view. The corresponding FSC curve is displayed on the right. A10 trimers were fitted into the map using UCSF

ChimeraX to assign densities based on proximity (12 Å cutoff). **d.** The maps from (a–c) colored by local resolution, on a scale (7.5–25 Å) shared across all local-resolution maps in this manuscript to allow direct comparison between reconstructions. **e.** STA map of A10 trimers surrounding the pore complexes (total number of particles: 15,540). Left: top view of the iso-surface rendering of the sharpened map with the fitted atomic (PDB 8P4K) model and local resolution shown alongside. Middle: corresponding side views. The local resolution has the same range as in (d). Right: FSC curve indicating the resolution of the reconstruction.

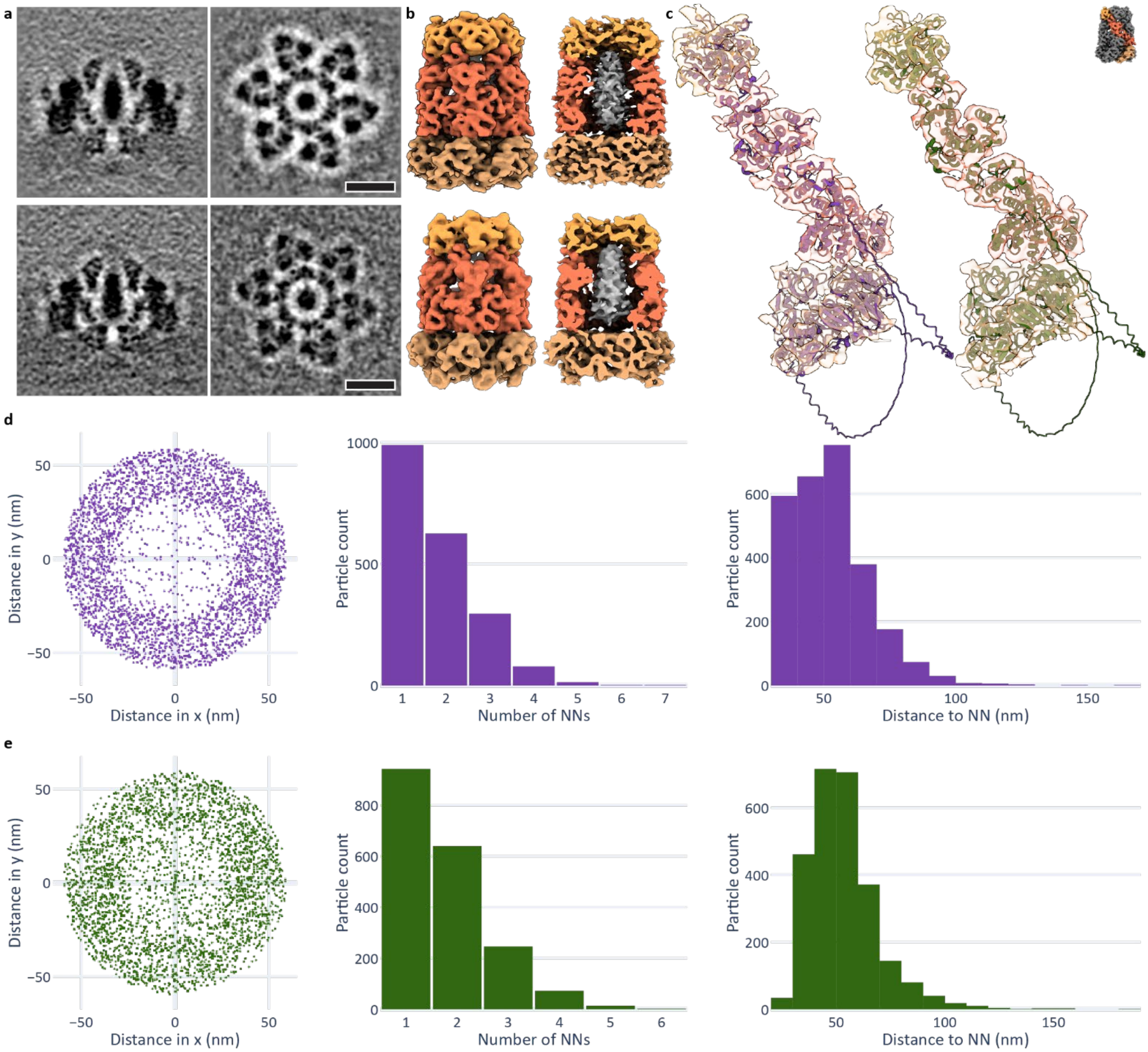

Supplementary Figure 18: Comparison of the transcription and untreated condition. a. Density maps of the pore complex in transcribing cores (top) and untreated cores (bottom). The side views on the left show the resemblance of the pore structure in both conditions. **b.** Iso-surface representation of the pore STA maps shown in A, seen from the side fully (left) and cut, revealing the similar internal density of the inner rod (right). **c.** Our model fitted into the density corresponding to one asymmetric subunit of E8, E6, and L3 of the structure obtained from the untreated (left) and the treated (right) dataset. **d-e.** Contextual analysis of transcription (d) and untreated (e) conditions. **d.** Spatial organization of pore complexes under transcribing conditions. Left: distribution of nearest neighbors (NN) in the XY plane within a radius of 60 nm, showing no apparent spatial pattern and suggesting a random distribution of pores across the core surface. Middle: histogram of the number of nearest neighbors per pore within the 60 nm radius. Right: histogram of distances to the nearest neighbor for each pore without applying a distance cutoff. **e.** Same analysis as in (d), shown for untreated cores. Source data are provided as a Source Data file.

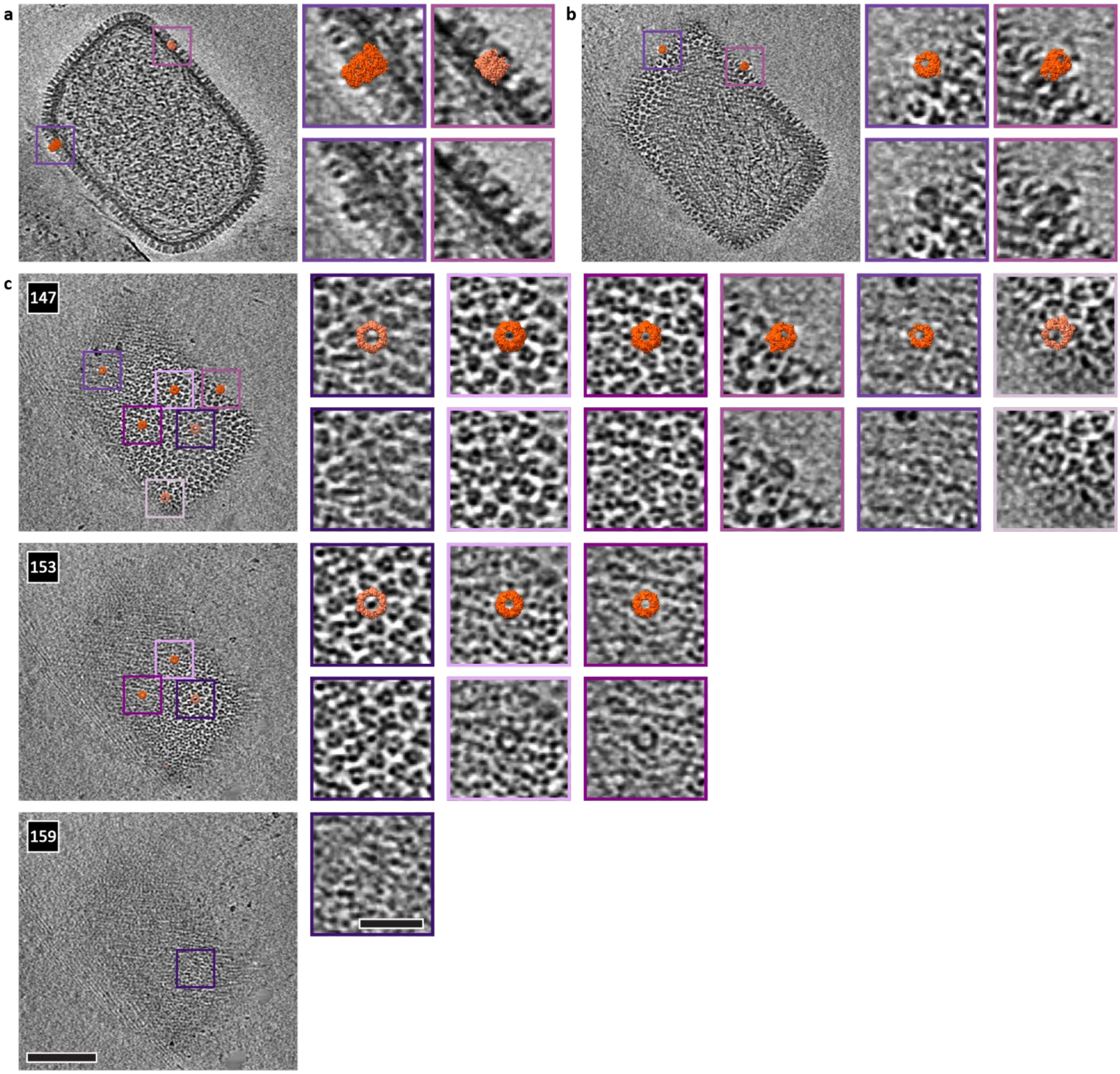

Supplementary Figure 19: Visualization of capped and uncapped pore complexes in tomograms. a. Tomographic slice with subtomogram averages of pore complexes placed back into the volume. Fully assembled pores are shown in dark orange, while uncapped pores (lacking the L3 and E8 rings, as determined by classification) are colored in light orange. Two regions are indicated and shown as zoomed-in views: the top row displays the tomogram with the maps, while the bottom row shows the corresponding raw tomogram slices. The absence of the E8 ring density is clearly visible in the uncapped pore (right). **b.** Same representation as in (a), but showing only fully assembled pore complexes. Prominent ring-like densities corresponding to the E8 layer are clearly visible. **c.** Sequential tomographic slices illustrating the vertical organization of pore complexes. The first row shows four fully assembled pores and two uncapped pores. The numbers indicate the z-height of each slice (pixel size: 11.144 Å). The second row shows a slice shifted by ~6.7 nm relative to the first, where the central fully assembled pores are now at the level of the E8 ring, which is clearly visible, while the uncapped pore remains also visible. The third row shows an additional ~6.7 nm shift. At this level, the E8 ring would be expected for fully assembled pores; however, it is absent in

the uncapped pore, confirming the lack of this structural feature. Scale bars: 100 nm (overview slices) and 30 nm (zoomed-in views).

a

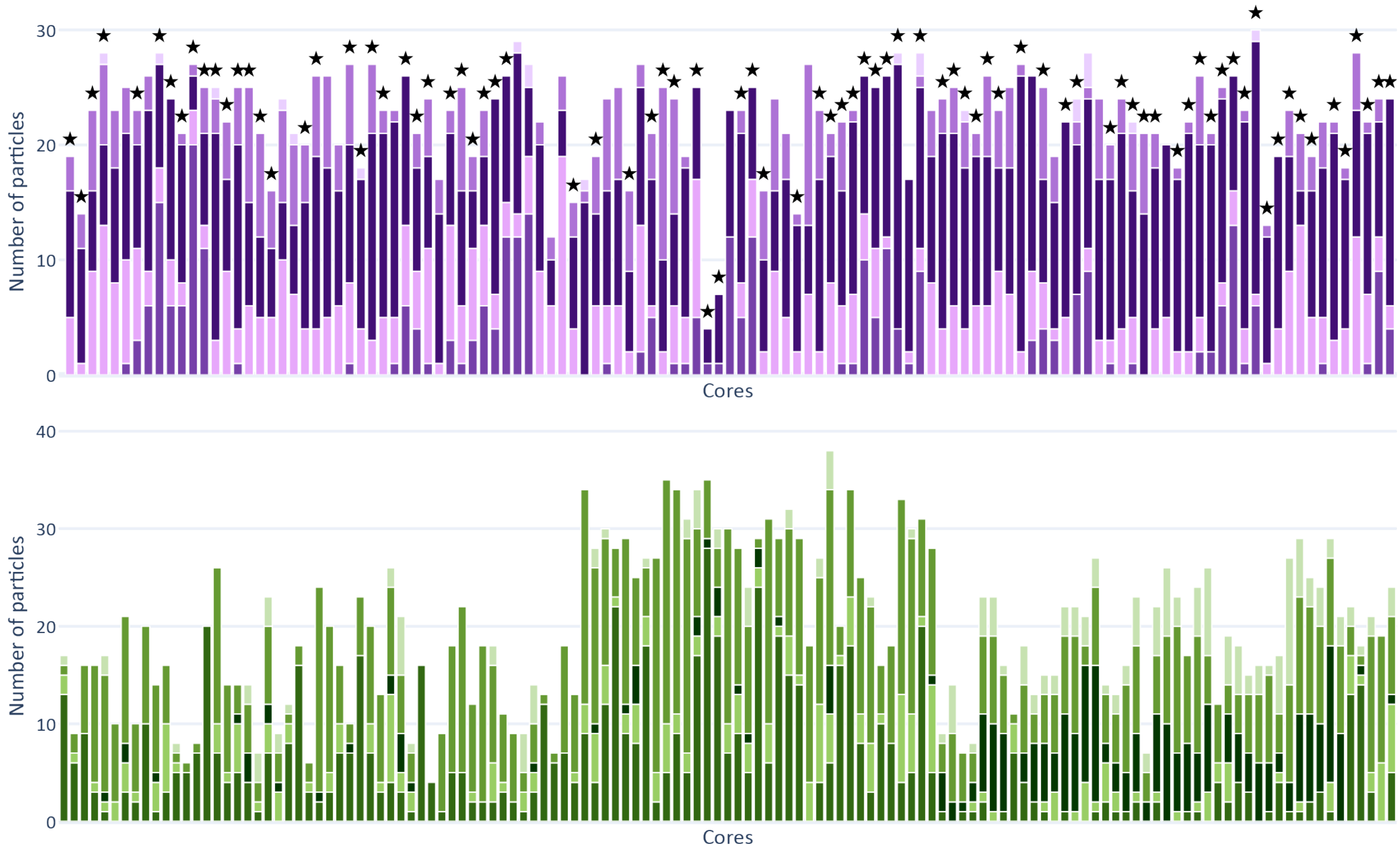

Supplementary Figure 20: Per-core class distribution of pore complexes. a. Distribution of pore complex classes per individual core under transcribing conditions. Each bar represents a single core, with colors corresponding to the classes defined in Fig. 4. The distribution shows that all classes are present across cores, without obvious enrichment patterns. The stars mark cores that are broken. Particle counts per class in intact and broken cores were 67/231 (class 1), 178/382 (class 2), 355/1,042 (class 3), 123/279 (class 4) and 9/12 (class 5), corresponding to 57–78% of particles on broken cores in every class, including the uncapped class 3 (75%). **b.** Same analysis as in (a), shown for untreated cores. A similar distribution is observed, with all classes represented across individual cores, consistent with a random distribution. Source data are provided as a Source Data file.

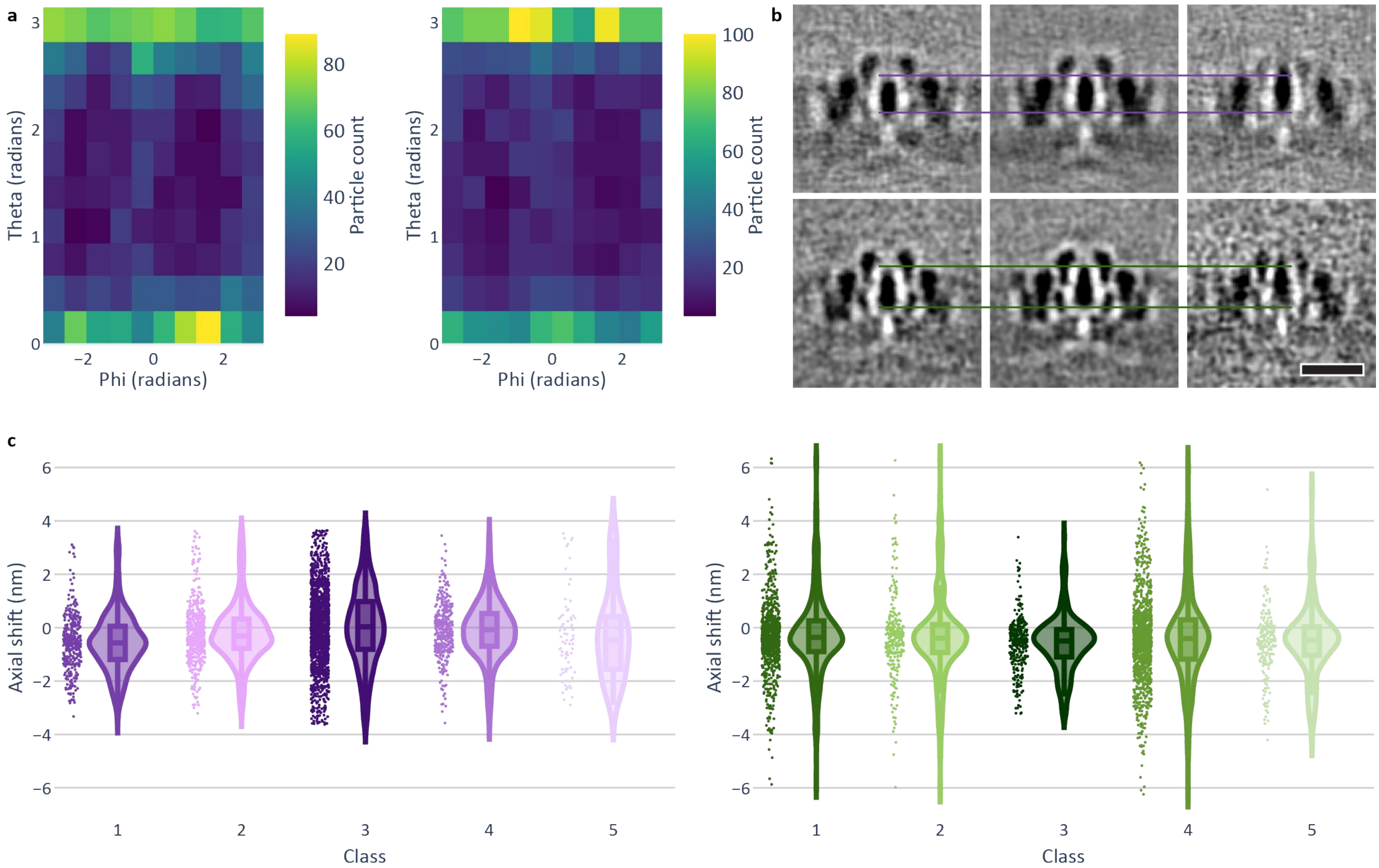

Supplementary Figure 21: Analysis of rod orientation and axial positional variability. a. Polar histograms of rod orientations derived from rod-focused alignment, shown for transcribing (left) and untreated (right) conditions. The distributions are strongly concentrated around θ ≈ 0° and 180°, indicating that the rod remains aligned with the pore axis and undergoes in-plane rotation without substantial tilting. **b.** Side-view slices of STA maps obtained by binning particles according to their axial shifts derived from rod-focused alignment. Top row: transcribing condition; bottom row: untreated condition. From left to right: particles with shifts ≤ −2 nm, shifts within ±0.5 nm, and shifts ≥ +2 nm. In all bins, a continuous rod density is observed, with small positional offsets along the pore axis as highlighted by the lines. **c.** Violin plots showing the distribution of axial shifts for individual pore classes under transcribing (left; violet) and untreated (right; green) conditions. The central line marks the median, the box the 25th–75th percentiles, and the whiskers extend to 1.5x the interquartile range, with all individual data points overlaid; n = 298 (class 1), 560 (2), 1,397 (3), 402 (4) and 21 (5) particles for the transcribing condition, and 738, 251, 283, 1,118 and 200 for the untreated condition. Each class exhibits a broad distribution of shifts, with substantial overlap between classes, indicating that axial rod displacement is not confined to specific structural classes. Source data are provided as a Source Data file. Scale bar 15 nm.

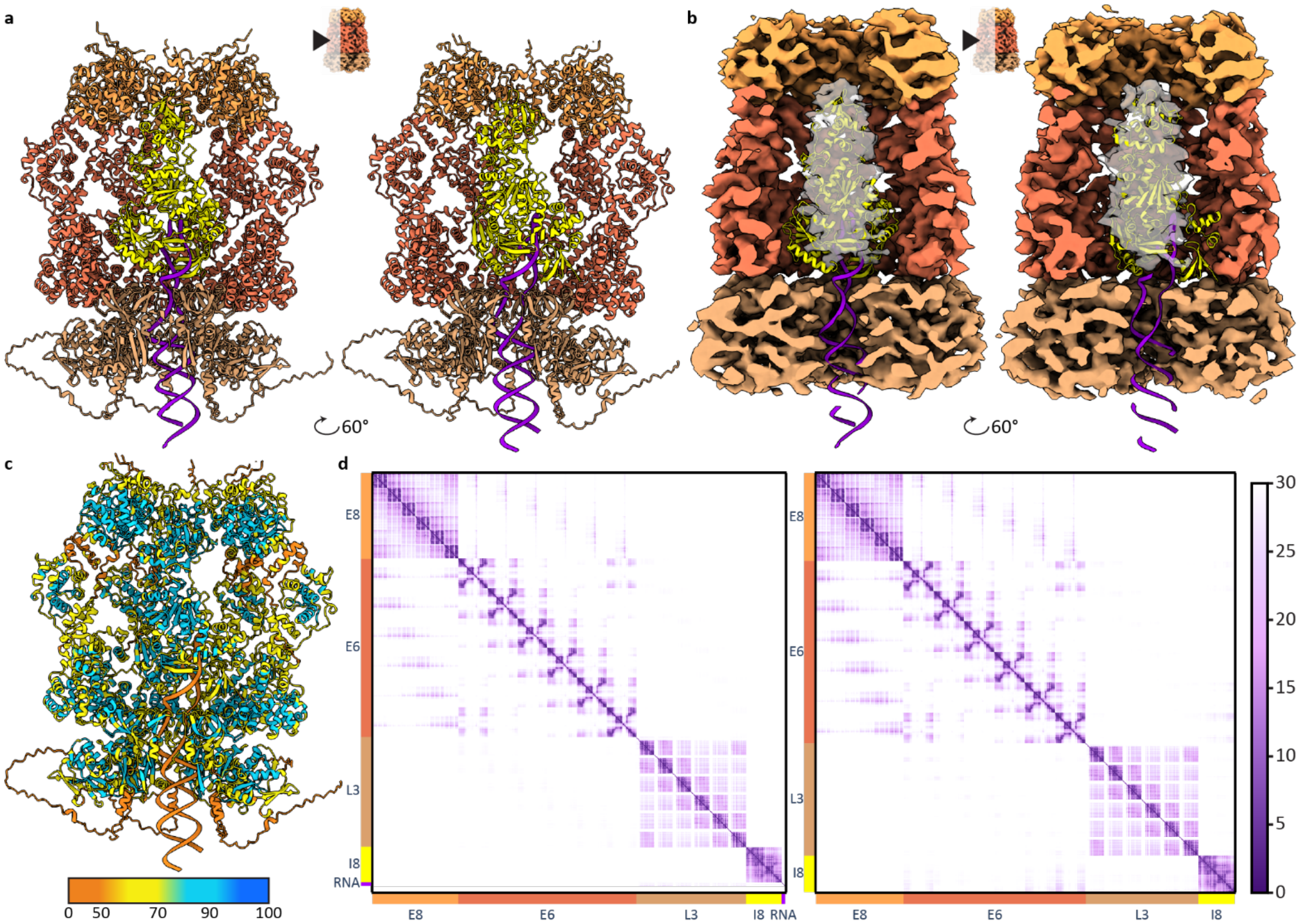

Supplementary Figure 22: AlphaFold3 prediction of the pore complex with RNA and helicase I8. **a.** AF3 prediction of the pore complex including the helicase I8 (yellow) and RNA (violet; ~70 nucleotides). The model is shown with the L3 hexamer (as the dodecamer did not converge reliably during prediction). The pore components are colored as follows: E8 hexamer (apricot), E6 hexamer (orange), and L3 hexamer (cappuccino). The right panel shows the model rotated by 60° to expose the wider region of the helicase. The prediction suggests RNA unwinding within the helicase region. As observed previously, the relative positioning of the hexameric assemblies is preserved, but the vertical spacing between rings is compressed compared to the experimental map. **b.** Superposition of the AF3-predicted RNA and I8 helicase onto the experimentally derived pore structure, with the same color coding as in (a). The model suggests that RNA can be accommodated within the L3 channel. **c.** Same model as in (a, right), colored by per-residue confidence (pLDDT), with values indicated on the scale bar. The ipTM is 0.41. **D.** Predicted Aligned Error (PAE) plots for the model shown in (a) (left) and for a corresponding prediction without RNA (right; see Fig. 6a), highlighting similarities in predicted confidence and inter-domain relationships.

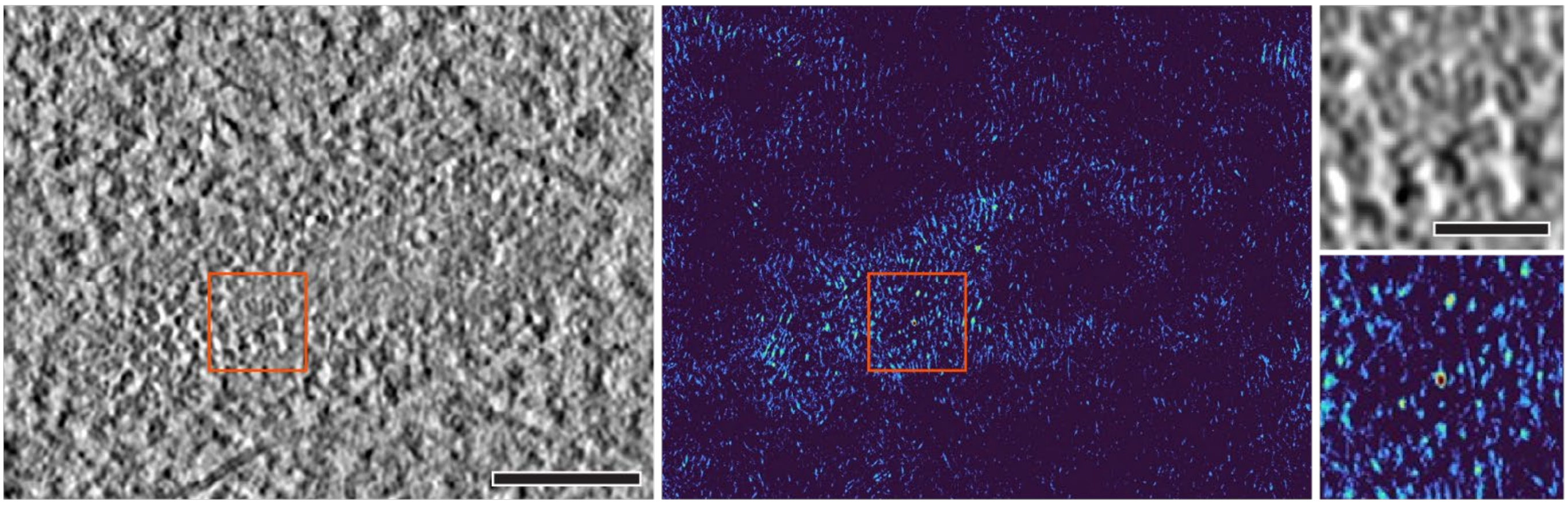

Supplementary Figure 23: Template matching on in situ tomograms. Left: Tomographic slice showing a pore complex in top view (highlighted). Middle: Corresponding cross-correlation (score) map obtained from 3D template matching using GAPSTOP(TM). Right: Zoomed-in views of the highlighted region, showing the tomogram slice (top) and the corresponding cross-correlation map (bottom), demonstrating the strong peak corresponding to the pore complex localization. Scale bars 100 nm, 30 nm.

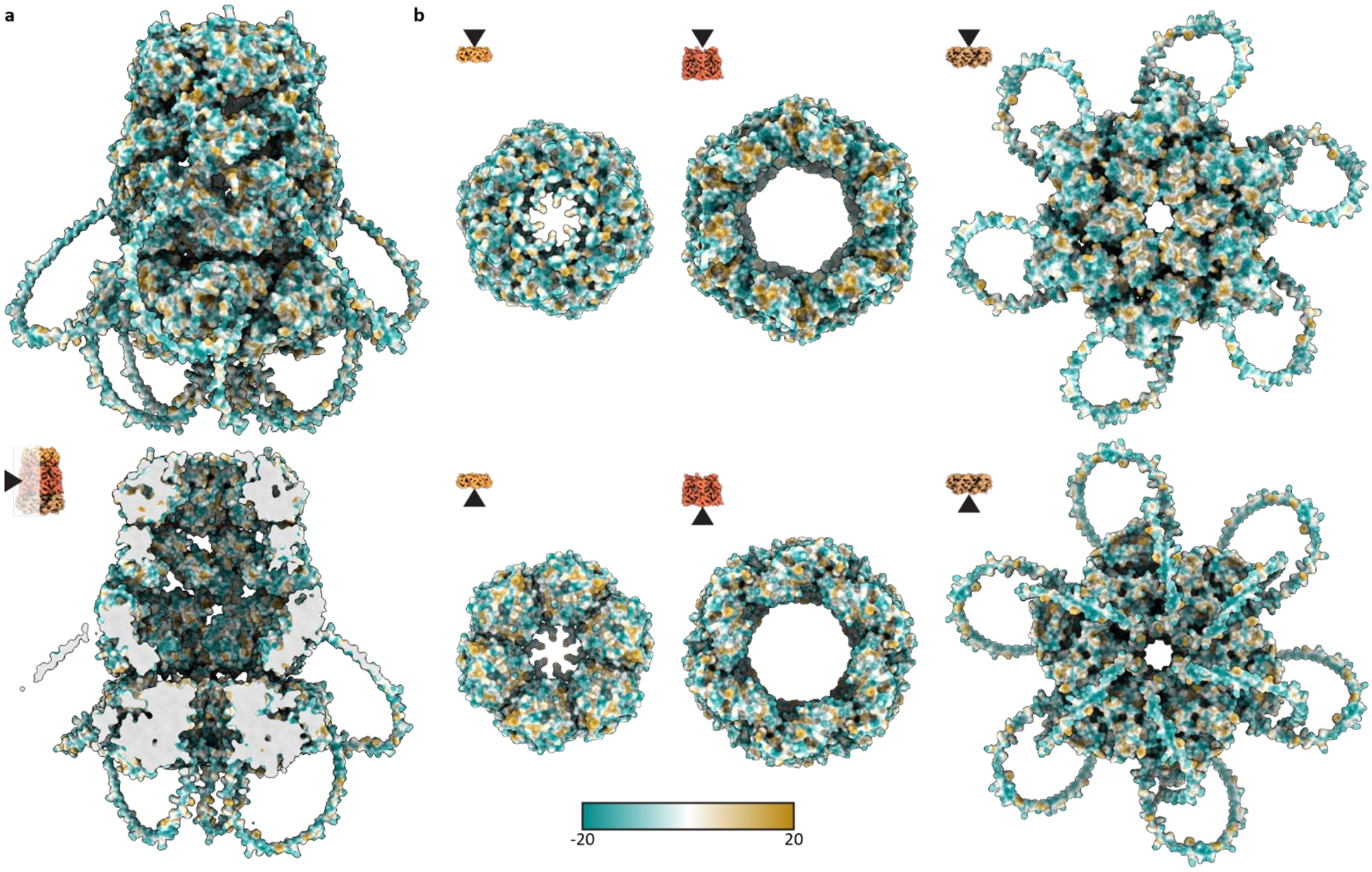

Supplementary Figure 24: Hydrophobic surface properties of the pore complex. a. Side view of the assembled pore complex model composed of the E8 hexamer, E6 hexamer, and L3 dodecamer. The top panel shows the intact structure, while the bottom panel displays a cut-open view to reveal the inner channel. The surface is colored according to hydrophobicity, with values indicated on the scale bar. The inner surface of the pore is predominantly hydrophilic (values around −20), whereas the tips of the E8 subunits extending into the channel display near-neutral values. **b.** Top (upper row) and bottom (lower row) views of the individual ring assemblies, shown in the order E8, E6, and L3. The E8 near-neutral tips extending into the channel are visible in the top view. In addition, the interface regions between the bottom of E8 and the top of E6 display increased hydrophobicity, suggesting a potential contribution of hydrophobic interactions to the stabilization of the ring–ring interface.

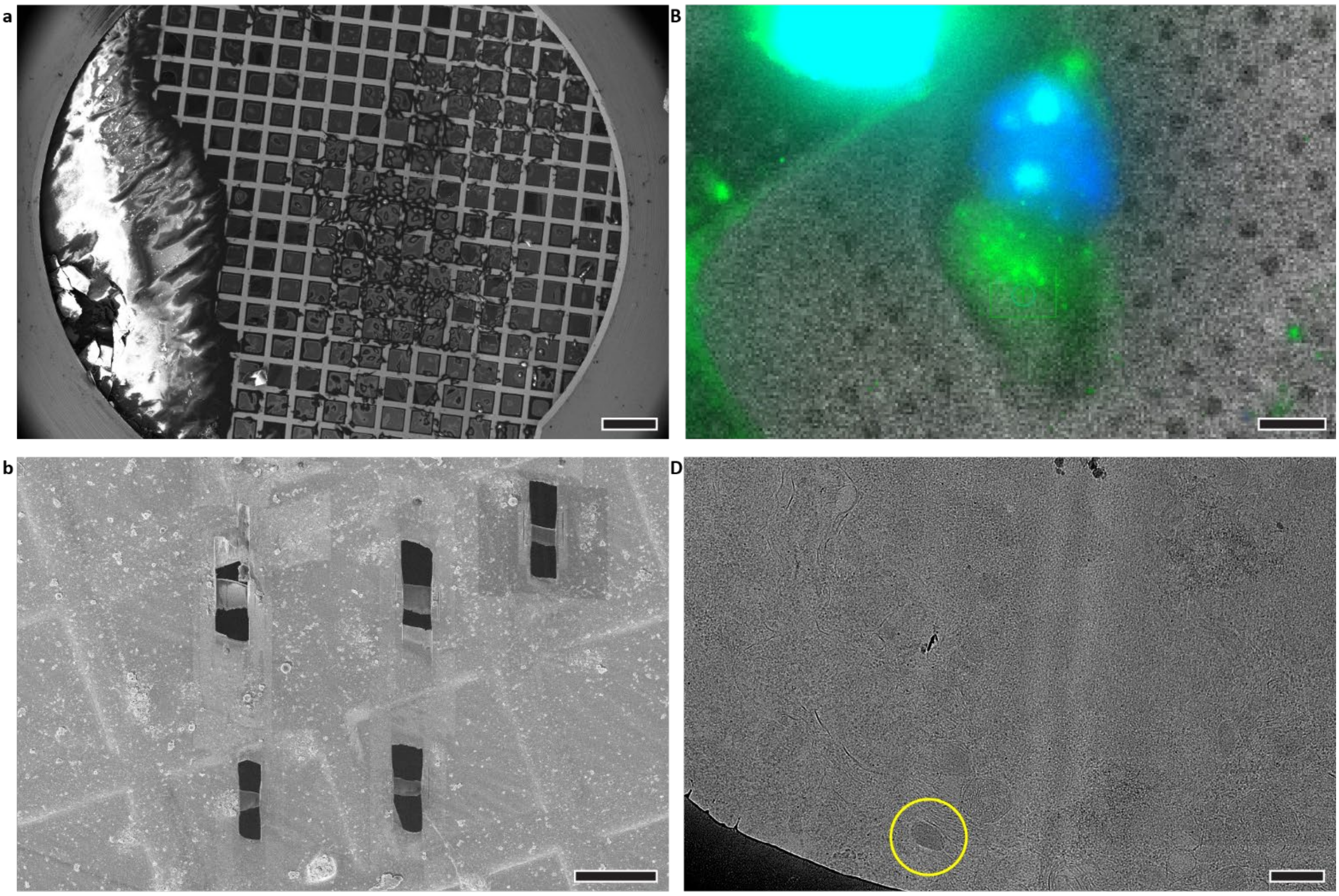

Supplementary Figure 25: Cryo-FIB workflow. a. Representative plunge-frozen grid containing VACV-infected HeLa cells. **b.** Cryo-fluorescence microscopy of a HeLa cell infected with YFP-labeled VACV and stained with Hoechst 33342. Individual viral particles are clearly visible and well distributed throughout the cell. **c.** High-throughput focused ion beam (FIB) milling. Most grid squares contain suitable targets for the preparation of thin lamellae. **d.** Medium-magnification cryo-TEM image of a lamella. Cellular ultrastructure is well preserved, and a viral core is visible (circled). Scale bars: 200 µm (A), 10 µm (B), 50 µm (C), and 500 nm (D).

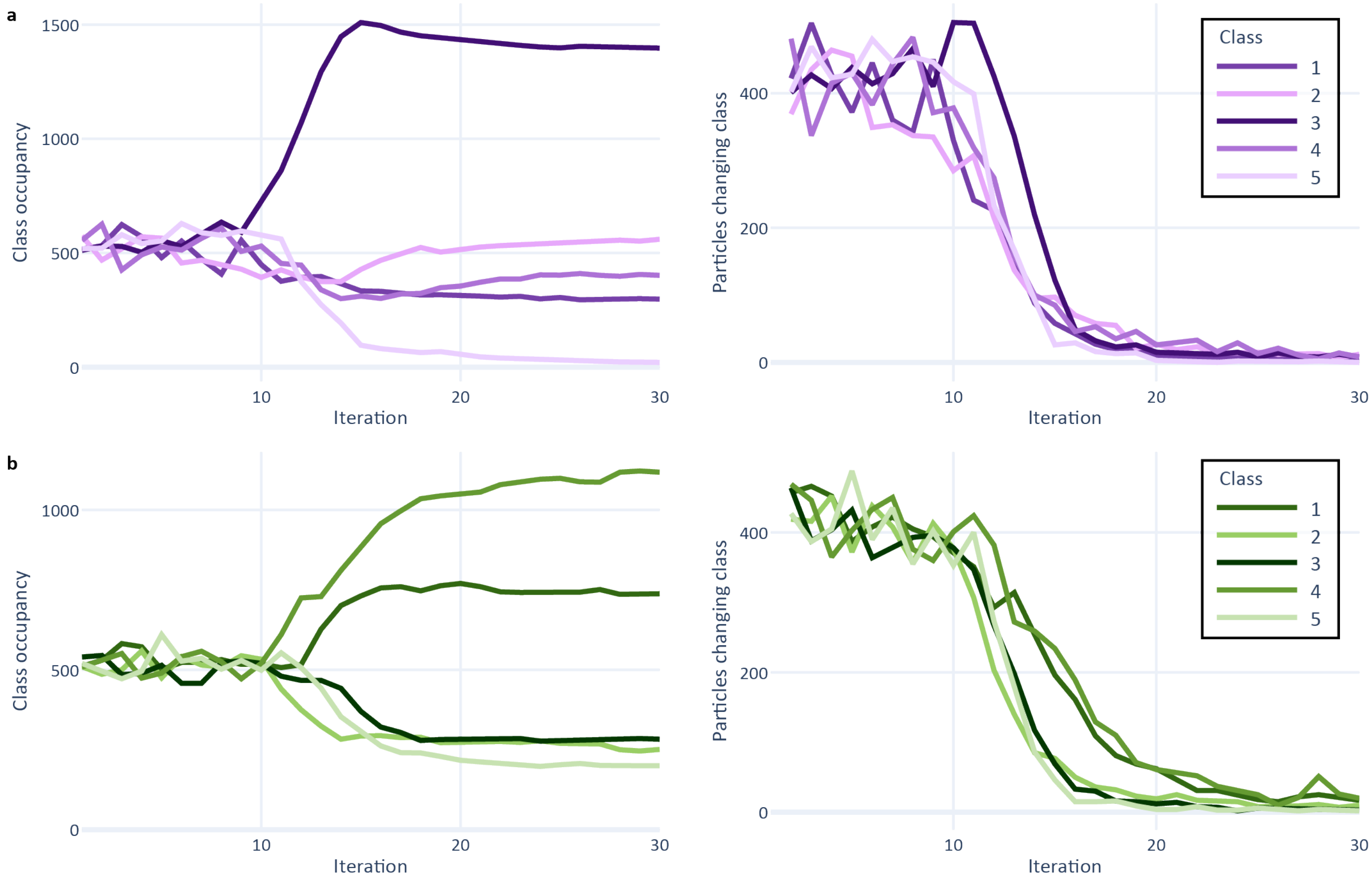

Supplementary Figure 26: Classification convergence analysis. a. Classification results for the transcribing condition. Left: class occupancy across iterations, showing the number of particles assigned to each class per iteration. Right: number of particles changing class assignment between successive iterations. The class occupancies stabilize by approximately iteration 20, accompanied by a plateau in class changes, indicating convergence of the classification. Classes 1-5 comprise 298, 560, 1,397, 402 and 21 particles, respectively. **b.** Same analysis as in (a) for the untreated condition. Class occupancies similarly stabilize around iteration 20, while the number of class changes decreases more gradually and reaches a plateau around iteration 25. Classes 1-5 comprise 738, 251, 283, 1,118 and 200 particles, respectively. Source data are provided as a Source Data file.

SUPPLEMENTARY TABLES

Supplementary Table 1: Interactors with A10 revealed by XL-MS. Proteins cross-linked to A10, with their gene name, UniProt identifier, molecular mass, the number of cross-links detected with A10, the oligomeric states tested by AF3 prediction, and available information on their localization and function with the corresponding references. Molecular mass is reported for completeness and was not used as an exclusion criterion.

Protein	Gene	UniProtID	MW (kDa)	#XLs with A10	Oligomeric states predicted with AF3	Available information on localization/function	References in PubMed (PMID)
**A3**	OPG129	P06440	72	79	1-12	Core protein (putative core-wall), important for morphogenesis	38316877, 4777308
**A4**	OPG130	P29191	31	26	1-12	Core/membrane protein; available PDB (8R5I) is mixture of A10 trimer and A4 monomer	8794334, 10600608
**A12**	OPG138	Q80HV7	20	4	1-12	Core protein	17625005
**A27**	OPG154	P11258	12	3	Structure known	Membrane/envelope protein; forms hexamer; PDBs: 3VO5, 5EOQ, 5EOR	15913650
**Actin ACM1***	ACTC1	P68032	42	1	x	Host protein	x
**D8**	OPG105	P04195	35	4	1-12	Membrane/envelope protein; should bind to the cell surface	2104847, 3418784
**E6**	OPG068	P21607	67	5	1-12	Core protein; required for virion assembly	20116821, 19217136, 38171004
**E8**	OPG070	P23372	32	1	1-12	Core-protein; essential for transcription	17619043
**F17**	OPG062	P07396	11	22	1-12	Phosphoprotein; lateral bodies	10729126, 1920628
**G7**	VACWR085	P68716	42	4	1-12	Should be part of a complex composed of A30, G7, F10 kinase, A15, D2, D3, and J1	15567438
**H1**	OPG106	P07239	20	9	Structure known	Dual-specificity phosphatase; forms dimer; PDBs: 2Q05, 2RF6, 3CM3	1848923, 21362620, 10438817
**L3**	OPG097	P07614	41	5	1-12	Factor for early transcription	16282472
**L4**	OPG098	P03295	28	1	1-12	Single stranded DNA/RNA binding, core transcription	15163727, 9321647, 8551633

*ACM1: alpha cardiac muscle 1

Supplementary Table 2: Results of AF3 predictions for chosen interactors of A10. P: protein; OS: oligomeric state; MW: molecular weight in kDa; ipTM: interface predicted template modeling score; pTM: predicted template modeling score; RS: ranking score; C: clash; FD: fraction disordered.

P	OS	MW	ipTM	pTM	RS	C	FD
**A3**	1	72		0.8	0.82	0	0.04
**A3**	2	144	0.22	0.48	0.3	0	0.06
**A3**	3	216	0.35	0.49	0.4	0	0.04
**A3**	4	288	0.58	0.64	0.61	0	0.03
**A3**	5	360	0.52	0.57	0.55	0	0.05
**A3**	6	432	0.39	0.46	0.42	0	0.03
**A3**	7	504	0.38	0.44	0.41	0	0.03
**A3**	8	576	0.3	0.37	0.33	0	0.04
**A3**	9	648	0.32	0.37	0.34	0	0.02
**A3**	10	720	0.36	0.4	-99.62	1	0.03
**A3**	11	792	0.38	0.42	0.41	0	0.04
**A3**	12	864	0.38	0.42	0.41	0	0.04
**A4**	1	31		0.24	0.69	0	0.9
**A4**	2	62	0.1	0.15	0.5	0	0.79
**A4**	3	93	0.16	0.19	0.53	0	0.72
**A4**	4	124	0.17	0.19	0.42	0	0.49
**A4**	5	155	0.17	0.19	0.53	0	0.69
**A4**	6	186	0.16	0.17	0.44	0	0.56
**A4**	7	217	0.17	0.19	0.4	0	0.45
**A4**	8	248	0.19	0.2	0.34	0	0.3
**A4**	9	279	0.21	0.21	0.37	0	0.32
**A4**	10	310	0.21	0.22	0.37	0	0.32
**A4**	11	341	0.22	0.22	0.36	0	0.29
**A4**	12	372	0.22	0.23	0.29	0	0.12
**A12**	1	20		0.23	0.66	0	0.85
**A12**	2	40	0.09	0.16	0.5	0	0.8
**A12**	3	60	0.21	0.22	0.58	0	0.74
**A12**	4	80	0.14	0.17	0.52	0	0.75
**A12**	5	100	0.15	0.17	0.55	0	0.8
**A12**	6	120	0.16	0.18	0.54	0	0.76
**A12**	7	140	0.17	0.19	0.56	0	0.76
**A12**	8	160	0.18	0.19	0.46	0	0.55
**A12**	9	180	0.17	0.19	0.49	0	0.63
**A12**	10	200	0.18	0.2	0.5	0	0.62
**A12**	11	220	0.18	0.2	0.38	0	0.39
**A12**	12	240	0.2	0.21	0.44	0	0.47
**D8**	1	35		0.78	0.83	0	0.1
**D8**	2	70	0.16	0.46	0.22	0	0
**D8**	3	105	0.17	0.37	0.21	0	0
**D8**	4	140	0.15	0.31	0.18	0	0
**D8**	5	175	0.17	0.28	0.19	0	0
**D8**	6	210	0.18	0.29	0.2	0	0
**D8**	7	245	0.19	0.27	0.23	0	0.04
**D8**	8	280	0.21	0.27	0.22	0	0
**D8**	9	315	0.22	0.28	0.23	0	0
**D8**	10	350	0.23	0.28	0.24	0	0
**D8**	11	385	0.24	0.28	0.25	0	0
**D8**	12	420	0.24	0.29	0.25	0	0
**E6**	1	67		0.73	0.73	0	0
**E6**	2	134	0.13	0.41	0.18	0	0
**E6**	3	201	0.2	0.36	0.23	0	0
**E6**	4	268	0.44	0.51	0.45	0	0
**E6**	5	335	0.5	0.54	0.51	0	0
**E6**	6	402	0.4	0.45	0.41	0	0
**E6**	7	469	0.28	0.35	0.3	0	0
**E6**	8	536	0.32	0.36	-99.67	1	0
**E6**	9	603	0.33	0.37	-99.66	1	0
**E6**	10	670	0.34	0.37	-99.66	1	0
**E6**	11	737	0.34	0.37	-99.65	1	0
**E6**	12	804	0.35	0.38	-99.64	1	0
**E8**	1	32		0.88	0.9	0	0.05
**E8**	2	64	0.67	0.78	0.71	0	0.04
**E8**	3	96	0.63	0.74	0.67	0	0.04
**E8**	4	128	0.62	0.7	0.66	0	0.04
**E8**	5	160	0.91	0.92	0.93	0	0.04
**E8**	6	192	0.87	0.89	0.9	0	0.04
**E8**	7	224	0.61	0.66	0.64	0	0.04
**E8**	8	256	0.37	0.45	0.41	0	0.04
**E8**	9	288	0.4	0.47	0.44	0	0.04
**E8**	10	320	0.43	0.48	0.46	0	0.04
**E8**	11	352	0.35	0.41	0.38	0	0.04
**E8**	12	384	0.35	0.4	0.38	0	0.04
**F17**	1	11		0.25	0.75	0	1
**F17**	2	22	0.3	0.31	0.78	0	0.95
**F17**	3	33	0.14	0.18	0.65	0	1
**F17**	4	44	0.31	0.32	0.81	0	1
**F17**	5	55	0.34	0.35	0.84	0	1
**F17**	6	66	0.37	0.37	0.87	0	1
**F17**	7	77	0.35	0.35	0.85	0	1
**F17**	8	88	0.4	0.4	0.9	0	1
**F17**	9	99	0.41	0.42	0.91	0	1
**F17**	10	110	0.43	0.43	0.93	0	1
**F17**	11	121	0.39	0.4	0.89	0	1
**F17**	12	132	0.36	0.37	0.86	0	1
**G7**	1	42		0.61	0.74	0	0.26
**G7**	2	84	0.28	0.31	0.32	0	0.07
**G7**	3	126	0.2	0.3	0.28	0	0.11
**G7**	4	168	0.28	0.38	0.34	0	0.08
**G7**	5	210	0.4	0.46	0.45	0	0.08
**G7**	6	252	0.35	0.41	0.41	0	0.09
**G7**	7	294	0.3	0.36	0.38	0	0.13
**G7**	8	336	0.27	0.32	-99.65	1	0.15
**G7**	9	378	0.27	0.32	-99.67	1	0.1
**G7**	10	420	0.24	0.27	0.27	0	0.05
**G7**	11	462	0.27	0.31	-99.65	1	0.15
**G7**	12	504	0.28	0.31	0.29	0	0
**L3**	1	41		0.76	0.85	0	0.18
**L3**	2	82	0.42	0.59	0.54	0	0.17
**L3**	3	123	0.55	0.63	0.65	0	0.16
**L3**	4	164	0.38	0.48	0.48	0	0.17
**L3**	5	205	0.63	0.67	0.72	0	0.17
**L3**	6	246	0.73	0.74	0.82	0	0.17
**L3**	7	287	0.7	0.72	0.78	0	0.17
**L3**	8	328	0.31	0.37	0.41	0	0.17
**L3**	9	369	0.37	0.42	0.47	0	0.18
**L3**	10	410	0.3	0.35	0.4	0	0.17
**L3**	11	451	0.32	0.36	0.41	0	0.17
**L3**	12	492	0.32	0.36	0.42	0	0.18
**L4**	1	28		0.82	0.87	0	0.1
**L4**	2	56	0.14	0.45	0.24	0	0.07
**L4**	3	84	0.14	0.35	0.24	0	0.12
**L4**	4	112	0.16	0.32	0.23	0	0.08
**L4**	5	140	0.16	0.29	0.22	0	0.07
**L4**	6	168	0.18	0.29	0.22	0	0.04
**L4**	7	196	0.18	0.27	0.21	0	0.03
**L4**	8	224	0.19	0.26	0.26	0	0.11
**L4**	9	252	0.2	0.27	0.27	0	0.11
**L4**	10	280	0.2	0.27	0.24	0	0.05
**L4**	11	308	0.21	0.27	0.22	0	0
**L4**	12	336	0.22	0.27	0.28	0	0.1

Supplementary Table 3: Symmetry expansion of E6, E8 and L3 protomers fitted to the pore density. A single protomer of E6, E8 or L3 was expanded under C_n symmetry (n = 2-12) about the pore axis and fitted to the corresponding region of the STA density; for L3, the inner and outer rings of the basal assembly were treated separately. Two values are given per symmetry: Assembly, the cross-correlation about the mean between the complete C_n assembly and the density; and Monomer, the same correlation computed for each copy individually, as mean ± s.d. over the n copies. Fitting was performed in ChimeraX with the central rod density masked out (see Methods). Only expansions commensurate with six-fold symmetry (C2, C3, C6; bold) place all copies into density, giving high correlation with low variance; other symmetries position subunits away from the density and fit worse, with higher per-copy variance as copies scatter. Negative monomer correlations (e.g. L3 outer, C8) arise at incommensurate symmetries when expanded copies fall between the true lobe positions, placing density where the map is below its mean; the correspondingly large variance reflects the incoherent scatter expected when the symmetry does not match the six-fold arrangement. The inner and outer L3 rings show the same pattern independently.

	**E8**		**E6**		**L3 inner ring**		**L3 outer ring**	
	Assembly	Monomer	Assembly	Monomer	Assembly	Monomer	Assembly	Monomer
**C2**	0.36	0.30 ± 0.03	0.41	0.35 ± 0.07	0.30	0.24 ± 0.08	0.37	0.32 ± 0.02
**C3**	0.32	0.29 ± 0.03	0.39	0.35 ± 0.03	0.30	0.25 ± 0.05	0.38	0.32 ± 0.03
**C4**	0.23	0.20 ± 0.13	0.22	0.19 ± 0.19	0.17	0.14 ± 0.12	0.17	0.15 ± 0.20
**C5**	0.15	0.10 ± 0.13	0.14	0.10 ± 0.16	0.20	0.09 ± 0.12	0.12	0.06 ± 0.15
**C6**	0.34	0.30 ± 0.04	0.39	0.35 ± 0.04	0.31	0.24 ± 0.04	0.38	0.32 ± 0.03
**C7**	0.13	0.11 ± 0.10	0.14	0.10 ± 0.13	0.10	0.07 ± 0.10	0.13	0.05 ± 0.13
**C8**	0.14	0.12 ± 0.12	0.16	0.11 ± 0.15	0.14	0.09 ± 0.10	0.10	-0.47 ± 1.57
**C9**	0.15	0.14 ± 0.12	0.20	0.15 ± 0.16	0.15	0.10 ± 0.12	0.14	0.10 ± 0.17
**C10**	0.11	0.10 ± 0.11	0.16	0.10 ± 0.13	0.17	0.07 ± 0.10	0.09	0.06 ± 0.14
**C11**	0.20	0.11 ± 0.09	0.20	0.11 ± 0.12	0.20	0.09 ± 0.10	0.11	0.04 ± 0.12
**C12**	0.26	0.20 ± 0.11	0.32	0.19 ± 0.17	0.24	0.14 ± 0.12	0.23	0.14 ± 0.19

Supplementary Table 4: Conformational clustering of E6 hexamer AF3 predictions. The 500 predictions were grouped by conformation (agglomerative clustering, Ward linkage). For each cluster, the number of models and the mean of each descriptor are given: Cα RMSD to the fitted E6 model (six-fold-aware), channel radius (closest approach of any Cα to the symmetry axis), axial span, and AF3 ipTM. All clusters are well above the closed state (RMSD ≥ 17.6 Å) at uniformly low ipTM, and none approaches the fitted model.

Cluster	Models	Mean Cα RMSD to fitted model (Å)	Mean channel radius (Å)	Mean axial span (Å)	Mean ipTM
**1**	143	21.82	33.89	96.24	0.401
**2**	36	17.61	33.73	101.63	0.393
**3**	202	25.68	33.9	91.25	0.405
**4**	72	26.22	33.87	91.01	0.406
**5**	47	29.72	33.8	84.62	0.406

Supplementary Table 5: Fit of predicted and fitted pore models to the STA density. Cross-correlation (CC), cross-correlation about the mean (CAM), occupancy and model overflow for the E6 hexamer and for the complete pore complex, each evaluated as an AF3 prediction, an AF3x cross-link-guided prediction, and the final fitted model of this study. For the E6 hexamer, values correspond to the E6 component extracted from each corresponding full model rather than to independent E6 predictions. All models were fitted into the STA density by global search in ChimeraX at a permissive contour level with the central rod density masked out (see Methods); the values quoted in the main text are the CC column. Occupancy is the fraction of the pore density covered by the model, and overflow is the fraction of the model volume lying outside the density. For both the isolated E6 hexamer and the full complex, the fit improves from the AF3 prediction through the AF3x model to the final fitted model, as the initially open E6 hexamer closes and the model is brought into agreement with the observed inter-ring spacing; the decreasing overflow reflects the progressive removal of density that initially extended beyond the map. For the full complex, the final model including the outer L3 ring is listed together with the same model without the outer ring: the outer ring increases the fraction of density covered (higher occupancy) at a slightly lower global correlation, as it is the least-constrained, density-guided component. Its higher overflow reflects the unstructured L3 N-terminus (residues 1-65), which forms extended, tentacle-like features projecting beyond the ordered density.

Model	CC	CAM	Occupancy	Overflow
**Full pore model**				
AF3	0.37	0.13	0.15	0.48
AF3x	0.51	0.24	0.19	0.35
Ours without the L3 outer ring	0.63	0.39	0.21	0.22
Ours	0.59	0.37	0.26	0.26
**E6 hexamer only**				
AF3	0.48	0.20	0.08	0.41
AF3x	0.63	0.35	0.10	0.25
Ours	0.68	0.41	0.11	0.19

Supplementary Table 6. Orthogonal evidence for pore-complex interfaces. Contacts were built from the AlphaFold3 model and fitted into the STA density; as side chains are not resolved at this resolution, modelled contacts are reported as consistent with the density rather than directly observed, and interface existence and location are assessed against sequence conservation and DSSO cross-links. ConSurf grades (1–9, 9 = most conserved) were computed on the monomers, independently of the model, density or oligomeric state; where a low grade reflects substitutions preserving physicochemical character, the conservation of that character is given alongside. Distances are Cα–Cα in the fitted model (DSSO limit ~35 Å): for long intra-chain links the distance within a single protomer exceeds this limit and therefore requires an inter-protomer arrangement, whereas same-residue links are inter-protomer by definition and do not depend on the modelled distance. Support level: Strong = corroborated by conservation and/or model-independent cross-links; Partial = conservation only.

Chains	Modelled contact	Conservation (ConSurf grade 1-9)	Supporting XL-MS restraint(s)	Ca-Ca distance (A)	Support level
**E8 : E8**	Polar interaction network centred on Ser56/Ser57 engaging Asp178 and Thr24	Thr24 (9); Ser57 (9); Asp178 (9); Ser56 (7); Thr176 (4)	Long intra-chain E8 links exceeding 35 A within one protomer, therefore inter-protomer: 13-267, 13-161, 12-268	49.3 / 37.8 / 48.9	Conservation + inter-protomer XL
**E6 : E8**	Acidic E6 helix 341-356 facing two E8 protomers; electrostatic complementarity,	E6 acidic face conserved in charge: Asp342 (8) 93% acidic; Glu341 (5) 88%; Asp351 (6) 83%; Glu353 (5) 80% (grades reflect Asp/Glu interconversion). E8 C-terminal region: Lys262 (7) 100% basic; Lys251 (7) 88% basic	None - no E8-E6 cross-link detected	-	Conserved charge complementarity; no XL
**E6 : E6 (domain 2)**	Electrostatic cluster Asp554 with Arg5/Arg6 (N-term to C-term)	Arg5 (9); Arg6 (9); Asp554 (7)	Long intra-chain E6 links exceeding 35 A within one protomer, therefore inter-protomer: 372-443, 372-445, 372-446, 478-552, 456-556	43.5 / 48.7 / 50.1 / 54.1 / 61.1	Conservation + inter-protomer XL)
**E6 : L3.2**	Hydrophobic packing involving conserved nonpolar residues	E6 Phe82 (8); L3 Ile308 (7); E6 Ala81 (5)	None direct (only E6-L3 link 553-14 is 85 A; flexible terminus)	-	Partial (conservation)
**L3.1 : L3.1**	Three-stranded beta-sheet: hairpin (strands 322-329, 338-341) from one protomer augmented by a strand from its neighbour	Buried core: Leu323 (9); Phe328 (9); Ile325 (8); Val322 (7). Solvent face: Asp319 (9); Glu324 (9); Asp327 (9); Glu326 (8); Glu338 (8). Low-grade positions conserved in character: Phe340 (4) 100% hydrophobic; Met339 (5) 97% hydrophobic	Same-residue link Lys272-Lys272 (inter-protomer by definition; short distance compatible with the compact inner ring)	14.8	Strong (conservation both faces + same-residue XL)
**L3 : L3 (N-terminal region)**	N-terminal segment (residues ~14-55), unresolved in the density, engaged in inter-protomer contacts	Acceptor region conserved: Thr260 (8); Lys261 (7); Lys268 (7); Lys272 (7)	Same-residue links, inter-protomer by definition: 14-14, 22-22, 55-55, 272-272	47.5 / 56.1 / 30.8 / 14.8 (modelled; not used for the inference)	Strong (model-independent XL)
**E6 : A10**	E6 protomer contacts three A10 trimers	Lys439 (4); Lys505 (4); Lys478 (2); Lys552 (2)	E6-A10 links: 478-366, 478-370, 439-162, 552-34	16.8 / 20.1 / 19.4 / 25.2	Strong (XL)

Supplementary Table 7: Response of the predicted L3 hexamer to targeted substitutions. All values are Cα RMSD (Å) relative to the wild-type prediction, calculated as described in Methods. Per-protomer values are mean ±s.d. over the six protomers; regional values (N-terminal, C-terminal) are derived from the mean per-residue deviation profile and are therefore given without s.d. Residues 1-65 were excluded throughout. The control-loop value for Proline strand (2.34 Å) arises from deviation of residues 147-152 in that variant and is unrelated to the substitutions made in the Proline loop construct. Residues 80-83 deviate by 6.5-7.2 Å in all three variants, including the two in which the assembly is reproduced, and are therefore variable between predictions independently of the substitutions. Per-protomer and per-residue values are provided in the Source Data file.

Variant	Substituted residues (number of substitutions is 12 for all)	Whole-ring Cα RMSD (Å)	Protomer body (Å)	Strand region 315-345 (Å)	Substituted strands 322-329/338-341 (Å)	Control loops 137-140/147-154 (Å)	N-term region 66-260 (Å)	C-term region 261-345 (Å)	Maximum deviation at own substituted sites (Å)
**Proline strand**	322-329, 338-341	26.4	2.52 ± 0.01	6.67 ± 0.04	6.99 ± 0.04	2.34 ± 0.05	1.46	5.49	10.5
**Valine Strand**	322-329, 338-341	1.05	0.85 ± 0.01	1.04 ± 0.02	0.99 ± 0.03	0.45 ± 0.03	0.82	0.96	1.8
**Proline loop**	137-140, 147-154	1.11	0.87 ± 0.02	1.21 ± 0.04	0.71 ± 0.07	2.80 ± 0.03	1.14	0.88	5.3

Supplementary Table 8: AF3 predictions of I8 across oligomeric states. I8 was predicted from monomer to dodecamer, and the resulting models assessed by AF3 confidence metrics: the interface predicted TM-score (ipTM, not defined for the monomer), the predicted TM-score (pTM), and the ranking score, together with the fraction of clashing atoms and the fraction of the model predicted to be disordered. The monomer is predicted with high confidence (pTM 0.92), whereas all higher oligomeric states show low inter-chain confidence (ipTM 0.19–0.34), indicating that AF3 does not support the formation of a defined I8 oligomer.

Oligomeric state	MW (kDa)	ipTM	pTM	Ranking Score	Clash	Fraction disordered
**1**	77		0.92	0.92	0	0
**2**	154	0.19	0.51	0.25	0	0
**3**	231	0.21	0.43	0.25	0	0
**4**	308	0.22	0.38	0.26	0	0
**5**	385	0.24	0.36	0.26	0	0
**6**	462	0.26	0.35	0.28	0	0
**7**	539	0.27	0.35	0.29	0	0
**8**	616	0.29	0.36	0.31	0	0
**9**	693	0.31	0.36	0.32	0	0
**10**	770	0.33	0.37	0.33	0	0
**11**	847	0.34	0.38	0.35	0	0
**12**	924	0.35	0.39	0.36	0	0

Supplementary Table 9: Reference generation and template-matching (TM) parameters. Parameters for STA and TM steps used to generate the pore reference and detect particles. Two independent STAs were performed on the manually picked particles, with C1 and with C6 symmetry; the C6 average was upscaled to voxel size 6.25 Å, followed by 1 iteration of refinement, and used as the initial reference for TM on 7 tomograms. Positions and orientations from this TM were refined over four iterations with C6 symmetry, and the resulting reference was used for the final TM on all tomograms. The steps in the first four columns were performed on the untreated dataset (EMPIAR-11674), in which the pore was first observed. Entries marked "x" are not applicable.

STA parameters	STA on manually picked particles	STA for upscaling	TM on 7 tomograms	STA on TM from 7 tomograms	Final TM
**Voxel size (Å)**	12.48	6.24	6.24	6.24	6.24 & 5.572
**Box size (voxels)**	48	72	72	72	72
**Iterations**	10	1	x	4	x
**Cone angle (degrees)**	48	48	360	48	360
**Cone angle sampling**	4	4	10	4	10
**In-plane angle (degrees)**	120	120	360	120	360
**In-plane angle sampling**	10	10	10	10	10
**Low-pass filter (voxels)**	14	20	20	20	20
**High-pass filter (voxels)**	1	1	1	1	1
**Symmetry applied**	C1 and C6	C6	C1	C6	C1
**Alignment mask – type**	Cylindrical	Cylindrical	Cylindrical	Cylindrical	Cylindrical
**Alignment mask – radius (voxels)**	12	24	24	24	24
**Alignment mask – height (voxels)**	12	24	24	34	34
**Alignment mask – Gaussian**	2	2	2	2	2
**CC mask – type**	Cylindrical	Cylindrical	x	Cylindrical	x
**CC mask – radius (voxels)**	3	6	x	6	x
**CC mask – height (voxels)**	12	24	x	24	x
**Particle number**	32	32	x	157	x
**Software**	novaSTA	novaSTA	GAPSTOP(TM)	novaSTA	GAPSTOP(TM)

Supplementary Table 10: Confirmation of six-fold symmetry by in-plane angle randomization. The particle lists from the final TM (Supplementary Table 9, Supplementary Fig. 3) were used to test whether the six-fold symmetry is data-derived. The in-plane angle was randomized, yielding a featureless tube, followed by C1 STA with a large mask partially including the surrounding A10 trimers and with a tight mask focused on the pore.

STA parameters	Transcribing condition with A10 trimers	Transcribing condition pore only	Untreated condition with A10 trimers	Untreated condition pore only
**Voxel size (Å)**	5.572	5.572	6.24	6.24
**Box size (voxels)**	72	72	72	72
**Iterations**	10	10	10	10
**Cone angle (degrees)**	12	12	12	12
**Cone angle sampling**	4	4	4	4
**In-plane angle (degrees)**	360 360 180 180 80 80 40 40 40 40	360 360 180 180 80 80 40 40 40	360 360 180 180 80 80 40 40 40	360 360 180 180 80 80 40 40 40
**In-plane angle sampling**	20 15 10 10 8 8 5 5 5 5	20 15 10 10 8 8 5 5 5 5	20 15 10 10 8 8 5 5 5 5	20 15 10 10 8 8 5 5 5 5
**Low-pass filter (voxels)**	12 12 12 12 16 16 16 16 20 20	12 12 12 12 16 16 16 16 20 20	12 12 12 12 16 16 16 16 20 20	12 12 12 12 16 16 16 16 20 20
**High-pass filter (voxels)**	1	1	1	1
**Symmetry applied**	C1	C1	C1	C1
**Alignment mask – type**	Cylindrical	Cylindrical	Cylindrical	Cylindrical
**Alignment mask – radius (voxels)**	26	13	24	13
**Alignment mask – height (voxels)**	34	36 (z-shift -2)	34	36 (z-shift -2)
**Alignment mask – Gaussian**	2	2	2	2
**CC mask – type**	Spherical	Spherical	Spherical	Spherical
**CC mask – radius (voxels)**	6	6	6	6
**Particle number**	2678	2678	2590	2590
**Software**	novaSTA	novaSTA	novaSTA	novaSTA

Supplementary Table 11: Subtomogram averaging parameters for the dataset under transcribing condition.

STA parameters	Pore complex with A10	Pore complex with A10	Pore complex	Asymmetric subunit
**Voxel size (Å)**	5.572	2.786	2.786	2.786
**Box size (voxels)**	72	140	140	140
**Iterations**	3	3	2	2
**Cone angle increment (degrees)**	2 1 1	3 2 1	2 1	2 1
**Cone angle iterations**	5 5 5	10 5 5	5 5	5 3
**In-plane angle increment (degrees)**	2 1 1	3 2 1	2 1	2 1
**In-plane iterations**	15 20 20	20 20 15	15 20	15 10
**Low-pass filter (voxels)**	18 20 30	16 20 39	20 39	20 39
**High-pass filter (voxels)**	1 1 1	1 1 1	1 1	1 1
**Symmetry applied**	C6	C6	C6	C1
**Alignment mask – type**	Cylindrical	Cylindrical	Cylindrical	Cyl. shell (halved)
**Alignment mask – radius (voxels)**	13	56	28	r1=5; r2=28
**Alignment mask – height (voxels)**	34	80	80	80
**Alignment mask – Gaussian**	2	2	2	2
**CC mask – type**	Spherical	Spherical	Spherical	Spherical
**CC mask – radius (voxels)**	6	12	12	5
**Particle number**	2678	2678	2678	16,068
**Software**	STOPGAP	STOPGAP	STOPGAP	STOPGAP

Supplementary Table 12: Subtomogram averaging parameters for the dataset under untreated condition.

STA parameters	Pore complex with A10	Pore complex with A10	Pore complex	Asymmetric subunit
**Voxel size (Å)**	6.24	3.12	3.12	3.12
**Box size (voxels)**	72	140	140	140
**Iterations**	3	3	2	2
**Cone angle increment (degrees)**	2 1 1	3 2 1	2 1	2 1
**Cone angle iterations**	5 5 5	10 5 5	5 5	5 3
**In-plane angle increment (degrees)**	2 1 1	3 2 1	2 1	2 1
**In-plane iterations**	15 20 20	20 20 15	15 20	15 10
**Low-pass filter (voxels)**	18 20 30	17 22 44	22 44	22 44
**High-pass filter (voxels)**	1 1 1	1 1 1	1 1	1 1
**Symmetry applied**	C6	C6	C6	C1
**Alignment mask – type**	Cylindrical	Cylindrical	Cylindrical	Cyl. shell (halved)
**Alignment mask – radius (voxels)**	13	56	28	r1=5; r2=28
**Alignment mask – height (voxels)**	34	80	80	80
**Alignment mask – Gaussian**	2	2	2	2
**CC mask – type**	Spherical	Spherical	Spherical	Spherical
**CC mask – radius (voxels)**	6	12	12	5
**Particle number**	2590	2590	2590	15,540
**Software**	STOPGAP	STOPGAP	STOPGAP	STOPGAP

Supplementary Table 13: Subtomogram averaging parameters for classification. Parameters used for de novo classification of the pore complex in the transcribing and untreated conditions, and for the rod-focused classification described in the Methods

STA parameters	Pore complex (transcription)	Pore complex (untreated)	Rod density (transcription)	Rod density (untreated)
**Voxel size (Å)**	2.786	3.12	2.786	3.12
**Box size (voxels)**	140	140	140	140
**Iterations**	29	29	24	24
**Cone angle increment (degrees)**	29 x 1	29 x 1	29 x 1	29 x 1
**Cone angle iterations**	29 x 5	29 x 5	29 x 5	29 x 5
**In-plane angle increment (degrees)**	29 x 1	29 x 1	29 x 1	29 x 1
**In-plane iterations**	29 x 10	29 x 10	29 x 10	29 x 10
**Low-pass filter (voxels)**	29 x 39	29 x 44	29 x 39	29 x 44
**High-pass filter (voxels)**	29 x 1	29 x 1	29 x 1	29 x 1
**Symmetry applied**	C6	C6	C1	C1
**Alignment mask – type**	Cylindrical	Cylindrical	Cylindrical	Cylindrical
**Alignment mask – radius (voxels)**	28	28	13	13
**Alignment mask – height (voxels)**	80	80	60	60
**Alignment mask – Gaussian**	2	2	2	2
**CC mask – type**	Spherical	Spherical	Cylindrical	Cylindrical
**CC mask – radius (voxels)**	5	5	5	5
**CC mask – height (voxels)**	x	x	20	20
**Particle number**	2678	2590	2678	2590
**Software**	STOPGAP	STOPGAP	STOPGAP	STOPGAP

Supplementary Table 14: Subtomogram averaging parameters for A10 trimer under transcription condition. Note that for runs performed using novaSTA the parameters for cone angle increment, cone angle iterations, in-plane angle increment, and in-plane iterations correspond to cone angle, con sampling, in-plane angle, and in-plane sampling, respectively.

**STA parameters**	**A10 trimer**	**A10 trimer**	**A10 trimer**	**A10 trimer**
**Voxel size (Å)**	11.144	5.572	2.786	2.786
**Box size (voxels)**	48	60	96	96
**Iterations**	6	6	2	2
**Cone angle increment (degrees)**	6 x 45	30 30 20 15 10 5	20 20 20 15 10 5	2 1
**Cone angle iterations**	6 x 9	5 6 5 5 5 5	4 5 5 5 5 5	5 5
**In-plane angle increment (degrees)**	6 x 48	30 30 30 20 20 10	30 30 30 20 20 10	2 1
**In-plane iterations**	6 x 8	5 6 10 10 10 10	5 6 10 10 10 10	10 15
**Low-pass filter (voxels)**	6 x 18	13 13 13 17 17 17	13 13 18 18 27 27	18 27
**High-pass filter (voxels)**	6 x 1	1 1 1 1 1 1	1 1 1 1 1 1	1 1
**Symmetry applied**	C3	C3	C3	C3
**Alignment mask – type**	Cylindrical	Cylindrical	Cylindrical	Cylindrical
**Alignment mask – radius (voxels)**	8	14	22	22
**Alignment mask – height (voxels)**	20	45	50	50
**Alignment mask – Gaussian**	2	6	2	2
**CC mask – type**	Cylindrical	Spherical	Spherical	Spherical
**CC mask – radius (voxels)**	6	12	5	5
**CC mask – height (voxels)**	20	x	x	x
**Particle number**	1,138,687	168,454	168,358	168,352
**Software**	novaSTA	novaSTA	novaSTA	STOPGAP

Supplementary Table 15: Subtomogram averaging parameters for in situ data.

STA parameters	Pore complex with A10 trimer
Voxel size (Å)	13.576
Box size (voxels)	48
Iterations	3
Cone angle (degrees)	24 24 24
Cone sampling	2 2 2
In-plane angle (degrees)	30 30 30
In-plane sampling	3 3 3
Low-pass filter (voxels)	20 20 20
High-pass filter (voxels)	1 1 1
Symmetry applied	C3
Alignment mask – type	Cylindrical
Alignment mask – radius (voxels)	14
Alignment mask – height (voxels)	22
Alignment mask – Gaussian	2
CC mask – type	Spherical
CC mask – radius (voxels)	6
Particle number	159
Software	novaSTA
