# Supplementary material for: A multimeric pore complex in the poxvirus core revealed by integrative structural analysis
Source: Nat Commun. 2026 Oct 30;17:11292. doi: 10.1038/s41467-026-79013-7 (PMC13722698; doi:10.1038/s41467-026-79013-7)
Supplement: Supplementary file 2 [file 41467_2026_79013_MOESM2_ESM.pdf]

Title: Supplementary Video 1

Description: Intact virion core under *in vitro* transcription conditions. Slicing through the reconstructed tomogram reveals the internal organization of the intact core particle. Scale bar, 100 nm.
