# Supplementary material for: A multimeric pore complex in the poxvirus core revealed by integrative structural analysis
Source: Nat Commun. 2026 Oct 30;17:11292. doi: 10.1038/s41467-026-79013-7 (PMC13722698; doi:10.1038/s41467-026-79013-7)
Supplement: Supplementary file 3 [file 41467_2026_79013_MOESM4_ESM.pdf]

nature portfolio

Corresponding author(s): Boris Bogdanow
Jacomine Krijnse Locker
Beata Turonova

Last updated by author(s): Sep 24, 2026

Reporting Summary

Nature Portfolio wishes to improve the reproducibility of the work that we publish. This form provides structure for consistency and transparency in reporting. For further information on Nature Portfolio policies, see our Editorial Policies and the Editorial Policy Checklist.

Statistics

For all statistical analyses, confirm that the following items are present in the figure legend, table legend, main text, or Methods section.

n/a	Confirmed	
☐	☒	The exact sample size (*n*) for each experimental group/condition, given as a discrete number and unit of measurement
☐	☒	A statement on whether measurements were taken from distinct samples or whether the same sample was measured repeatedly
☐	☒	The statistical test(s) used AND whether they are one- or two-sided<br>*Only common tests should be described solely by name; describe more complex techniques in the Methods section.*
☒	☐	A description of all covariates tested
☒	☐	A description of any assumptions or corrections, such as tests of normality and adjustment for multiple comparisons
☐	☒	A full description of the statistical parameters including central tendency (e.g. means) or other basic estimates (e.g. regression coefficient) AND variation (e.g. standard deviation) or associated estimates of uncertainty (e.g. confidence intervals)
☐	☒	For null hypothesis testing, the test statistic (e.g. *F*, *t*, *r*) with confidence intervals, effect sizes, degrees of freedom and *P* value noted<br>*Give P values as exact values whenever suitable.*
☒	☐	For Bayesian analysis, information on the choice of priors and Markov chain Monte Carlo settings
☒	☐	For hierarchical and complex designs, identification of the appropriate level for tests and full reporting of outcomes
☒	☐	Estimates of effect sizes (e.g. Cohen's *d*, Pearson's *r*), indicating how they were calculated

Our web collection on statistics for biologists contains articles on many of the points above.

Software and code

Policy information about availability of computer code

Data collection	SerialEM (multiple versions used across data collection - recored in Mdoc files in the EMPIAR repositories listed below)
Data analysis	CTFFIND4 (v.4.1), Gctf (v.1.06), cryoCAT (v.0.7.1), novaSTA (currently unversioned, https://github.com/turonova/novaSTA), novaCTF (currently unversioned, https://github.com/turonova/novaCTF), IMOD/eTomo (v.4.12.17), TANGO (v.0.7.1), PyMOL (v.3.2.0a), Cytoscape (v.3.10.1) with the XlinkCyNET plugin, UCSF ChimeraX (v.1.10.1) with ArtiaX plugin (v.1.5), Assembline (v.1.03), AlphaFold3 (v.3.0.1, local installation), AF3x (v.0.0.0.20250316, local installation), GAPSTOP(TM) (v.1.0), STOPGAP (v.0.7.5), Dragonfly (v.2025.1), Napari (version not recorded), Scout (v. 2), MAFFT (via ConSurf web server accessed in July 2026), ProtTest (v. 3.4.2), ConSurf (web server accessed in July 2026).<br><br>Code availability:<br>All code required to reproduce the analyses presented in this study is publicly available within the cryoCAT [https://github.com/turonova/cryoCAT] toolbox or at Zenodo under accession code [https://doi.org/10.5281/zenodo.17817056]. The analysis scripts specific to this study are available at Zenodo [https://doi.org/10.5281/zenodo.21479484]. The model used for 3D U-Net–based core picking as well as a tutorial for the training and segmentation are publicly available on Zenodo under accession code [https://doi.org/10.5281/zenodo.20262954].

For manuscripts utilizing custom algorithms or software that are central to the research but not yet described in published literature, software must be made available to editors and reviewers. We strongly encourage code deposition in a community repository (e.g. GitHub). See the Nature Portfolio guidelines for submitting code & software for further information.

Data

Policy information about availability of data

All manuscripts must include a data availability statement. This statement should provide the following information, where applicable:

- Accession codes, unique identifiers, or web links for publicly available datasets
- A description of any restrictions on data availability
- For clinical datasets or third party data, please ensure that the statement adheres to our policy

The electron microscopy density maps generated in this study have been deposited in the Electron Microscopy Data Bank (EMDB) under accession codes EMD-57290 [https://www.ebi.ac.uk/emdb/EMD-57290] (pore complex with surrounding A10 trimers from in vitro cores, transcription condition), EMD-57291 [https://www.ebi.ac.uk/emdb/EMD-57291] (pore complex with surrounding A10 trimers from in vitro cores, untreated condition), EMD-57292 [https://www.ebi.ac.uk/emdb/EMD-57292] (pore complex, transcription condition), EMD-57293 [https://www.ebi.ac.uk/emdb/EMD-57293] (pore complex, untreated condition), EMD-57294 [https://www.ebi.ac.uk/emdb/EMD-57294] (single asymmetric subunit of the pore complex from in vitro cores, transcription condition), EMD-57295 [https://www.ebi.ac.uk/emdb/EMD-57295] (single asymmetric subunit of the pore complex from in vitro cores, untreated condition), EMD-58326 [https://www.ebi.ac.uk/emdb/EMD-58326] (A10 trimer from in vitro cores, transcription condition), EMD-58327 [https://www.ebi.ac.uk/emdb/EMD-58327] (A10 trimers contacting the outer surface of the vaccinia virus pore complex, transcription condition), EMD-58328 [https://www.ebi.ac.uk/emdb/EMD-58328] (A10 trimers contacting the outer surface of the vaccinia virus pore complex, untreated condition), and EMD-58349 [https://www.ebi.ac.uk/emdb/EMD-58349] (pore complex from in situ cores).
The raw tilt series generated in this study have been deposited in the Electron Microscopy Public Image Archive (EMPIAR) under accession codes EMPIAR-13805 [https://www.ebi.ac.uk/empiar/EMPIAR-13805] (transcription condition), EMPIAR-13803 [https://www.ebi.ac.uk/empiar/EMPIAR-13803] (untreated condition) and EMPIAR-13806 [https://www.ebi.ac.uk/empiar/EMPIAR-13806] (in situ tilt series). The previously published raw tilt series used in this study are available in EMPIAR under accession code EMPIAR-11674 [https://www.ebi.ac.uk/empiar/EMPIAR-11674] (untreated condition).
The mass spectrometry proteomics data generated in this study have been deposited in the ProteomeXchange Consortium via the PRIDE partner repository under accession code PXD076400 [http://proteomecentral.proteomexchange.org/cgi/GetDataset?ID=PXD076400].
The AlphaFold3 and AF3x predicted models, additional predictions, analysis scripts and derived data generated in this study – including the oligomeric-state sweeps, the E6 hexamer seed scan, the rod candidate models, the L3 substitution constructs, and the conservation analyses – have been deposited at Zenodo under accession code [https://doi.org/10.5281/zenodo.21479484].
The previously published structural model used in this study is available in the Protein Data Bank under accession code 8P4K [https://doi.org/10.2210/pdb8P4K/pdb].
Source data are provided with this paper.

Research involving human participants, their data, or biological material

Policy information about studies with human participants or human data. See also policy information about sex, gender (identity/presentation), and sexual orientation and race, ethnicity and racism.

Reporting on sex and gender	n/a
Reporting on race, ethnicity, or other socially relevant groupings	n/a
Population characteristics	n/a
Recruitment	n/a
Ethics oversight	n/a

Note that full information on the approval of the study protocol must also be provided in the manuscript.

Field-specific reporting

Please select the one below that is the best fit for your research. If you are not sure, read the appropriate sections before making your selection.

☒ Life sciences ☐ Behavioural & social sciences ☐ Ecological, evolutionary & environmental sciences

For a reference copy of the document with all sections, see nature.com/documents/nr-reporting-summary-flat.pdf

Life sciences study design

All studies must disclose on these points even when the disclosure is negative.

Sample size	No statistical sample-size calculation was performed. Sample sizes (particle and tomogram numbers) were determined by the data acquired.
Data exclusions	No data was excluded from the analyses.

Replication	Findings were reproduced across two independent conditions (transcription and untreated) and confirmed in situ; the XL-MS used two biological replicates.
Randomization	n/a
Blinding	n/a

Reporting for specific materials, systems and methods

We require information from authors about some types of materials, experimental systems and methods used in many studies. Here, indicate whether each material, system or method listed is relevant to your study. If you are not sure if a list item applies to your research, read the appropriate section before selecting a response.

Materials & experimental systems

n/a	Involved in the study	
☒	☐	Antibodies
☐	☒	Eukaryotic cell lines
☒	☐	Palaeontology and archaeology
☒	☐	Animals and other organisms
☒	☐	Clinical data
☒	☐	Dual use research of concern
☒	☐	Plants

Methods

n/a	Involved in the study	
☒	☐	ChIP-seq
☒	☐	Flow cytometry
☒	☐	MRI-based neuroimaging

Eukaryotic cell lines

Policy information about cell lines and Sex and Gender in Research

Cell line source(s)	HeLa, DSMZ (ACC 57)
Authentication	n/a
Mycoplasma contamination	no
Commonly misidentified lines (See ICLAC register)	HeLa is not on the ICLAC register of commonly misidentified cell lines.

Plants

Seed stocks	n/a
Novel plant genotypes	n/a
Authentication	n/a
