# Supplementary material for: A multimeric pore complex in the poxvirus core revealed by integrative structural analysis
Source: Nat Commun. 2026 Oct 30;17:11292. doi: 10.1038/s41467-026-79013-7 (PMC13722698; doi:10.1038/s41467-026-79013-7)
Supplement: Supplementary file 4 [file 41467_2026_79013_MOESM5_ESM.pdf]

nature portfolio

Peer Review File

A multimeric pore complex in the poxvirus core revealed by integrative structural analysis

Corresponding Author: Dr Beata Turoňová

This file contains all reviewer reports in order by version, followed by all author rebuttals in order by version.

Version 0:

Reviewer comments:

Reviewer #1

(Remarks to the Author)
The authors have responded carefully to the previous comments and substantially improved the manuscript. The structural and functional conclusions are now more appropriately separated; I8 is presented as a tentative candidate for the central rod density, and the additional analyses provide stronger support for the proposed E8/E6/L3 architecture. I also appreciate the authors' transparent correction of the rod-displacement analysis and removal of the unsupported claim that rod mobility increases during transcription.

During revision, Calcraft et al. published an independent study, "In situ structure of the poxvirus portal complex," which also identifies E8, E6, and a hexamer-of-dimers arrangement of L3 as the principal components of the poxvirus portal. This reduces the novelty of the molecular assignment but provides strong independent support for the main structural conclusions of the present manuscript. The current work remains valuable because of its complementary XL-MS analysis, systematic candidate testing, automated particle-localization workflow, transcription-state classification, and analysis of the central luminal density.

Before publication, the authors should cite and discuss the new paper and clarify the similarities and differences between the two studies. In particular, Calcraft et al. report no detectable change in portal architecture after NTP treatment, whereas the present study identifies an uncapped class lacking E8 and L3 densities under transcription conditions. This difference may reflect experimental or classification differences, but it should be discussed directly. The uncapped class should therefore be described as a state observed under the specific transcription conditions used here, rather than as a general consequence of transcription.

I have only a few additional minor comments:

The L3 substitution analysis should be described as an in silico AlphaFold3 sensitivity analysis, not as direct experimental validation.
The authors should provide the underlying numbers for the statement that approximately 90% of mapped cross-links satisfy the structural model.
The number of uncapped class-3 particles is inconsistent between the Results and Figure 5 legend, with 1,467 and 1,397 particles reported, respectively.
Statements that the approximately 2 nm clearance is "sufficient" for mRNA passage should instead state that it is geometrically compatible with passage of single-stranded RNA.

Overall, the authors have addressed the major concerns. Subject to incorporation of the newly published study and correction of these minor issues, I support publication.

(Remarks on code availability)
The code is well annotated and can be replicated using a notebook. Very nice!

Reviewer #2

(Remarks to the Author)
The revised manuscript resolves some of the concerns on the identification of the pore components through clarification of the mass spectrometry, additional AF modelling and interface analysis such as sequence conservation. A few points remain incompletely addressed:

1/ “the pore-like structures themselves were described only recently (...) The present work is therefore the first to link a specific molecular assembly to this proposed function.”

It depends what the authors mean by “recently”. On one hand, the pore-like structures were reported in 2005 in the first cryo-ET of virions (10.1073/pnas.0409825102): “We also detected pore-like formations spanning through the viral core membrane, which could be responsible for the export of newly transcribed RNA to the cytoplasm in the early stages of infection.” On the other hand, a paper published after the receipt of the revised manuscript links a specific set of proteins to early mRNA transcription (10.1038/s41586-026-10856-2). These results should be discussed here.

2/ Modelling:
2a. Oligomeric states: the modelling is a very useful addition to the manuscript. It shows pentameric assemblies with good (or sometimes top) scores. Of course, pentamers are important in introducing curvature in icosahedral and non-icosahedral (e.g. HIV core) capsids. This is worth discussing in the main text, including whether the authors think (or have evidence that) these pentameric objects are present in their imaging dataset or not.

2b. Given the resolution of the reconstruction, improving the modelling of E6 would add confidence in the final proposed atomic model. A couple of approaches have not been mentioned:
- Given A10 trimers are proposed to template/constrain the E6 conformation, does their inclusion in AF modelling improve E6 modelling (all neighboring trimers beyond the reasonable token limit but not required).
- “E6 hexamer was treated as two rigid bodies corresponding to its two domains.”: the comment that E6 seems to be made of 3 domains rather than 2 was not addressed in the authors’ response or revised manuscript.
- A potentially better way to approach this, might be to use iterative density-guided modelling which can dramatically improve AF modelling providing more constraints than AFx (e.g. as in PHENIX, 10.1038/s41592-022-01645-6).

Other points:
- In response to comment #3 (Reviewer #2), the authors make the interesting point that the core wall presents some density but no distinct structure, and hence is likely to adopt a different symmetry or molecular organization. This should be mentioned in the manuscript.
- The channel is described as mostly electropositive except for the domain 1 of E6, which is proposed to interact with I8. This is unlike most RNA/DNA conduits, which are in general highly electronegative to avoid sticking of the nucleic acid to the channel sides. What is the expected mechanism or molecular feature proposed here to explain this unexpected charge distribution?
- L. 596, “Crosslinks further confirmed interactions both within the pore complex and between its components and the A10-palisade, supporting the structural arrangement revealed by STA.” This could be toned down since many of the interfaces are not directly validated by crosslinking when poorly modelled structures are excluded.
- “E6 is also required for MV assembly (Resch et al., 2009), which may relate to the contacts we observe between E6 and the surrounding A10 trimers.”: Given the lack of contacts between A10 trimers in the palissade layer, wouldn’t the core wall be expected to be driving assembly while A10/E6 control the shape of the particle (cf. comment above on pentamers and Datler et al, 2024)?

(Remarks on code availability)

Open Access This Peer Review File is licensed under a Creative Commons Attribution 4.0 International License, which permits use, sharing, adaptation, distribution and reproduction in any medium or format, as long as you give appropriate credit to the original author(s) and the source, provide a link to the Creative Commons license, and indicate if changes were made.
In cases where reviewers are anonymous, credit should be given to 'Anonymous Referee' and the source.
The images or other third party material in this Peer Review File are included in the article's Creative Commons license, unless indicated otherwise in a credit line to the material. If material is not included in the article's Creative Commons license and your intended use is not permitted by statutory regulation or exceeds the permitted use, you will need to obtain permission directly from the copyright holder.
To view a copy of this license, visit https://creativecommons.org/licenses/by/4.0/

We thank both reviewers for their careful and constructive assessment of our manuscript. Their comments have substantially improved the work, and we have addressed all points raised.

Two changes cut across the whole manuscript and are worth stating up front. First, following the reviewers' assessment that our functional interpretation exceeded what the data support, we have reduced the interpretation throughout: the pore is now presented as a candidate mRNA-release structure and I8 as a plausible, tentative assignment for the central rod density, and residue-level claims have been retained only where supported by orthogonal evidence – evolutionary conservation, cross-linking restraints, or, in the case of the L3 inter-protomer β-sheet, a direct substitution test. Second, in re-examining the analysis of rod movement in response to Reviewer 2, we found that the axial-shift distributions had been derived from an incorrect particle table. We have corrected this, repeated the analysis, and updated the affected figures; the reviewer's underlying point holds in both cases, and we have withdrawn the claim that the rod undergoes increased axial movement under transcription conditions.

For full transparency, the revised manuscript marks all added text in green and retains removed text as strikethrough, so that every change can be traced. Minor corrections such as typographical fixes are not marked in this way.

Reviewer comments are reproduced in full below, with our responses in italics. References to figures, tables and page numbers are to the revised manuscript.

Reviewer #1 (Remarks to the Author):

A. Summary of the key results

This manuscript reports the structural characterization of a pore-like complex in the vaccinia virus core that the authors propose functions in the export of early viral mRNA transcripts. The work builds on prior observations that the poxvirus core surface contains abundant A10 trimers interspersed with rarer ring-like or pore-like structures surrounded by six A10 trimers. These structures had been proposed to serve as mRNA release sites, but their molecular identity and architecture were not known.

Using cryo-electron tomography, subtomogram averaging, deep-learning-based particle picking, AlphaFold3/AF3x modeling, and cross-linking mass spectrometry, the authors assign the pore complex to a multimeric assembly composed of E8, E6, and L3. E8 and E6 form hexameric rings, while L3 forms a higher-order basal assembly. The authors also identify a central rod-like density in the pore lumen and propose the viral RNA helicase I8 as a likely candidate for this density. They further report transcription-associated remodeling of the pore, including an uncapped state lacking densities assigned to E8 and L3.

Overall, this is a strong and exciting structural biology study. The authors address a long-standing question in poxvirus biology: how early mRNAs synthesized within the viral core exit into the cytoplasm. The structural work is impressive, the integrative strategy is creative, and the automated particle-picking and segmentation workflow represents a technically sophisticated advance for cryo-ET analysis of complex viral assemblies.

We thank this reviewer for the positive assessment of our work and for his/her reviewing efforts. We address the specific comments point by point below.

B. Originality and significance

The study is highly original and significant. The molecular identity and architecture of the poxvirus core pore have remained unresolved despite prior visualization of ring-like structures in vaccinia virus cores. This work provides the most detailed molecular model to date of this complex and offers a plausible structural framework for understanding transcript release from the poxvirus core.

The significance is twofold. First, the biological question is important because early transcription occurs within the poxvirus core, yet the route by which newly synthesized mRNAs exit the core remains unclear. Second, the technical workflow developed here, combining 3D U-Net segmentation, template matching, surface-normal filtering, subtomogram averaging, XL-MS, and structure prediction, is likely to be useful beyond this specific system.

The work is also well connected to previous studies of poxvirus core architecture, A10 trimers in the palisade layer, E6 involvement in pore-like structures, and biochemical studies implicating E8, E6, L3, and I8 in early transcription. However, some of the functional conclusions are less directly supported than the structural conclusions. The claim that this complex is the mRNA export pore remains plausible but indirect. The manuscript would be stronger if the authors framed the work as a major structural discovery that supports a candidate mRNA release pathway, rather than as a complete mechanistic demonstration of mRNA export.

We agree and have followed this recommendation throughout. The title, abstract, introduction and discussion now frame the work as a structural discovery that supports a candidate mRNA-release pathway, rather than a demonstration of mRNA export. Functional interpretation is now clearly separated from the structural findings, and statements regarding export activity and the assignment of I8 are qualified accordingly.

C. Data and methodology: **validity of approach, quality of data, quality of presentation**

The data and methodology are generally very strong. The combination of cryo-ET, subtomogram averaging, XL-MS, AF3/AF3x modeling, and in situ validation is appropriate and cutting-edge. The authors developed an impressive workflow for detecting rare pore complexes on the complex surface of vaccinia cores. The use of deep-learning-based segmentation, template matching, surface mesh generation, surface-normal filtering, and particle cleaning is a major strength of the study.

The structural assignment of E8, E6, and L3 is supported by multiple lines of evidence, including density fitting, cross-linking mass spectrometry, predicted oligomeric assemblies, and prior biological knowledge. The overall quality of the maps appears sufficient to support the main architectural conclusions. The cross-linking data are particularly valuable as an independent constraint on the model.

There are, however, several places where the methodology and interpretation require clarification. The rationale for candidate selection from the XL-MS data should be explained more clearly. The authors filter A10 interactors in part based on molecular weight, reasoning that pore subunits should be larger than A10. This assumption is not obvious, since smaller proteins could form larger oligomeric assemblies or contribute repeated density. The authors should justify this filtering criterion and clarify whether alternative candidates were considered and excluded.

We agree that the basis for candidate selection deserves a clearer explanation, as it rested on several considerations rather than on a single criterion. We would like to stress at the outset that this selection concerns candidates for the pore complex only; the central rod-like density is a separate question, addressed in our response to Reviewer 2, point 4.

Candidate pore components were derived from the A10 cross-linking partners, which we now summarize together with their known or predicted functions in Supplementary Table 1. Each plausible candidate was predicted with AF3 across all oligomeric states from monomer to dodecamer (Supplementary Table 2). Predictions compatible with the dimensions of the pore density and with its six-fold symmetry – that is, states with six subunits or multiples thereof (6 and 12) – were then subjected to a global search in ChimeraX (see Methods) to determine their best position and orientation in the density (Supplementary Fig. 7). At the resolution of the reconstruction, this comparison distinguishes whether a candidate can account for the overall size and shape of the density rather than resolving detailed contacts; on this basis, none of the alternative candidates was compatible with the pore density to the extent of the assigned components. Molecular weight entered only as a secondary consideration alongside symmetry compatibility, not as a primary exclusion criterion; compatibility with the density, together with the cross-linking and prior functional data, is what distinguishes the assigned subunits.

We have revised the Results text to state this scope and selection procedure explicitly.

The modeling of L3 also requires a more detailed explanation. The manuscript states that an L3 hexamer was predicted, but that the map was best explained by adding additional L3 protomers to form a higher-order basal assembly. Since the L3 dodecamer did not converge reliably in AF3/AF3x, the final model appears to rely heavily on density-guided fitting. The authors should clarify whether independent data support this arrangement, whether alternative oligomeric models were tested, and how strongly the current density distinguishes among them.

We agree that the L3 basal assembly is the least constrained part of the model, and we have substantially expanded both the description of the modeling and the supporting analysis.

We first clarify the AF3 behavior: neither AF3 nor AF3x converged on a dodecamer. Predictions yielded either highly overlapping hexamers or two stacked hexamers of approximate D6 symmetry (Supplementary Fig. 10). The basal arrangement was therefore not obtained from prediction alone but built in a density-guided manner, which we now test explicitly against alternative arrangements.

To assess whether the density distinguishes among oligomeric arrangements, we placed a single L3 protomer into one lobe of the basal density and expanded it under C2–C12 symmetry, fitting each expansion to the density (Supplementary Table 3). Expansions corresponding to divisors of six (C2, C3, C6) placed all protomers into density and retained high correlation about the mean (~0.3–0.4), whereas the other symmetries placed subunits away from the lobes and fitted markedly worse (~0.1–0.2). This pattern is the expected signature of a six-fold ring: only symmetries commensurate with six place subunits onto the true lobe positions. Lower divisors (C2, C3), while correctly placed, account for only part of the density, leaving lobes unoccupied; the full C6 expansion fills all six lobes (highest overlap among the tested symmetries; Supplementary Table 3), consistent with all six positions being occupied – as confirmed independently by the six individually fitted protomers (below).

As an independent test that does not rely on symmetry expansion, we fitted six L3 protomers individually into the six lobes of each ring. All six gave closely comparable fits (Supplementary Table 3), indicating that each of the six positions corresponds to genuine, comparably occupied density rather than an arrangement imposed by applying the symmetry. The same analysis applied to both the inner and outer rings of the basal assembly gave consistent results.

Regarding independent, non-density support: the cross-linking data place L3 within the complex, and the intra-L3 cross-links are compatible with the compact double-ring model – the linked residue pairs are satisfiable within a protomer, within a ring, and across the inner and outer rings – so the arrangement is consistent with, though not uniquely determined by, the cross-linking. We have also softened our statement regarding A3: L3 cross-links to A3, which has been proposed to form the core wall, but as this assignment is itself not yet firmly established we now present the L3–A3 interaction as indirect evidence for the basal/core-wall location rather than as confirmation.

Taken together, we present the C6 double-ring as the arrangement best supported by the density among those tested – excluded below and above six-fold by fit, and completed at six-fold – and consistent with the cross-linking, rather than as a uniquely determined structure. We have tempered the corresponding statements in the Results and Discussion accordingly.

The central rod density and its assignment to I8 are intriguing, but the evidence remains suggestive rather than definitive. The rod is featureless; the model does not fully match the density across all regions; and the assignment relies on size, prior biological knowledge, electrostatic complementarity, AF3 prediction, and limited cross-linking support. The manuscript should consistently refer to I8 as a likely or plausible candidate rather than as an identified component unless additional validation is provided.

We agree and now refer to I8 only as a plausible candidate for the central rod density throughout the manuscript. Further analyses supporting this assignment are provided in our response to Reviewer 2, point 4.

The in situ experiment is valuable because it shows that a similar architecture can be detected in infected cells. However, the in situ STA is based on only 159 particles and reaches substantially lower resolution than the in vitro maps. It therefore supports the general presence of a similar architecture in cells but cannot validate detailed protein assignments or conformational states. The authors should state this limitation more clearly.

We have revised the framing of this section and now state explicitly that the in situ reconstruction, obtained from 159 particles at ~40 Å, supports the presence of a comparable architecture in cells but is not of sufficient resolution to validate detailed molecular assignments or to distinguish conformational states.

D. Appropriate use of statistics and treatment of uncertainties

The manuscript includes useful quantitative information, such as particle counts, tomogram counts, resolution estimates, class occupancies, cross-link consistency, pore dimensions, and

rod displacement analyses. These are important and generally appropriate. However, the treatment of uncertainty could be improved in several areas. First, the transcription-associated ruptured core phenotype is interpreted mechanistically, but it is unclear whether rupture reflects a biologically meaningful state or a side effect of sample preparation, osmotic stress, detergent stripping, incubation, grid preparation, or freezing. The authors should quantify the fraction of ruptured versus intact cores under transcription and untreated conditions processed in parallel.
They should also test whether uncapped pores are enriched on ruptured cores.

We quantified the fraction of broken cores in each class to test whether the uncapped class (class 3) is associated with core rupture. Broken cores are more numerous than intact cores across all classes, so we compared the fraction of broken cores per class rather than absolute counts (see Fig. 5B). The uncapped class shows 74.6% broken particles, within the range of the capped classes (57–78%) and not elevated relative to them; the same holds at the level of individual cores (73% of class-3 cores broken). If uncapping were a consequence of rupture, this class would be expected to show a higher broken fraction than the capped classes, which is not observed. We therefore find no enrichment of the uncapped state on broken cores. We note that broken cores predominate in all classes, so we do not interpret these data as excluding any effect of core state in general; rather, they indicate that the uncapped state specifically is not explained by core rupture. This analysis is shown per core in Supplementary Fig. 20, where broken cores are marked.

Second, the interpretation of the uncapped pore class would benefit from more rigorous statistical support. The authors report that this class appears under transcription conditions, but it remains unclear whether the class is associated with specific core states, core rupture, local structural damage, or transcriptional activity. Additional quantification and controls would help establish the robustness of this observation.

As described above, we have added quantification of core state per class and find no enrichment of the uncapped class on broken cores, indicating that uncapping is not linked to core rupture. Because the uncapped class occurs on intact cores as well, we do not consider a further analysis of local structural damage (e.g. proximity to rupture sites) to be warranted. Consistent with this, uncapped pores are observed in both top and side views across different regions of the cores (Supplementary Fig. 19), rather than being confined to particular locations. Finally, as we have now reduced our functional claims regarding the uncapped state, we consider the present statistical analysis sufficient to support the observation as reported.

Third, the rod-focused analysis needs more methodological detail. The authors should explain how the rod focus was achieved, whether masking was used, how the mask was defined, whether C1 or C6 symmetry was applied, and how rod alignment was distinguished from pore alignment. Given the modest number of pore particles and the classification into multiple states, the reliability of the rod signal should be discussed more explicitly.

The methodology and full parameters for the rod-focused analysis, including the masking, were already provided in the Methods and Supplementary Table 13 (originally Supplementary Table 4). We have now clarified in the Methods text what the focused mask covers: it encloses

only the central rod and excludes the surrounding pore rings, so that alignment was driven by the rod rather than the pore walls. C1 symmetry was applied, as the rod does not share the six-fold symmetry of the pore. Rod-focused and full-pore alignments were run independently, and the two sets of orientations were compared to distinguish rod movement from pore movement (Supplementary Fig. 21A,B). On reliability: the rod is directly visible in individual pore complexes in raw tomogram slices, before averaging (Fig. 1, Supplementary Fig. 19), and is retained in the reconstruction without imposed symmetry (see our response to point 9 below). Focused classification converged to a single, featureless class in both conditions, indicating that the rod is consistently present but structurally heterogeneous, or of a symmetry incommensurate with the hexameric pore, which prevents coherent averaging.

Fourth, the authors state that approximately 90% of mapped cross-links are consistent with the structure. This is an important validation, but the analysis should clarify how flexible or low-confidence regions were handled. Were cross-links involving flexible regions excluded, retained, or interpreted separately? Did any cross-links suggest unexpected contacts involving flexible segments? This should be stated clearly.

Low confidence regions (pLDDT < 50) were excluded from the plotting of the cross-links. This eliminates most of residues with high flexibility.

E. Conclusions: robustness, validity, reliability

The central structural conclusions are robust and well supported. The data strongly support the presence of a pore-like complex in the vaccinia virus core and provide compelling evidence that E8, E6, and L3 are major components of this assembly. The integrative structural model is plausible, internally consistent, and represents an important advance in understanding poxvirus core architecture.

The functional conclusions are less robust. The manuscript convincingly identifies and models a pore-like structure, but it does not directly show that mRNA exits through this pore, that transcript release depends on this structure, or that I8 is definitely the central rod. The in vitro transcription assay supports RNA production under the experimental conditions, but it does not directly connect RNA release to the pore complex. It is also unclear whether newly synthesized RNA is released from intact cores or whether some RNA could be produced or released from damaged or ruptured cores.

Similarly, the transcription-associated loss of E8 and L3 densities is intriguing, but its biological meaning remains uncertain. It could represent active transport, termination, disassembly, damage, or preparation for uncoating. The current data do not distinguish among these possibilities.

The conclusions should therefore be softened in the title, abstract, and discussion. Phrases such as “mRNA export pore” or “mRNA release machinery” should be qualified as “candidate,” “putative,” or “proposed” unless more direct functional evidence is added. With this adjustment, the manuscript would present a strong and reliable structural discovery while appropriately acknowledging the remaining mechanistic uncertainty.

We have softened the title, abstract and discussion accordingly; references to mRNA export and to I8 are now consistently qualified as candidate or proposed.

F. Suggested improvements: experiments, data for possible revision

The manuscript would be substantially strengthened by addressing the following points.

1. The authors should more clearly separate structural discovery from functional interpretation. The data strongly support the identification of a pore-like E8/E6/L3 complex, but the claim that this is the mRNA export pore remains indirect. The title, abstract, and discussion should be revised to avoid overstating functional conclusions.

The title, abstract and discussion have been revised to separate the structural discovery from the functional interpretation, as described in our responses above.

2. The in vitro transcription experiment should be interpreted more cautiously. The authors show increased RNA absorbance under transcription conditions, but this does not directly demonstrate RNA export through the pore. It would be useful to distinguish RNA synthesized inside intact cores from RNA released after core rupture or RNA generated in association with damaged cores. If cores are broken and UTP is supplied, the authors should address whether RNA synthesis could occur outside intact cores or from exposed transcription complexes.

The absorbance assay reports the total amount of RNA produced and cannot distinguish core-associated from free RNA; the total yield is close to the detection limit of the assay, so sub-fractionating it into core-associated and released pools is not feasible. We agree, therefore, that this measurement reports RNA synthesis rather than export through the pore, and we have adjusted the text to state this explicitly and to avoid implying that the assay demonstrates export. We would note, however, that transcription in this system is carried out by the virion-associated machinery packaged within the core, so RNA synthesis originates from core-derived transcription complexes whether or not the core remains fully intact; and, as shown in our analysis of core state (see above), the transcription-associated pore states are not enriched on broken cores. The assay thus supports that transcription occurs, consistent with the structural changes we observe, without our claiming it as direct evidence of export.

3. The interpretation of ruptured cores should be strengthened or toned down. The authors should quantify intact versus ruptured cores in transcription and untreated conditions processed in the same way. They should also assess whether ruptured cores are enriched for uncapped pores and whether rupture correlates with transcriptional activity or sample preparation conditions.

As addressed above (in section D), we quantified intact versus ruptured cores in both conditions and found no enrichment of the uncapped class on ruptured cores, indicating that uncapping is not a consequence of rupture. Whether rupture itself correlates with transcriptional activity or sample preparation concerns the mechanism of core breakage — a distinct question from the pore structure reported here, which we are investigating in separate ongoing work. We have accordingly tempered the interpretation of the ruptured-core phenotype in the present manuscript, presenting it as an observation associated with the transcription condition rather than assigning it a mechanistic cause.

4. The particle-picking and segmentation pipeline should be described more clearly in the main text. The supplementary information is helpful, but because this workflow is central to the discovery and likely valuable to other groups, the main text should provide a more accessible explanation of how segmentation, template matching, surface mesh generation, surface-normal filtering, and particle cleaning were integrated.

We have revised the main-text description of the picking and segmentation pipeline to explain more clearly how its components – segmentation, template matching, surface mesh and point-cloud generation, and surface-normal-based particle cleaning – are integrated, and how this recovers side-oriented pores that manual picking would miss. To make the workflow more accessible, the schematic is now presented as a main-text figure (Fig. 2). Full details remain in the Methods.

5. The rationale for selecting candidate pore components from XL-MS should be clarified. The molecular-weight filter used to identify candidate proteins should be justified, and alternative candidates should be discussed. A summary table listing vaccinia proteins linked to RNA transport or early transcription, their XL-MS support, predicted or known functions, and whether they were considered or excluded would strengthen this section.

We have clarified the basis for candidate selection and expanded the summary table as suggested. We would like to clarify the scope of the candidate search: our aim was to assign the observed pore density rather than to enumerate the proteins involved in early transcription or RNA transport in general. The candidate set is therefore defined by cross-linking to A10 – the layer against which candidates can be positioned in the density – rather than by function (the role of molecular weight in candidate selection is addressed in our response in section C above); a protein associated with early transcription but not cross-linked to A10, or without density compatible with its size and shape, is not a candidate for this structure. Accordingly, Supplementary Table 1 lists the A10 cross-linking partners together with their known or predicted functions, their cross-linking support, the oligomeric states tested, and whether each was considered or excluded.
Once the pore components were assigned, we additionally tested candidates for the central rod. For this we generated AF3 predictions combining the assigned pore hexamers (E6, E8 and L3) with individual candidate proteins associated with early transcription, and examined where each candidate was placed. L4 monomer, dimer, and trimer were positioned within the pore but with different shape and size to account for the observed rod density; two chains of H1 were placed outside the pore; and H6 placed also inside the pore is substantially smaller than the density. Only I8 was consistently positioned within the lumen at a size compatible with the rod. Given the limited resolution of the rod density, this comparison assesses position and overall size rather than detailed fit, and we therefore do not assign the rod at the present resolution. The predictions are available at Zenodo (see Data availability statement in the manuscript).
Aside from the central rod, the hexamers of E6 and E8 and the dodecamer of L3 account for the ordered pore density, so we did not find evidence of an additional unassigned component that would warrant a broader candidate search. We have also moderated our functional interpretation throughout the manuscript, so that the analysis is presented specifically as a structural assignment of the pore rather than a general account of early transcription.

6. The authors should include an evidence table for the protein assignments. For each proposed component, the table should summarize density fit, XL-MS support, AF3/AF3x confidence, prior biological evidence, and limitations. This would make the integrative logic more transparent.

We have added an evidence table summarizing, for each proposed component, the fit to the density, the supporting cross-linking restraints, the confidence of the AlphaFold3 predictions, the prior biological evidence, and the limitations of the assignment (Table 2). The cross-linking entries state the restraints that bear on each assignment and on the oligomeric state, rather than repeating the full interaction network, which is shown in Fig. 2. The central rod density is included but indicated as a tentative assignment, with its limitations stated explicitly, so that it is not presented on the same footing as E6, E8 and L3.

7. The L3 model requires further clarification. Because the L3 dodecamer did not converge reliably in AF3/AF3x, the authors should explain how alternative oligomeric arrangements were evaluated and whether any independent evidence supports the proposed hexamer-of-dimers or double-ring arrangement.

Addressed in our response to point C: the L3 oligomeric arrangement was tested by symmetry expansion (C2–C12) and by individual protomer fitting, which together support the six-fold double-ring among the arrangements tested rather than a uniquely determined structure.

8. The assignment of the central rod to I8 should be presented as plausible but not definitive. Stronger support would require additional validation, such as tagging, labeling, depletion, mutant analysis, altered transcription conditions, or a condition in which I8 is specifically absent or perturbed.

I8 is now presented as a plausible candidate for the central rod density throughout. A suitable inducible or temperature-sensitive I8 virus for direct validation is not available to us, and the text has been adjusted to reflect the resulting uncertainty.

9. The authors should discuss whether the central rod density could be influenced by symmetry application, alignment, or averaging artifacts. It would be helpful to show reconstructions generated without imposed symmetry, or additional controls demonstrating that the rod is visible in raw particles or independent reconstructions.

The rod is not an artifact of symmetry or averaging. It is directly visible in individual pore complexes in raw tomogram slices, before any averaging (Fig. 1, Supplementary Fig. 19). Moreover, in the reconstruction calculated without imposed symmetry (Supplementary Fig. 3), the rod is retained and, if anything, its connections to the pore are more pronounced than in the symmetrized map. The rod signal therefore does not depend on C6 application.

10. The rod-focused analysis should include more methodological detail. The authors should specify the mask, symmetry, alignment strategy, classification procedure, and criteria used to assess the robustness of the rod signal.

The parameters for the rod-focused analysis, including the mask definition, were provided in Supplementary Table 4 (rod-classification columns), which now corresponds to Supplementary Table 13. We agree, however, that they should also be stated in the main Methods text, and we have now added an explicit description of the rod-focused mask, symmetry, alignment strategy, and classification procedure there, with a direct reference to Supplementary Table 13. The criteria used to assess the robustness of the rod signal – convergence of focused classification and consistency with the unsymmetrized reconstruction and raw tomogram slices – are addressed in our responses to point #3 in section D and point #9 above.

11. The mechanistic implications of I8 occupying the pore lumen should be discussed more directly. If I8 sits in the channel, how does mRNA pass through the pore? Does RNA pass around I8, does I8 move axially, or does I8 remodel RNA before export? Does the remaining channel diameter support the proposed path? A schematic showing possible RNA transit routes would be useful.

We have expanded the discussion of how mRNA could transit the pore, while keeping it at the level the data support. The channel is wide enough for single-stranded RNA to pass: the modeled clearance between the rod and the surrounding wall is ~2 nm, and the L3, E6 and E8 channels each accommodate single-stranded RNA. The electrostatics are consistent with such a path – the pore channel is predominantly positively charged, and where the E6 lumen is less so, the complementary positive surface of the modeled helicase would line that region. An AF3 prediction placing RNA within the helicase indicates that this arrangement is sterically feasible (Supplementary Fig. 22). Beyond this, we prefer not to specify a transit mechanism: whether RNA passes around the rod, whether the rod moves axially, or whether it remodels RNA cannot be determined from the present data, and we note that the rod's axial position does not differ significantly between transcribing and untreated cores. For the same reason, and given that the assignment of the rod to I8 is itself presented as tentative, we have not included a schematic of transit routes, as it would imply a level of mechanistic certainty the data do not support.

12. The transcription-associated uncapped class should be analyzed more deeply or interpreted more cautiously. The authors should clarify whether loss of E8 and L3 reflects active transport, termination, disassembly, damage, or uncoating. If this cannot be resolved, the text should avoid assigning a specific mechanism to this state.

We have removed the specific mechanistic interpretation of the uncapped state, and the text now states that our data do not allow us to distinguish among active transport, transcription termination, disassembly or damage.

13. The in situ experiment should be framed more clearly. The authors infect cells for 30 minutes, which likely captures early post-entry cores rather than newly assembled virions. The biological rationale for this time point should be stated explicitly.

The biological rationale for the 30-minute time point is now stated in the main text: it captures incoming, post-entry cores undergoing early transcription, before core uncoating and DNA release – the stage at which the pore complex is expected to be functionally relevant.

14. The in situ STA should be interpreted cautiously. The reconstruction is based on a small number of particles and has low resolution. It supports the presence of a similar general architecture in cells but should not be used to validate detailed molecular assignments or conformational states.

We agree. The main text now states explicitly that the in situ reconstruction, obtained from 159 particles at ~40 Å, supports the presence of a comparable architecture in cells but is not of sufficient resolution to validate detailed molecular assignments or to distinguish conformational states.

15. Figure 3 would be clearer if protein names were shown directly in addition to descriptive labels such as outer ring, inner ring, and basal ring. Adding E8, E6, and L3 labels would improve readability.

We have added the protein names (E8, E6, L3) to Figure 4 (originally Figure 3) alongside the descriptive ring labels, as suggested.

16. In Figure 3C, the rainbow coloring is somewhat confusing because all proteins use similar gradients. Distinct gradients or separate color schemes for each protein would make the topology easier to interpret.

We have split the former panel so that the individual protomers are now shown separately and enlarged in Figure 4D (originally Figure 3C), making the N- to C-terminal topology of each protein clearly distinguishable.

17. Formatting issues in the methods should be corrected, including incorrectly rendered underscores and missing superscript or subscript formatting, for example around CO_2 and $MgCl_2$.

We were unable to locate the formatting issues noted in our original manuscript document, where these appear correctly. We did, however, find them in the converted PDF generated by the submission system, and therefore assume they arise during conversion rather than from our source. We will work with the editorial office to ensure the formatting is correctly preserved in the final version.

G. References: appropriate credit to previous work?

The manuscript generally gives appropriate credit to prior work. It cites earlier studies on vaccinia virus core transcription, poxvirus core architecture, A10 trimers in the palisade layer, previous visualization of pore-like structures, E6 involvement in virion assembly and transcriptional competence, L3 function in early transcription, and I8 helicase activity. The authors should ensure that prior work proposing ring-like structures as potential mRNA release sites is clearly distinguished from the new molecular assignment presented here. They should also make clear which aspects of the proposed functional model are supported by previous biochemical work and which are newly inferred from the current structural data. The reference list appears appropriate overall. A concise table linking prior biological evidence for E8, E6, L3, and I8 to the current structural assignments would help readers understand how previous work supports, but does not by itself prove, the proposed export mechanism.

We have addressed these points. A summary table linking the prior biological evidence for E8, E6, L3 and I8 to the current structural assignments has been added (Table 2), indicating for each protein what is established from previous work and citing the relevant studies.
Regarding prior proposals of ring-like structures as mRNA release sites: the pore-like structures themselves were described only recently, and while the existence of mRNA-release sites in the core had been postulated earlier, to our knowledge these proposals were not associated with any defined structure. The present work is therefore the first to link a specific molecular assembly to this proposed function, and we have made this distinction explicit in the Introduction/Discussion.
On distinguishing established from newly inferred aspects of the model: throughout the manuscript, claims derived from previous work are referenced to their source, whereas unreferenced statements represent inferences from the present structural data. We have also substantially reduced the functional claims (see our responses to Reviewer 1, points A/E, and Reviewer 2, point 5), so that the proposed export function is now presented as a hypothesis consistent with the structure rather than as demonstrated.

H. Clarity and context: lucidity of abstract/summary, appropriateness of abstract, introduction and conclusions

The manuscript is generally well written and clearly presented. The abstract and introduction effectively define the biological problem and explain why the poxvirus core pore is important. The figures are generally clear, and the manuscript communicates the structural findings well. The main issue is that the abstract and conclusions currently overstate the functional interpretation. The structural discovery is excellent, but the mechanistic model of mRNA export remains incompletely demonstrated. The abstract should more clearly indicate that the pore is a proposed or candidate mRNA export structure and that I8 is a plausible candidate for the central rod density. The discussion should more explicitly acknowledge what remains unknown, including how RNA enters the L3 region, how RNA passes through or around I8, whether E8/L3 dissociation activates or terminates export, and how this process relates to core rupture and genome release.
In summary, this is a beautiful and interesting paper with high-quality data, a creative technical approach, strong structural analysis, and clear potential impact. The

characterization of the poxvirus core pore is important, providing a plausible molecular explanation for a structure that has been observed but not fully understood. The automated picking and segmentation workflow is especially impressive and represents state-of-the-art cryo-ET analysis.
I am supportive of publication, but I think the manuscript requires revision. The authors should either provide more direct functional or validation data, or soften the language so that the paper is presented as a major structural discovery and a strong framework for future mechanistic studies, rather than as a complete demonstration of the mRNA export mechanism. With these revisions, the manuscript would be highly valuable to both the structural biology and virology communities.

We thank the reviewer for the positive overall assessment. As described in our responses above, we have revised the abstract and conclusions to present the pore as a candidate mRNA-export structure and I8 as a plausible candidate for the central rod density. The Discussion now explicitly acknowledges the open questions the reviewer raises – how RNA enters the L3 region, how it passes the central density, whether E8/L3 dissociation activates or terminates export, and how this relates to core rupture and genome release – as directions for future work. The manuscript is now framed as a structural discovery and a framework for future mechanistic studies rather than a demonstration of the export mechanism.

Reviewer #1 (Remarks on code availability):

Code is open source, and the documentation is available. The documentation is not fully developed, but it is there, so one can theoretically start using it.

We thank the reviewer for noting that the code is open source and usable. We assume the comment on documentation refers to the Jupyter notebook for the machine-learning-based mask generation, which is indeed relatively sparse; the remainder of the pipeline is implemented in the cryoCAT toolkit, which provides full API documentation and dedicated tutorials (https://cryocat.readthedocs.io).

Reviewer #2 (Remarks to the Author):

This cryo-ET study follows a series of recent articles by three different teams that have described vaccinia virus cores by cryo-ET and SPA cryo-EM at increased resolutions, almost 2 decades after the initial structure of the vaccinia virion published in 2005. These works identified the molecular components of the palisade as A10 trimers but could not confirm the identity of the core wall (A3?) and pores that traverse the palisade and core wall providing a connection between the viral core interior and exterior.
Here, Liu et al present cryo-EM reconstructions, AF3 modelling and crosslinking MS that attempt to resolve these pores. They use an original approach to identify the pore in cryo-ET images for sub-tomogram averaging. This is highly challenging given the poor signal-to-noise intrinsic to the dense virions and the paucity of pores on each virion (~20). They attain relatively modest resolutions (in absolute terms, not for cryo-ET) so that the reconstructions alone do not allow de novo modelling of the pores. However, AF3 models partially fit into the

reconstructed maps. While the fit and modelling of oligomers are imperfect, crosslinking mass spectrometry adds confidence in the proposed model.
Finally, the authors focused on a density present at the centre of the pore. They assign it to I8 and compare the structure of the pores when viral transcription is activated. Unfortunately, they do not visualise the emergence of nascent RNAs as has been possible for icosahedral viruses like rotavirus (doi: 10.1038/s41467-019-10236-7) or more recently cystovirus phi6 (DOI: 10.1016/j.molcel.2025.12.025).

It is true that nascent RNA has been visualized in those systems, but this reflects a difference in method rather than in our data. Both cited studies used single-particle cryo-EM, where the high electron dose allows mRNA to be visualized; cryo-ET, operates at a small fraction of that dose. While double-stranded RNA and DNA are dense enough to be observed in tomograms, we are not aware of any validated direct visualization of single-stranded RNA by cryo-ET, which has roughly half the density of its double-stranded counterpart.

This study is significant and provides exciting new hypotheses regarding the structure and function of the vaccinia virus pores. However, the level of certainty about the composition and structure of the pore and its content remain too low, requiring significant additional analyses. Here are my main reservations and comments:

1. The crosslink data is essential to add confidence in the molecular composition of the pore but not easy to analyse as presented. Thus the reader has to follow the authors' focus on interactions, which appear less strongly supported by experimental data than others that are ignored. The size argument is not very convincing since it can become secondary when homo-/hetero-oligomers are taken into account. A couple of analyses may help to better gauge experimental support for the authors' interpretations: please provide the analysis with 1% FDR at the residue level rather than protein level. Also, indicate the most common names and/or function for each hit in Fig 2 b and c (e.g. H1 for the dual phosphatase). It should be clearer that all of these alternate candidate for the pore have been modelled and tested for fit into the reconstructions.

The cross-linking data were already filtered at the residue-pair level, not only at the protein level. As stated in the Methods (XL-MS data analysis), the interaction network was filtered consecutively to 1% FDR at the cross-link spectrum-match, residue-pair, and protein–protein interaction levels, and cross-links mapped onto the structure were filtered to 1% FDR at the spectrum-match and residue-pair levels.

We agree that molecular mass alone is not a reliable criterion, as a smaller protein could contribute through a higher oligomeric state, and candidates were therefore not excluded on mass. All A10 cross-linking partners were predicted with AF3 across oligomeric states from monomer to dodecamer and tested for fit to the pore density; none other than E6, E8 and L3 was compatible with the density in any state tested (Supplementary Fig. 7). The full candidate list, the oligomeric states tested, and the outcome are now given in Supplementary Table 2, and the modeling and fitting rationale is described in our response to point C.

To make the cross-linking hits easier to follow, we have added the common protein names to Fig. 2B and 2C. Functional annotations are provided in the candidate summary table

(Supplementary Table 1) rather than in the figure, where there is no space to include them legibly.

2. The AlphaFold modelling of the pore is generally poorer than expected for vaccinia virus. This particularly true of E6 and of the E6/L3 or E6/E8 interactions. E6 is described as a 2-domain protein (e.g. Supp Fig 9C) but the PAE plot rather suggest a 3-domain organisation (note: the domain/chain boundaries should be indicated to improve readability of the PAE plots). AF relative failure is surprising given the depth of sequences available and the expected functional conservation, which for other vaccinia virus proteins result in reliable predictions (e.g. A10 or the EFC). The low pLDDT for parts of A6 and L3, and high PAE for all "complexes" suggest that additional molecular partners are missing and/or the general organisation of the pore is partially incorrect.

The lower AF3 confidence for the isolated pore components is consistent with assembly that depends on the native lattice context rather than with an incorrect organization. When the components are predicted in isolation, the E6 hexamer is open, its upper portion extending beyond the density; incorporating the cross-links (AF3x) closes the ring and defines the E6–E8 interface, and fitting into the density completes the closure. This progression is quantitative: the E6 hexamer improves from a cross-correlation of 0.48 (AF3) to 0.63 (AF3x) to 0.68 (fitted), with model overflow falling from 0.41 to 0.19, and the full complex from 0.37 to 0.51 to 0.59 (Supplementary Table 5). The closed conformation is thus templated by the surrounding palisade — the six A10 trimers and neighboring components — which is absent from the isolated prediction. In this sense the reviewer is correct that partners are missing from the isolated models; we now state explicitly that closure is lattice-dependent. We have indicated the domain and chain boundaries on the PAE plots (Supplementary Fig. 12 and also 22) to improve readability.

Given the uncertainty around these models, further analyses are required such as a comprehensive analysis of oligomeric states (e.g. 1- to 12-mers) to validate that C6 is the most likely symmetry for AF3, independently of the cryo-EM. Conversely, assuming a hexamer of E6, what is the diversity of structures when more seeds are used (e.g. 75-100 structure predictions)? It would be surprising that AF3 never samples the cryo-EM structure. More analyses should support the E6-L3 and E6-E8 interactions. From the current AF3 and crosslinking MS, there is no real support for the interactions and at >8 Å of resolution the cryo-EM is insufficient to provide definitive proof. Are the proposed inter-molecular surfaces conserved? Are there strong complementarities between the electrostatic surfaces (not evident in Fig. 3D or Supp Fig. 16)?

We validate the six-fold symmetry, and the reproducibility of the AF3 prediction, in three independent ways.
First, a reconstruction calculated without imposing symmetry reproduces the six-fold arrangement directly from the data, in both the transcribing and untreated conditions and in orientations independent of the reference (Supplementary Fig. 3; Methods). The symmetry is therefore an observation from the map, not an assumption imposed during averaging.
Second, we tested whether the fitted models are themselves consistent with six-fold symmetry by symmetry expansion, using the approach described in detail in our response to

Reviewer 1, point C, applied here to all three subunits. Briefly, a single protomer was expanded under C2–C12 symmetry and fitted to the density; only expansions commensurate with six-fold symmetry (C2, C3, C6) place all copies into density and retain high correlation about the mean (~0.3–0.4), whereas non-commensurate symmetries position subunits away from the density and fit markedly worse (~0.1–0.2) (Supplementary Table 3). The same pattern holds for E6, E8 and both L3 rings.

Third, to address the reviewer's specific question about seed diversity, we tested how reproducibly AF3 predicts the E6 hexamer and whether it ever recovers the closed conformation seen in the density. We generated 500 predictions across independent seeds and examined two things separately: the symmetry of each predicted ring, and how open or closed it is – a ring can be six-fold symmetric yet either closed, as in the density, or splayed open (Methods; Supplementary Fig. 13, Supplementary Table 4). For symmetry, we measured the rotation relating neighboring protomers and the regularity of the ring axis: all 500 predictions formed regular six-fold rings (rotation between adjacent protomers 60.0 ± 0.0°, the value expected for a hexamer; axis consistency 0.998, where 1.0 is a perfect ring; flat rather than spiral). AF3 therefore favors the C6 arrangement of E6 robustly and reproducibly, in agreement with the two analyses above. For conformation, we measured how far each ring is from the closed state in two independent ways – the Cα RMSD of each prediction to our fitted E6 model (comparing the rings in every possible protomer alignment, so the value reflects a genuine conformational difference), and the ring radius, which increases as the ring opens. By both measures, none of the 500 predictions reached the closed conformation: the closest was 13.8 Å RMSD and the median 24.7 Å, and every predicted ring was more expanded than the fitted model (radius 51–65 Å).

AF3 thus reliably reproduces the symmetry and topology of E6 but not its closed state – the behavior expected if closure is stabilized by the surrounding lattice rather than being intrinsic to the isolated hexamer. This is consistent with the fit progression described above (E6 improving from 0.48 in the isolated AF3 prediction to 0.68 in the fitted model): adding cross-links (AF3x), or co-predicting E6 together with candidate neighboring components, drives the ring towards closure, which is then completed by fitting into the density. We would therefore argue that AF3 not sampling the closed conformation in isolation is expected and informative, rather than evidence against the model.

With low confidence modelling and low resolution cryo-EM reconstructions, experimental validation (e.g. site-directed mutagenesis or further crosslinking MS) is required to discuss residue-level interpretations throughout the manuscript. As an example, I don't see sufficient evidence to state: "… also forms interprotomer contacts with neighboring E6 monomers at residues Glu117–Lys404 and Arg210–Asp313, stabilizing the upper part of the E6 ring." Fig 3E is also quite hard to read. The beta-sheet extension is interesting but not clearly illustrated.

We agree that residue-level interpretations are not supported at this resolution, and we have revised the manuscript accordingly. The contacts noted by the reviewer (Glu117–Lys404 and Arg210–Asp313) have been removed – conservation analysis shows these positions to be among the most variable in E6, and they are not supported by cross-linking – and we have applied the same criterion throughout, describing interfaces at the level of the interface rather than of individual side-chain contacts.

To provide orthogonal support, we performed evolutionary conservation analysis (ConSurf) for E6, E8 and L3, computed on the individual monomers and therefore independent of the

model, the density and the oligomeric state. Each interface is now assessed against both conservation and the cross-linking data, and the outcome is summarized in Supplementary Table 6 together with the supporting restraints and the resulting level of support. Interfaces resting on the model alone have been softened or removed; those corroborated by orthogonal evidence are retained and described accordingly.
The inter-protomer β-sheet of the inner L3 ring was additionally tested directly: introducing a proline substitution into the paired strands disrupted the predicted pairing, indicating a genuine feature of the interface rather than a prediction artifact (Supplementary Fig. 16). Figure 4E (originally 3E) has been redrawn to show the retained interfaces more clearly.

3. The base of the pore does not show densities for the neighboring components, which are presumably the core wall. Has this density been masked away or is it following a different symmetry? It looks fairly strong in the STA of panel 1D (top right). Is there any reason for the L3 rings to stop at a double-ring structure? That is, would it be possible for L3 to form (part of) the core wall?

The neighboring density has not been masked away. Our alignments used a generous mask that included the surrounding A10 trimers together with the region below them, adjacent to L3 (Fig. 1D). In these maps a density is indeed present below A10, through which L3 pierces, but it remains unstructured; the same is true of the C1 reconstruction calculated without imposed symmetry to validate the six-fold arrangement. The density is therefore present but does not resolve, rather than being excluded by masking.
We interpret this as the core wall not sharing the organization of the pore and its surrounding A10 trimers. Although the A10 trimers are regularly arranged around the pore – unlike elsewhere on the core – the underlying core wall appears misaligned with respect to this extended assembly, so that an irregular or non-six-fold arrangement would not survive averaging. Consistent with this, the core wall has proven difficult to resolve more generally: Datler et al. (2024) obtained a low-resolution density corresponding to an A3 dimer by single-particle analysis but were unable to place it reliably within tomograms. We should note that we have not attempted an alignment focused specifically on this region.
Regarding whether L3 could form part of the core wall: for this to be compatible with our data, L3 would have to adopt a different, irregular arrangement below each pore, since no ordered density beyond the two rings is recovered. We consider this unlikely, but we cannot formally exclude it at the present resolution and we make no claim in either direction.

4. Untreated vs. transcription cores:

- The authors state "Interestingly, unlike in the untreated condition, these cores displayed detached lateral bodies and a decondensed genome inside". The differences induced by the transcription mix should be shown to validate the condensed to uncondensed transition.

This observation is based on direct inspection of the tomograms, in which the detached lateral bodies and the decondensed genome are apparent in cores from the transcription condition but not in untreated cores. We have added Supplementary Fig. 2 comparing the two conditions and clarify in the text that this is a qualitative observation from the tomographic

data rather than a quantified transition. A detailed characterization of this change, including its extent and kinetics, is the subject of a separate study currently in preparation, and we have kept the statement in the present manuscript descriptive accordingly.

- Is the “uncapping” a biochemical change in the pore or a structural change that means the STA “erases” the cap densities? Are there predicted nucleotide-binding sites in A6, E8, L3 (or even I8) that could explain sensitivity to the transcription-inducing buffer? Or is anything else in this buffer likely to affect E8 and L3 recruitment to the pores?

Capped and uncapped pores are observed within the same condition and, importantly, coexist on individual cores, interspersed with one another (see our analysis of class distribution in response to Reviewer 1, points D/F3). This argues against an averaging artifact: were the caps being erased by STA, the effect would apply uniformly rather than to a subset of pores on the same core. It also argues against a buffer effect, since all pores on a given core are exposed to identical conditions; cores incubated without the transcription mix, but otherwise treated and prepared identically, retain E8 and L3. The uncapping therefore appears to reflect a genuine change in pore composition rather than a consequence of averaging, buffer, or core preparation.

- Central density:

(1) Given the distributions presented in Fig 4F, I’m not sure one could propose that “… the rod undergoes increased axial movement when transcription is initiated”. Is there a statistical measure to support this statement? This is not evident from Fig 4 and Supp 14. Wouldn’t you expect the central density to become weaker in uncapped pores?

We thank the reviewer for prompting a closer look at this analysis. In re-examining it, we found that the axial-shift distributions in Figure 4F had been derived from an incorrect particle table; we have corrected this, updated the figure, and repeated the analysis on the correct data. The reviewer's underlying point holds in both cases: the statement is not supported. On the corrected data, the axial-shift distributions in the two conditions have essentially identical spread (transcription SD 1.31 nm, untreated 1.46 nm; Brown–Forsythe test for equal spread $p = 0.93$), so there is no evidence for increased axial movement of the rod under transcription. We have removed this statement and now describe the distributions as comparable, with the rod showing continuous positional variability along the pore axis in both conditions. Regarding density in uncapped pores: the rod is present and continuous in all axial-shift bins and all classes, including the uncapped class (Supplementary Fig. 21B,C), consistent with a stable rod whose position varies rather than a density that weakens upon uncapping.

(2) I8 is a (very) tentative candidate for the inner pore component. Only one compatible intermolecular crosslink is observed. Other crosslinks (e.g. Fig 2) would need to assume I8 is also elsewhere in the core and even then it is not clear if it would be inside (A3 crosslinks) or outside (A4 crosslinks). Have oligomers of I8 been modelled? Its putative function as an RNA helicase would suggest it may form higher oligomeric forms. These would be incompatible with a location inside the pore but a logical analysis to add here if mention of I8 is to stay.

We have modeled I8 oligomers as suggested. AF3 predictions of I8 were generated from monomer to dodecamer (Supplementary Table 8). The monomer is predicted with high confidence (pTM 0.92), whereas all oligomeric states from dimer to dodecamer score poorly (inter-chain ipTM 0.19-0.35), so AF3 does not support I8 forming a stable oligomer, and these low-confidence oligomers did not fit the rod density. A single I8 monomer is compatible with the dimensions of the rod. This agrees with the biochemical characterization of the I8 gene product (NPH-II), an established RNA helicase whose sedimentation properties indicate a monomer (Shuman, PNAS 1992, https://doi.org/10.1073/pnas.89.22.10935), and with the three intramolecular I8 cross-links in our data.
Regarding localization: we agree the cross-linking evidence does not by itself place I8 unambiguously. We observe one cross-link between I8 and A3, in addition to the I8-L3 cross-link. However, neither A3 nor A4 is itself firmly localized in the structure – A3 is proposed to form the core wall and A4 is a putative cement protein – so cross-links to these proteins cannot, on their own, establish whether I8 lies inside or outside the pore. The assignment of I8 to the rod therefore rests on the agreement between the monomeric I8 model and the dimensions and position of the rod density, together with the I8-L3 cross-link, rather than on localization inferred from A3 or A4. Consistent with this, I8/NPH-II is activated by single-stranded RNA (Shuman, PNAS 1992, https://doi.org/10.1073/pnas.89.22.10935), in line with a functional role within the pore lumen. We agree the evidence remains limited, and I8 is presented throughout as a plausible, tentative candidate for the rod density rather than a firmly assigned component (see also our response to point 8 from Reviewer 1).

(3) Why are nascent RNAs not visible? One would expect extra densities to be prominent as seen for dsRNA viruses.

This reflects the different visibility of double- and single-stranded RNA rather than an absence of transcripts. Double-stranded RNA, like DNA, is dense enough to be observed in cryo-EM and cryo-ET, which is why it is prominent in the dsRNA viruses the reviewer refers to. Early VACV transcripts, however, are single-stranded, with roughly half the density of double-stranded RNA. To our knowledge, mRNA has been directly visualized only in high-dose single-particle cryo-EM micrographs (as mentioned by Reviewer #1, DOI: 10.1038/s41467-019-10236-7, 10.1016/j.molcel.2025.12.025), and not by cryo-electron tomography, which operates at a small fraction of that dose.

5. Discussion

“tight interaction with the A10-palisade”: not really supported by AF3 or cryo-EM. Any other evidence?

We have removed the word "tight," as our data support the existence of the E6-A10 interaction but not its strength. The interaction itself is nonetheless well supported: five connections between E6 and the surrounding A10 trimers, spanning three different trimers, are resolved in the density (Fig. 4F), and cross-links between E6 and A10 satisfying the DSSO distance constraint independently confirm the proximity of the two proteins (Supplementary

Table 6). We therefore retain the description of the interfaces while avoiding any claim about interaction strength.

"the hexameric architecture of the complex is consistent with a broader theme in biology, where hexameric rings frequently act as translocation or remodeling machines." As far as I know there's nothing special about the C6 symmetry that promotes translocation. There are non-hexameric translocases and (a lot of) hexameric proteins that don't have this function. This would only be a valid argument if evolutionary relatedness was implied, which is not the case here.

We agree that the argument does not hold as stated and have deleted the sentence.

I find it highly implausible structurally that RNA pass through the pore with I8 in the middle. MD simulation may help provide more convincing evidence for or against this model.

We agree that the route of RNA past the central density is not established by our data, and we have revised the text accordingly: we now state only the geometric basis for possible passage – the ~2 nm clearance between the rod and the E6 wall, and the channel dimensions that would accommodate single-stranded RNA – and acknowledge that the precise route, including any requirement for transient axial displacement of the rod, remains to be determined. We agree that molecular-dynamics simulation would be a valuable way to address this. However, a treatment capable of testing RNA transit through an assembly of this size, with a tentatively assigned central component, would require a dedicated computational study rather than an addition to the present work, and we consider it beyond the scope of this manuscript.

Other comments:

- Crosslinking mass spectrometry: are the duplicates technical or biological ones?

These were two biological replicates, originating from two independent batches of vaccinia virion core purification and cross-linking. We have clarified this in the text.

- What do the authors mean by "asymmetric unit of the pore"? If considering the C6 symmetry, this should be smaller than what is shown e.g. in Supp Fig 4.

By "asymmetric unit of the pore" we refer to a single protomer, which was used as the focus of the final refinement. The reviewer is correct that the asymmetric unit itself corresponds to one sixth of the C6 assembly. The map in Supplementary Fig. 5 (originally Supplementary Fig. 4) appears larger than a single protomer because, as the E6 protomer is slightly twisted, we used a cylindrical alignment mask rather than a tight, shaped one conforming to a single subunit; the mask therefore also encompassed parts of the neighboring subunits, which remain visible in the resulting map. The refinement was nonetheless driven by the single asymmetric unit. We have clarified this in the legend of Supplementary Fig. 5 and refer to the mask definition in Supplementary Table 11gradi.

- Reference is missing for EMPIAR-11674 (p. 10).

Thank you for noticing. We have added the missing reference.

Reviewer #1 (Remarks to the Author):

The authors have responded carefully to the previous comments and substantially improved the manuscript. The structural and functional conclusions are now more appropriately separated; I8 is presented as a tentative candidate for the central rod density, and the additional analyses provide stronger support for the proposed E8/E6/L3 architecture. I also appreciate the authors' transparent correction of the rod-displacement analysis and removal of the unsupported claim that rod mobility increases during transcription.

During revision, Calcraft et al. published an independent study, "In situ structure of the poxvirus portal complex," which also identifies E8, E6, and a hexamer-of-dimers arrangement of L3 as the principal components of the poxvirus portal. This reduces the novelty of the molecular assignment but provides strong independent support for the main structural conclusions of the present manuscript. The current work remains valuable because of its complementary XL-MS analysis, systematic candidate testing, automated particle-localization workflow, transcription-state classification, and analysis of the central luminal density.

We thank the reviewer for the positive assessment and for supporting publication. We are also grateful for the careful reading that identified the inconsistencies below, which we have corrected.

Before publication, the authors should cite and discuss the new paper and clarify the similarities and differences between the two studies. In particular, Calcraft et al. report no detectable change in portal architecture after NTP treatment, whereas the present study identifies an uncapped class lacking E8 and L3 densities under transcription conditions. This difference may reflect experimental or classification differences, but it should be discussed directly. The uncapped class should therefore be described as a state observed under the specific transcription conditions used here, rather than as a general consequence of transcription.

Indeed during revision, Calcraft et al. (2026) published an independent cryo-ET study on the vaccinia portal complex from isolated virions. Fortunately, their findings closely agree with ours: they identify the same three viral proteins (E8, E6 and L3) in the same hexameric architecture. E6 forms the central barrel and contacts six A10 trimers, E8 forms the external ring and L3 the bottom ring and forms hexamer of dimers – consistent with the dodecameric, two-ring L3 arrangement we describe. As their study uses a different route, it provides strong support for the architecture reported here. We now cite and discuss this study in the discussion.

We also note the complementary aspects of the two studies. Our work adds cross-linking mass spectrometry, systematic candidate testing across oligomeric states, an automated

particle-localization workflow, classification across transcriptional states, and an analysis of the luminal density. With respect to the luminal density, Calcraft et al. observe the same elongated feature but do not assign it; we propose I8 as a tentative candidate on the basis of our cross-linking and modelling. Their assignment of the helicase D5 concerns a distinct feature – an externally docked hexamer on the E8 rim, associated with genome release and observed in cytoplasmic cores – rather than the luminal density, and is therefore complementary to, rather than in conflict with, our proposal for I8.

Regarding the effect of transcription: Calcraft et al. report no change in portal architecture on addition of NTPs to isolated cores, whereas we observe a class lacking the outer (E8) and basal (L3) ring densities under our transcription conditions. This difference may reflect differences in the treatment protocols, sample preparation or classification approach. In particular, Calcraft et al. performed classification primarily for particle cleaning rather than for exploring particle heterogeneity. Specifically, particles were classified against their previously published portal map, and only particles contributing to well-resolved classes were retained. Thus, particles representing alternative portal architectures that differed substantially from the reference may have been excluded during this selection step. In contrast, in our study, we performed *de novo* classification using initial references generated from random subsets of particles, as described in the Methods, without relying on a pre-existing portal structure as a reference. Moreover, particles were not excluded on the basis of their agreement with a reference structure, as our classification was intended to explore portal architecture rather than to serve as a particle-cleaning step. These differences in classification strategy may have allowed us to sample a broader range of particle states or conformations and could therefore contribute to the different observations between the two studies. Accordingly, we now describe the uncapped class as a state observed under the specific in vitro transcription conditions used here, rather than as a general consequence of transcription.

I have only a few additional minor comments:

The L3 substitution analysis should be described as an in silico AlphaFold3 sensitivity analysis, not as direct experimental validation.

We agree and have clarified throughout that this is an in silico AlphaFold3 sensitivity analysis rather than experimental validation. The relevant sentences in the Results, Methods, and figure legend now describe it as such.

The authors should provide the underlying numbers for the statement that approximately 90% of mapped cross-links satisfy the structural model.

We have added the underlying numbers to the Results. Of the 100 cross-links mapped onto the model, 75 are within the DSSO distance. Of the 25 that exceed it, 16 are A10–

A10 cross-links, which as noted in the manuscript need not originate from the pore complex; excluding these, 75 of 84 (89%) satisfy the constraint. The full distance distribution is provided in the Source Data (SourceData_XLMS_F3_SF6.xlsx).

The number of uncapped class-3 particles is inconsistent between the Results and Figure 5 legend, with 1,467 and 1,397 particles reported, respectively.

We thank the reviewer for catching this. The correct number is 1,397, as given in the Figure 5 legend; the Results text has been corrected accordingly.

Statements that the approximately 2 nm clearance is "sufficient" for mRNA passage should instead state that it is geometrically compatible with passage of single-stranded RNA.

We have revised these statements to say that the ~2 nm clearance is geometrically compatible with the passage of single-stranded RNA, rather than sufficient for it.

Overall, the authors have addressed the major concerns. Subject to incorporation of the newly published study and correction of these minor issues, I support publication.

Reviewer #1 (Remarks on code availability):

The code is well annotated and can be replicated using a notebook. Very nice!

We thank the reviewer for taking the time to examine the code and for the kind comment. We are glad the annotation and notebook make the analysis straightforward to reproduce.

Reviewer #2 (Remarks to the Author):

The revised manuscript resolves some of the concerns on the identification of the pore components through clarification of the mass spectrometry, additional AF modelling and interface analysis such as sequence conservation. A few points remain incompletely addressed:

1/ "the pore-like structures themselves were described only recently (...) The present work is therefore the first to link a specific molecular assembly to this proposed function."

It depends what the authors mean by "recently". On one hand, the pore-like structures were reported in 2005 in the first cryo-ET of virions (10.1073/pnas.0409825102): "We also detected pore-like formations spanning through the viral core membrane, which could be responsible for the export of

newly transcribed RNA to the cytoplasm in the early stages of infection." On the other hand, a paper published after the receipt of the revised manuscript links a specific set of proteins to early mRNA transcription (10.1038/s41586-026-10856-2). These results should be discussed here.

We thank the reviewer for these points, which concern two separate matters.

First: the "first to link a specific molecular assembly to this proposed function" phrasing appeared only in our previous response letter, not in the manuscript, and we make no such claim in the text. We fully agree that pore-like formations were proposed as possible mRNA-export sites as early as 2005 (Cyrklaff et al., 2005), and this work is cited in the original manuscript. Indeed, the contribution of our study is the determination of the molecular composition and architecture of the complex, as described in the manuscript.

Second: during revision we noted the release of the study by Calcraft et al. (2026) and agree it should be discussed, as reviewer 1 also noted. We now cite and discuss this study in the discussion, describing the close agreement between the two independent studies, the complementary aspects of the two approaches, and the difference regarding the effect of NTP-incubation.

2/Modelling:
2a. Oligomeric states: the modelling is a very useful addition to the manuscript. It shows pentameric assemblies with good (or sometimes top) scores. Of course, pentamers are important in introducing curvature in icosahedral and non-icosahedral (e.g. HIV core) capsids. This is worth discussing in the main text, including whether the authors think (or have evidence that) these pentameric objects are present in their imaging dataset or not.

We thank the reviewer for this observation. The pentameric predictions do appear among the modelled oligomeric states, sometimes with high scores; however, we interpret these as reflecting the general tendency of AlphaFold3 to predict plausible closed rings across oligomeric states, rather than as evidence for pentameric assemblies in the biological sample. The role of pentamers in introducing curvature applies to closed, regular lattices such as icosahedral capsids or the HIV core, where the surface must be closed and every lattice position is tiled. The poxvirus palisade is different: it is an irregular lattice on a biconcave core, in which the pore complexes occupy defined positions each surrounded by six A10 trimers, and the surface does not need to close through five-fold vertices. Consistent with this, our reconstructions of the pore complex are six-fold symmetric throughout, validated independently of the reference by in-plane angle randomization (Supplementary Fig. 3), and we find no evidence of pentameric pore assemblies in the data. We have added a sentence to the main text noting this.

2b. Given the resolution of the reconstruction, improving the modelling of E6 would add confidence in the final proposed atomic model. A couple of approaches have not been mentioned:
- Given A10 trimers are proposed to template/constrain the E6 conformation, does their inclusion in AF modelling improve E6 modelling (all neighboring trimers beyond the reasonable token limit but not required).

As suggested, we generated AlphaFold3 predictions of the E6 hexamer together with one and with two A10 trimers. The predictions were not informative: AlphaFold3 did not place the A10 trimers correctly relative to E6. We therefore could not use these predictions to assess whether inclusion of A10 improves the E6 conformation. Representative predictions are shown for the reviewer in below (the remaining predictions were similar). As the reviewer notes, prediction with all six surrounding trimers exceeds a reasonable token limit; those calculations did not complete and are not required.

Given that these predictions are uninformative, we prefer not to include them in the manuscript or the deposited data, and do not discuss them in the text. We would also note that the closed E6 conformation used in our model is supported independently – by the fit to the density, and by the concurrent structure of Calcraft et al. (2026), which likewise resolves a closed E6 hexamer – so it does not rest on co-prediction with A10.

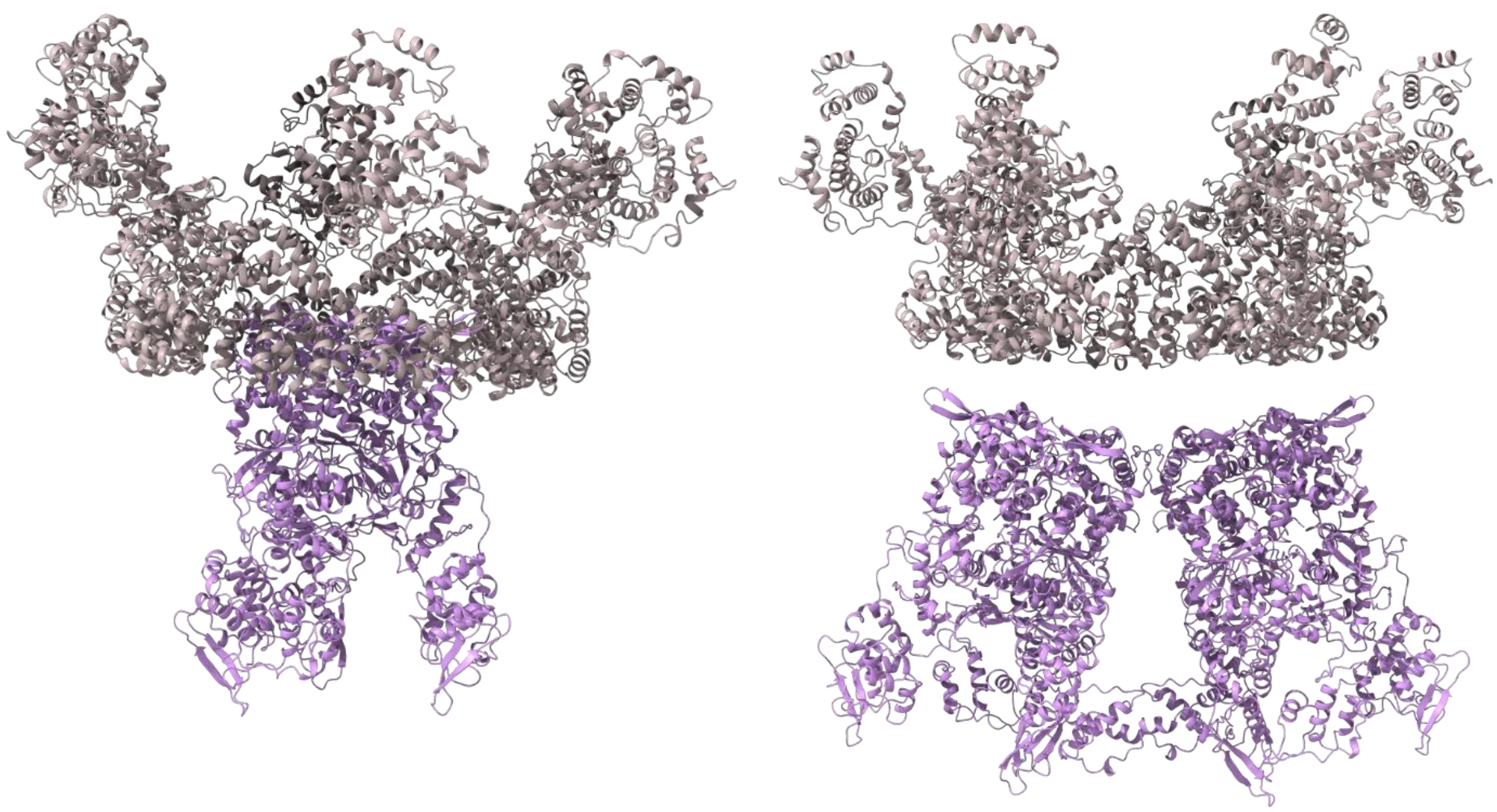

- "E6 hexamer was treated as two rigid bodies corresponding to its two domains.": the comment that E6 seems to be made of 3 domains rather than 2 was not addressed in the authors' response or revised manuscript.

We apologize for not addressing this in the previous round. The reviewer is correct that the E6 PAE shows three blocks. These correspond to three segments delimited by the two flexible boundaries we identified (Lys163-Lys164 and Asn409-Glu410). Structurally, however, the N- and C-terminal segments (the first and third blocks) both form the lower part of the hexamer and fit the density as predicted, while only the intervening middle segment extends beyond the density and required independent placement. We therefore treated the protein as two rigid bodies – the overflowing middle segment, and the N- and C-terminal segments grouped as the lower domain – rather than three. We have revised the text to describe the three PAE segments and this grouping explicitly.

- A potentially better way to approach this, might be to use iterative density-guided modelling which can dramatically improve AF modelling providing more constraints than AFx (e.g. as in PHENIX, 10.1038/s41592-022-01645-6).

We considered density-guided modelling approaches such as that implemented in PHENIX. However, these methods refine atomic coordinates against the map, which is appropriate at near-atomic resolution but not at the ~8 Å resolution of our reconstruction, where individual side chains and backbone conformations are not resolved. Applying such refinement here would introduce atomic detail that the density does not support and risk over-interpretation – precisely the caution we have applied throughout the manuscript in response to the reviewers' earlier comments. We therefore restricted ourselves to rigid-body fitting of predicted models, which is the appropriate level of modelling at this resolution, and we present the result as a fitted model rather than a refined atomic structure. For the same reason, we have not deposited the model in the PDB, which is intended for atomic-resolution structures; the fitted model is instead provided at Zenodo, alongside the density maps deposited in the EMDB.

Other points:

- In response to comment #3 (Reviewer #2), the authors make the interesting point that the core wall presents some density but no distinct structure, and hence is likely to adopt a different symmetry or molecular organization. This should be mentioned in the manuscript.

We have added this observation to the manuscript. The Discussion now notes that the core wall is present as density beneath the pore but does not resolve, indicating that it does not share the six-fold organization of the pore and its surrounding A10 trimers, and is likely irregular or of a different symmetry.

- The channel is described as mostly electropositive except for the domain 1 of E6, which is proposed to interact with I8. This is unlike most RNA/DNA conduits, which

are in general highly electronegative to avoid sticking of the nucleic acid to the channel sides. What is the expected mechanism or molecular feature proposed here to explain this unexpected charge distribution?

We thank the reviewer. First, a clarification: we do not propose that E6 domain 1 interacts with I8, and we do not assign a specific interaction between the luminal density and E6 domain 1.

On the substantive point: the reviewer is right that the channel is predominantly electropositive, which is unusual, since nucleic-acid conduits are typically electronegative so that the RNA backbone does not adhere to the channel walls. We would suggest this is compatible with the pore not being a passive conduit. mRNA export is proposed to be driven by a luminal helicase, an active and directional process; under such motor-driven translocation, transient attraction between the positively charged channel and the RNA backbone need not trap the RNA, as it would in passive diffusion, and the RNA is threaded through actively. We have added a hedged statement of this interpretation to the Discussion, while noting that the mechanism by which single-stranded RNA transits the predominantly positive channel remains to be established.

- L. 596, "Crosslinks further confirmed interactions both within the pore complex and between its components and the A10-palisade, supporting the structural arrangement revealed by STA." This could be toned down since many of the interfaces are not directly validated by crosslinking when poorly modelled structures are excluded.

We have toned down this statement. It now reads that cross-links were consistent with, and provided support for, interactions within the pore complex and between its components and the A10 palisade, rather than confirming them.

- "E6 is also required for MV assembly (Resch et al., 2009), which may relate to the contacts we observe between E6 and the surrounding A10 trimers.": Given the lack of contacts between A10 trimers in the palissade layer, wouldn't the core wall be expected to be driving assembly while A10/E6 control the shape of the particle (cf. comment above on pentamers and Datler et al, 2024)?

We agree this is a plausible interpretation and have revised the text accordingly. Given the absence of direct contacts between A10 trimers in the palisade, the core wall may drive assembly, with the A10 palisade and its contacts to E6 contributing to the shape and organization of the particle rather than to assembly per se. We now present the E6–A10 contacts in this light, referring to Datler et al. (2024).
